# Towards personalized medicine: a scoping review of immunotherapy in sepsis

**DOI:** 10.1186/s13054-024-04964-6

**Published:** 2024-05-28

**Authors:** Marleen A. Slim, Niels van Mourik, Lieke Bakkerus, Katherine Fuller, Lydia Acharya, Tatiana Giannidis, Joanna C. Dionne, Simon J. W. Oczkowski, Mihai G. Netea, Peter Pickkers, Evangelos J. Giamarellos-Bourboulis, Marcella C. A. Müller, Tom van der Poll, W. Joost Wiersinga, in collaboration with the ImmunoSep Consortium, Bart-Jan Kullberg, Aline Nooijer, Frank Veerdonk, Jaap Oever, Jacobien Hoogerwerf, Marlies Hulscher, Mihai Netea, Anke Oerlemans, Athanasios Ziogas, Julie Swillens, Lisa Berg, Nynke Bos, Matthijs Kox, Leda Estratiou, Evangelos Giamarellos-Bourboulis, Antigoni Kotsaki, Antonakos Nikolaos, Gregoriadis Spyros, Thierry Calandra, Sylvain Meylan, Tiia Snaka, Thierry Roger, Michael Bauer, Frank Brunkhorst, Frank Bloos, Sebastian Weis, Willy Hartman, Marleen Slim, Lonneke Vught, Alexander Vlaar, Marcela Muller, Joost Wiersinga, Mihaela Lupse, Grigore Santamarean, Thomas Rimmele, Filippo Conti, Guillaume Monneret, Anna Aschenbrenner, Joachim Schultze, Martina Uelft, Christoph Bock, Robert terHorst, Irit Gat-Viks, Einat Ron, Gal Yunkovitz, Sophie Ablott, Estelle Peronnet, Margaux Balezeaux, Adrien Saliou, Julie Hart, Alexander P. J. Vlaar, Lonneke A. van Vught

**Affiliations:** 1 Department of Intensive Care Medicine, Amsterdam University Medical Center, Meibergdreef 9, Room G3-220, 1105 AZ Amsterdam, The Netherlands; 2 Center for Experimental and Molecular Medicine, Amsterdam University Medical Center, University of Amsterdam, Amsterdam, The Netherlands; 3 Department of Internal Medicine and Radboud Center for Infectious Diseases, Radboud University Medical Center, Nijmegen, The Netherlands; 4 Department of Medicine, McMaster University, Hamilton, Canada; 5 The Guidelines in Intensive Care Development and Evaluation (GUIDE) Group, Research Institute St. Joseph’s Healthcare Hamilton, Hamilton, Canada; 6 Department of Health Research Methods, Evidence, and Impact, McMaster University, Hamilton, Canada; 7 Division of Gastroenterology, McMaster University, Hamilton, ON Canada; 8 Department of Intensive Care Medicine, Radboud University Medical Center, Nijmegen, The Netherlands; 9 4th Department of Internal Medicine, Medical School, National and Kapodistrian University of Athens, Athens, Greece; 10 Department of Internal Medicine, Division of Infectious Diseases, Amsterdam University Medical Center, University of Amsterdam, Amsterdam, The Netherlands

**Keywords:** Sepsis, Immunomodulation, Immunotherapy, Personalized, Enrichment

## Abstract

Despite significant progress in our understanding of the pathophysiology of sepsis and extensive clinical research, there are few proven therapies addressing the underlying immune dysregulation of this life-threatening condition. The aim of this scoping review is to describe the literature evaluating immunotherapy in adult patients with sepsis, emphasizing on methods providing a “personalized immunotherapy” approach, which was defined as the classification of patients into a distinct subgroup or subphenotype, in which a patient’s immune profile is used to guide treatment. Subgroups are subsets of sepsis patients, based on any cut-off in a variable. Subphenotypes are subgroups that can be reliably discriminated from other subgroup based on data-driven assessments. Included studies were randomized controlled trials and cohort studies investigating immunomodulatory therapies in adults with sepsis. Studies were identified by searching PubMed, Embase, Cochrane CENTRAL and ClinicalTrials.gov, from the first paper available until January 29th, 2024. The search resulted in 15,853 studies. Title and abstract screening resulted in 1409 studies (9%), assessed for eligibility; 771 studies were included, of which 282 (37%) were observational and 489 (63%) interventional. Treatment groups included were treatments targeting the innate immune response, the complement system, coagulation and endothelial dysfunction, non-pharmalogical treatment, pleiotropic drugs, immunonutrition, concomitant treatments, Traditional Chinese Medicine, immunostimulatory cytokines and growth factors, intravenous immunoglobulins, mesenchymal stem cells and immune-checkpoint inhibitors. A personalized approach was incorporated in 70 studies (9%). Enrichment was applied using cut-offs in temperature, laboratory, biomarker or genetic variables. Trials often showed conflicting results, possibly due to the lack of patient stratification or the potential influence of severity and timing on immunomodulatory therapy results. When a personalized approach was applied, trends of clinical benefit for several interventions emerged, which hold promise for future clinical trials using personalized immunotherapy.

**Supplementary Information:**

The online version contains supplementary material available at 10.1186/s13054-024-04964-6.

## Background

Despite a global decrease in sepsis burden, sepsis still causes almost 20% of all deaths worldwide [1]. Over the past few decades, significant progress has been made in the understanding of the pathophysiology of sepsis [2], however, treatment is still limited to tackling the pathogens and providing supportive care. To date, limited proven therapies address the underlying mechanisms of this life-threatening condition.

The host response to infection can be dysregulated in multiple ways, resulting in a highly heterogeneous clinical presentation, treatment response, and prognosis [3]. The pathophysiology of sepsis involves dysregulation of the inflammatory response, but also catabolic, metabolic and immune-suppressive features can be present, together resulting in failure to return to homeostasis [3–5]. Modulating these various immune responses to infection represents a promising treatment option. Reason for the numerous failed clinical trials [4–6] could be the use of “one-size-fits-all” approaches, suggesting that personalized immunomodulatory treatment tailored to an individual patient’s immune profile may be a more successful treatment approach. The first step towards implementation of such a personalized strategy is providing a structured and in-depth overview of currently available evidence on immunotherapy in sepsis. The aim of this scoping review is to describe and summarize the literature evaluating immunotherapy in adult patients with sepsis, and to evaluate methods by which a personalized immunotherapy approach has been studied so far.

## Methods

In line with our previously published protocol [7], studies were identified by searching PubMed, Embase, Cochrane CENTRAL and ClinicalTrials.gov from the first paper available until Janaury 29th, 2024. Inclusion criteria were: 1) randomized controlled trials (RCTs) or cohort studies (including case control studies and observational cohorts); 2) investigating immunomodulatory therapies; in 3) adult (≥ 16 years) patients with sepsis, 4) written in English or Dutch. We included studies that addressed therapies with a potential or hypothesized immunomodulatory effect (see Supplementary Methods). Exclusion criteria were: 1) case reports or systematic reviews; 2) animal studies; and 3) studies in healthy volunteers. We deviated from the previously publish protocol [7] by not including studies investigating coronavirus disease 2019 (COVID-19), since immunomodulatory treatments and patient stratification in COVID-19 is explored in a recently published review [8]. The full search strategies, screening and data extraction can be found in the Supplementary Methods. The results are organized in two steps. First, separating observational from interventional studies; and subsequently into treatment groups [3]. Ongoing trials are reported separately. The text in the main paper focusses on randomized controlled trials and studies applying a personalized approach; an overview of observational studies and non-randomized interventional studies not using a personalized approach can be found in the supplement. We defined a personalized approach as the classification of patients into a distinct subgroup or subphenotype. Subgroups are subsets of patients with the same disease or syndrome, based on any cut-off in temperature, laboratory, biomarker or genetic variables. In particular, subgroups based on age, sex or use of certain interventions (mechanical ventilation or vasopressors) were not considered subgroups for a personalized approach. Subphenotypes are subgroups that can be reliably discriminated from other subgroup based on data-driven assessments including machine learning techniques [9]. The use of disease severity scores was not considered as personalized. Individualized interventions were not included, since this review focused on personalized treatments, defined as applying specific treatment at subgroup or subphenotype level, and not at an individualized or patient level.

## Results

### Study characteristics

**Fig. 1:**
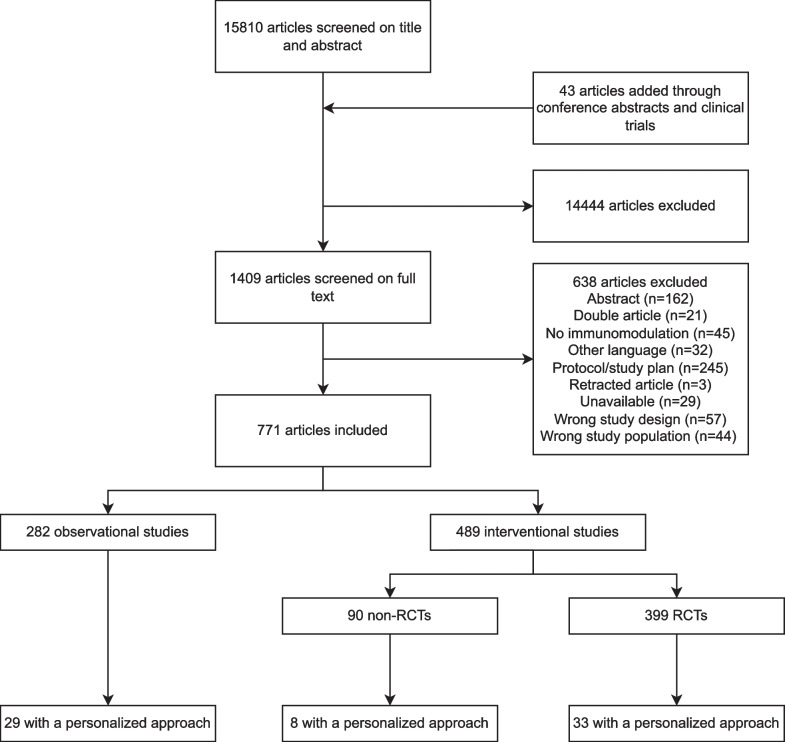
Flow diagram for study selection. RCT, randomized controlled trial

**Fig. 2:**
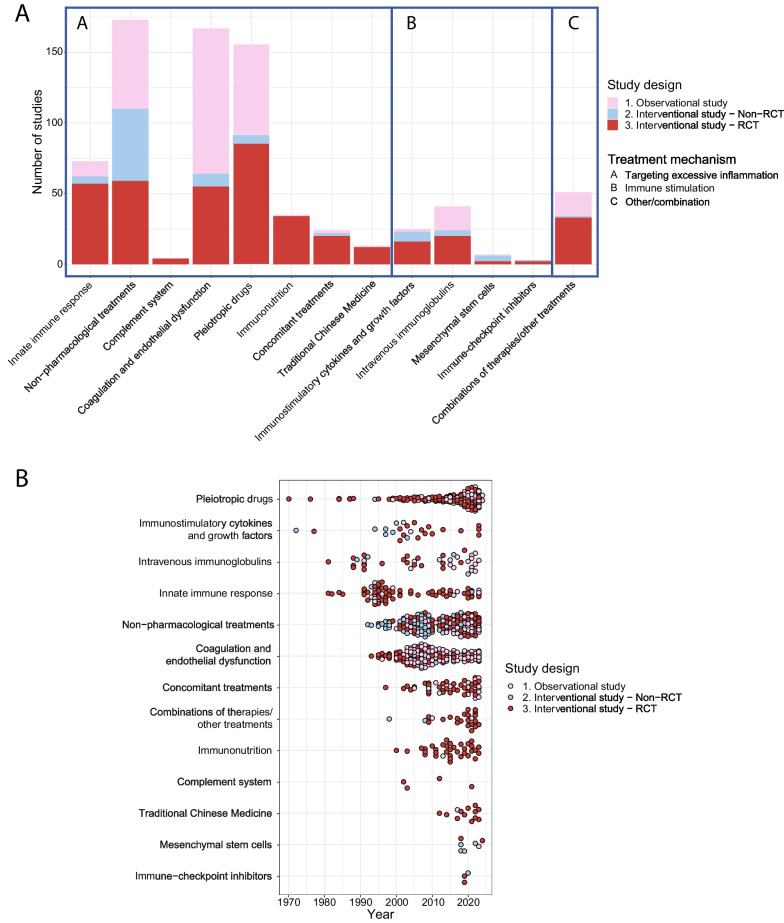
Overview of the included studies. Overview of the included studies in this review **A** divided per treatment strategy, study design and treatment group and **B** divided per year, study design and treatment group demonstrated from the earliest to the latest studies overtime. RCT, Randomized controlled trial

**Fig. 3:**
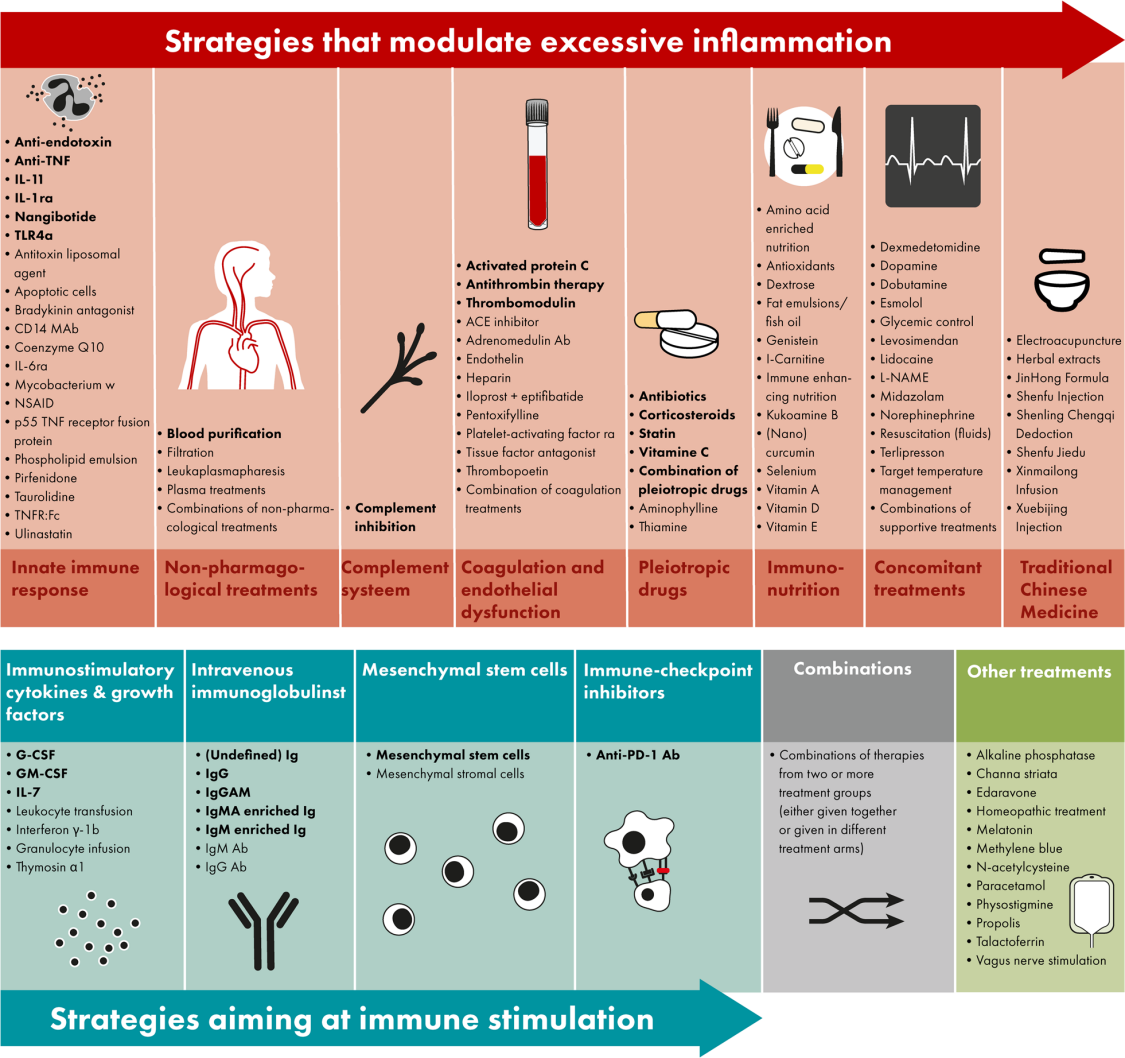
Overview of all immunomodulatory treatments studies in adult patients with sepsis. Immunomodulatory treatments investigated in sepsis patients either modulate excessive inflammation (top op panel, in red) or aim at immune stimulation (bottom of panel, blue), furthermore there are combinations of these treatment strategies (bottom of panel, in grey) or treatments not fitting into these categories (bottom of panel, in green). All treatments displayed in this figure are included in this review; the treatments in bold are discussed in the text and in the supplement, treatment not in bold can be found in the supplement. Abbreviations: Ab, antibody; ACE, angiotensin-converting-enzyme; anti-PD-1, anti-programmed cell death protein 1; G(M)-CSF, granulocyte(-macrophage) colony-stimulating factor; Ig, immunoglobulin; IL, interleukin; L-NAME, L-NG-Nitro arginine methyl ester; NSAID, non-steroidal anti-inflammatory drugs; (r)a, (receptor) antagonist; TLR, toll-like receptor; TNF(r), tumor necrosis factor (receptor)

The search resulted in 15,853 studies, including 43 studies identified through manual searching for the results of protocols, abstracts and registered studies. Our search was completed on January 29th, 2024. Title and abstract screening resulted in 1409 studies that were assessed for eligibility (Fig. 1). In total, 282 observational studies and 489 interventional studies were included (Figs. 1 and 2), of which 70 (9%) applied a personalized approach. Figure 2 depicts a timeline with an overview of the included studies in this review divided by study design, treatment group and year of publication. Figure 3 depicts an overview of all interventions discussed in this review in order to summarize all treatments that have been studied in the research field of immunotherapy in sepsis. Treatments in bold are discussed in the text and in the supplement, treatment not in bold can be found in the supplement.

### Strategies modulating excessive inflammation

#### Innate immune response

Since excessive activation of the innate immune response causes host response dysregulation leading to sepsis, there is a clear rationale to study blocking innate immune activation [3]. Treatments targeting the innate immune response were studied in 11 (15%) observational studies, 5 (7%) non-randomized interventional studies and 57 (78%) RCTs (Supplementary Table 2). Treatments most studied were anti-tumor necrosis factor (TNF)α antibodies (Abs) (n = 17, 23%), anti-endotoxin Abs (n = 14, 19%) and interleukin (IL)-1 receptor antagonists (ra) (n = 10, 14%),

##### RCTs without a personalized approach

Since 1981, RCTs studying anti-endotoxin strategies, including antiserum raised in volunteers immunized with heat-killed mutant *E coli* J5 (murine E5)) and humanized (HA-1A) antibodies directed against the lipid-A part of endotoxin, have been published almost without positive results. Two studies showed a lower mortality in patients with gram-negative bacteremia (30% vs. 49%, n = 197, respectively, 22% vs. 39%, n = 212) [10, 11]. However it has been stated that the results should be interpreted cautiously [12], since this effect was restricted to gram-negative bacteremia and patients most likely to benefit are difficult to identify, and none of the other anti-endotoxin trials showed similar results (Supplementary Table 2). Since 2007, inhibiting Toll-like receptor-4 (TLR4) has been examined using eritoran (a synthetic lipid A antagonist blocking lipopolysaccharide (LPS) from binding at the cell surface MD2-TLR4 receptor) or TAK-242 (a small molecule-inhibitor specific for TLR4) [3]. Neither was found to be effective in large RCTs in severe sepsis patients [13, 14]. Since 1995, anti-TNFα Abs were examined in several trials yielding disappointing results [15, 16]. In the 1990s, Anakinra, a recombinant human IL-1RA, was studied in 6 RCTs, without effect on mortality (Supplementary Table 2).

##### Studies with a personalized approach

 In a retrospective RCT subgroup analysis, showing no survival benefit of anti-TNFα Ab CB0006 in 80 unselected severe sepsis patients, the patients with increased entry TNF-levels appeared to benefit from the high dose anti-TNF Ab (survival rate 86%, n = 7) [17]. When the anti-TNF Ab afelimomab was studied in a phase-III trial including sepsis patients using stratification, only patients with IL-6 levels > 1000 pg/mL had a reduced 28-day mortality (44% vs. 48%, n = 998) [18]. In a similar trial [19], sepsis patients with IL-6 levels > 1000 pg/mL were randomized to receive either afelimomab or placebo, without mortality effect (54% vs. 58%, n = 446). One of the RCTs [20] studying anakinra in 696 sepsis patients, failed to demonstrate a mortality reduction, however in a post-hoc analysis [21] treatment with anakinra provided a 30% decrease of 28-day mortality in patients who, at the start of treatment, had both liver dysfunction and disseminated intravascular coagulation which were interpreted by the authors as traits of macrophage activation. Another post-hoc analysis of this RCT showed that patients with higher baseline IL-1 levels showed mortality reduction compared to patients with lower IL-1 [22]. In an RCT examining IL-11 therapy in patients with thrombocytopenia, a less extensive inflammatory response and lower mortality was observed (31% vs. 14%, n = 105) [23]. In an RCT with patients treated with nangibotide, a triggering receptor expressed on myeloid cells-1 (TREM-1) inhibitor, grouped according to sTREM-1 concentrations at baseline, no improvement in sequential organ failure assessment (SOFA) score was seen [24].

#### Non-pharmacological treatments

The primary non-pharmacological immunotherapy treatment studied in sepsis is blood purification, which may be beneficial through removal of endotoxin, altering cytokine levels, mobilization of cytokines from local tissues, or through more complex processes of immune modulation [25]. Non-pharmacological treatments were studied in 63 (36%) observational studies, 51 (29%) non-randomized interventional studies and 59 (34%) RCTs (Supplementary Table 3). Treatments most studies were blood purification (n = 111, 64%), blood filtration (n = 47, 27%) and plasma treatments (e.g. plasma filtration or exchange, n = 12, 7%).

##### RCTs without a personalized approach

 The clinical effects of these studies are mixed. For instance, while in the EUPHAS trial Polymyxin B hemoperfusion reduced 28-day mortality in 64 patients with severe abdominal sepsis (32% vs. 53%, aHR 0.36; 95% CI 0.16, 0.80) [26], the ABDOMIX trial in peritonitis-induced septic shock did not show a reduction in 28-day mortality (28% vs. 20%, n = 243) [27].

##### Studies with a personalized approach

 The EUPHRATES trial, a multicenter RCT including 450 patients using enrichment by including patients with endotoxin activity assay (EAA) levels ≥ 0.6, did not find improvement in 28-day survival when applying Polymyxin B hemoperfusion [28]. The trial showed that in some septic shock patients the burden of endotoxin activity was extreme (EAA ≥ 0.9). Therefore, a post-hoc analysis of the EUPHRATES trial was conducted in only patients with EAA of 0.6–0.89, not leading to better survival rates [29]. In a retrospective study using the EUPHRATES trial Polymyxin B hemoadsorption was associated with higher 28-day survival in patients with PT-INR > 1.4 or lactate > 3 mmol/L (68% vs. 52%, *p* = 0.02) [30]. Cytokine adsorption and endotoxin hemoabsorption were studied in two observational studies including patients with septic shock and IL-6 ≥ 1000 ng/l, one study found an increased hazard of death of 1.82 (95% CI, 1.03–3.2) compared to a matched control group [31]; the other compared survivors and non-survivors and concluded that this treatment could be beneficial when applied early after onset of shock [32].

#### Complement system

The rationale for studying complement inhibitors is that excessive complement system activation contributes to sepsis-induced organ failure and death [33], which has been studied in 4 RCTs (Supplementary Table 4).

##### RCTs without a personalized approach

Treatment with complement (C)1-inhibitors infusion was studied in three RCTs and associated with reduced all-cause mortality (12% vs. 45% in control, n = 61) [34]. Furthermore, a phase-IIa trial on a monoclonal Anti-C5a antibody in 72 severe sepsis and septic shock patients demonstrated a dose-dependent neutralization of C5a. Complement inhibition was not studied in trials using a personalized approach.

#### Coagulation and endothelial dysfunction

The rationale for studies aiming at coagulation pathways and endothelial dysfunction in sepsis patients is that disseminated intravascular coagulation (DIC) and loss of endothelial barrier integrity are both key phenomena in the pathogenesis of sepsis [2]. Studies aiming at coagulation pathways and endothelial dysfunction were studied in 103 (62%) observational studies, 9 (5%) non-randomized interventional studies and 55 (33%) RCTs (Supplementary Table 5). The most studied treatment interventions were activated protein C (APC; n = 77, 46%), antithrombin (n = 25, 15%) and soluble thrombomodulin (n = 19, 11%).

##### RCT without a personalized approach

In PROWESS, a large phase-III trial in severe sepsis patients, a beneficial effect on 28-day mortality of APC was observed (25% vs. 31%, n = 1690) along with an increased risk of bleeding (3.5% versus 2.0%) [35]. In patients with septic shock, however, the phase-III PROWES-SHOCK trial did not show mortality reduction from treatment with APC (26% vs. 24%, n = 1697) [36]. Even though antithrombin therapy resulted in improvement of DIC [37, 38], it did not result in a decreased mortality in patients with severe sepsis or septic shock (39% vs. 39%, n = 2314) [39]. In the SCARLET trial soluble thrombomodulin did not reduce mortality in unselected sepsis patient (27% vs. 29%, n = 800) [40].

##### Studies with a personalized approach

In a predefined subgroup analyzing patients with severe protein C deficiency from the PROWESS-SHOCK, APC treatment did not result in differences in 28-day mortality (28.7% vs 30.8%, n = 673) [36]. However, in a retrospective cohort study in 48 patients with severe sepsis and elevated troponin, treatment with APC did improve intensive care unit (ICU)-mortality (30% vs. 72%, n = 48) [41]. A post hoc analysis of the SCARLET trial showed that patients with higher baseline thrombin generation biomarker levels showed reduced mortality when treated with recombinant human soluble thrombomodulin [42]. A study using coagulation phenotypes as a secondary analysis of multicenter registries on sepsis patients admitted to the ICU, demonstrated that in one in four phenotypes, the one with high fibrinogen/fibrin-degradation-products and D-Dimer, treatment with thrombomodulin was associated with lower mortality (adjusted risk difference -18%, 95% CI -29%,-7%, n = 323) [43]. Antithrombin supplementation therapy only reduced in-hospital mortality in sepsis patients with very low anthithrombin activity (HR 0.603, 95% CI 0.368, 0.988) [44]. When applying molecular phenotypes previously identified in acute respiratory distress syndrome (ARDS) different treatment response to activated protein C were found, with survival benefit in the hyperinflammatory and harm in the hypoinflammatory phenotype [45].

#### Pleiotropic drugs

Pleiotropic drugs refer to substances exerting effects other than for which it was initially developed. Corticosteroids (n = 89, 57%) and antibiotics (n = 18, 12%, mainly macrolides are known for their immunomodulatory effect [46, 47]) are the primary pleotropic drugs used in sepsis (Supplementary Table 6). Pleiotropic drugs were studied in 64 (41%) observational studies, 6 (4%) non-randomized interventional studies and 85 (55%) RCTs.

##### RCTs without a personalized approach

Corticosteroids have been studied in sepsis patients in over 30 RCTs with contradicting results. In septic shock patients receiving hydrocortisone plus fludrocortisone compared to placebo, mortality was lower (43% vs. 49%, n = 1241) [48], however hydrocortisone alone in septic shock patients undergoing mechanical ventilation did not result in the same effect (28% vs. 29%, n = 3658) [49]. A recently published RCT showed a lower 28-day mortality among ~ 800 patients with severe community-acquired pneumonia treated in the ICU with hydrocortisone (12% vs. 6%) [50]. In an RCT in sepsis patients receiving vasopressors those who received intravenous vitamin C had a higher risk of death or persistent organ dysfunction (44.5% vs 38.5%, n = 872) [51]. Trials investigating the combination of vitamin C, thiamine, and hydrocortisone did not find positive results on ventilator-free-days [52] or mortality SPS:refid::bib53|bib54(53, 54). Treatment with clarithromycin, next to standard-of-care antimicrobial treatment, resulted in contradicting findings, the latest large trial did, however, found an association with a decreased 90-day mortality compared to placebo (43% vs. 60%, n = 200) [55].

##### Studies with a personalized approach

In an RCT including patients with severe community-acquired pneumonia and C-reactive protein (CRP) > 150 mg/L methylprednisolone led to reduced treatment failure (development of shock, need for mechanical ventilation or death) compared to placebo (31% vs. 13%, n = 60) [56]. Increased mortality was observed in patients with sepsis response signature-(SRS)2 endotype compared to SRS1 in patients treated with hydrocortisone (n = 176, OR 7.9, 95% CI 1.6, 39.9) [57]. When assigning patients to two previously identified gene expression-based endotypes, corticosteroid exposure may be associated with increased mortality among septic shock endotype A patients (OR 3.1, 95% CI, 1.0 – 9.6, n = 97) [58]. When gene expression scores used to identify the immune state of shock patients; patients with the prevalent immune-adaptive state may be harmed by hydrocortisone [59]. Expression of *GLCCI1* was associated with decreased time to shock reversal, and the expression of *BHSD1* was associated with increased time to shock reversal (n = 494, HR 3.81 vs. 0.64 and HR 0.55 vs. 1.32, respectively) [60]. In two cohorts with > 1200 and > 2500 patients, studying the use of machine learning for corticosteroid treatment decision showed positive results [61, 62]. Another cohort study employing machine learning identified interferon (IFN)γ/IL10 as a theranostic marker; a low serum IFNγ/IL10 ratio predicted increased survival in the hydrocortisone group whereas a high ratio predicted better survival in the placebo group [63]. One post-hoc analysis of an RCT examined the effect of simvastatin in sepsis-induced ARDS in patients with high baseline IL-18, which was associated with a higher survival probability (39% vs. 24%, n = 511) [64].

#### Immunonutrition, concomitant treatments and traditional chinese medicine

See the Supplementary Results for the studies regarding immunonutrition (Supplementary Table 7), concomitant treatments (Supplementary Table 8) and Traditional Chinese Medicine (Supplementary Table 9).

### Strategies aiming at immune stimulation

#### Immunostimulatory cytokines and growth factors

Immunostimulatory cytokines and growth factors have been studied in 2 (8%) observational studies, 7 (28%) interventional non-RCTs and 16 (64%) RCTs (Supplementary Table 10). Treatments most studies were granulocyte-colony stimulating factor (G-CSF) (n = 11, 44%) and granulocyte–macrophage colony-stimulating factor (GM-CSF) (n = 7, 28%).

##### RCTs without a personalized approach

 Six RCTs investigating G-CSF did not show an effect on mortality (Supplementary Table 10). Even though likewise no survival benefit was found for GM-CSF, one RCT did demonstrate improved respiratory function (n = 18) [65].

##### Studies with a personalized approach

 Three RCTs studied biomarker-guided (human leukocyte antigen DR (HLA-DR) < 8000) GM-CSF treatment; one trial resulted in a shorter time of mechanical ventilation (148 ± 103 h vs. 207 ± 58 h, n = 38) [66]; another in decreased indoleamine 2,3-dioxygenase levels, possibly due to an improved antibacterial defense (35.4 ± 21.0 vs 21.6 ± 9.9 (baseline vs day 9), n = 36) [67]; another had no effect on the prevention on ICU-acquired infections (11% vs 11%, n = 98) [68]. In an RCT studying intramuscular recombinant human IL-7 (CYT107) in 27 patients with severe lymphopenia, CYT107 reversed the loss of CD4+ and CD8+ cells [69].

#### Intravenous immunoglobulins

Immunoglobulins can opsonize and neutralize pathogens and toxins resulting in immunostimulation and reduced inflammation. Immunoglobulins have been studied in 17 (41%) observational studies, 4 (10%) interventional non-RCTs and 20 (49%) RCTs (Supplementary Table 11).

*RCTs without a personalized approach* RCTs on immunoglobulins demonstrate contradicting results concerning improving patient outcome and decreasing mortality (Supplementary Table 11). For example, two RCTs on immunoglobulin (Ig)G demonstrated a lower mortality from septic shock in one trial (38% vs. 67%, n = 62) [70]; while no mortality reduction was seen in another trial (37% vs. 39%, n = 653) [71].

*Studies with a personalized approach* In an observation study intravenous immunoglobulins (IVIG) administration in patients with sepsis and low serum IgG levels was associated with improved prognosis (OR 0.15; 95%CI, 0.04–0.54; n = 87) [72]. In sepsis patients with neutropenia, polyclonal immunoglobulin M-enriched immunoglobulins led to a decrease in endotoxin levels in survivors, in non-survivors this was not seen [73]. In a post-hoc RCT analysis, a reduction of all-cause mortality was observed in pneumosepsis patients with high CRP and low IgM levels when administered trimodulin (polyclonal antibody) (reduction of 25%, n = 92) [74]. In an RCT studying the use of IVIG, IGMA had no effect on 28-day mortality in neutropenic patients (26% vs. 28%, n = 211) [75].

#### Mesenchymal stem cells

Mesenchymal stem cells enhance bacterial clearance and modulate the immune response. Mesenchymal stem cells have been studied in 1 (17%) observational study, 4 (67%) non-randomized interventional studies and 1 (17%) RCT (Supplementary Table 12).

##### RCTs with a personalized approach

One RCT showed that mesenchymal stem cells are safe and attributed to the faster hemodynamic stabilization in 30 patients with neutropenia [76].

#### Immune-checkpoint inhibitors

Immune-checkpoint inhibitors have been studied in 1 (33%) non-randomized interventional study and 2 (67%) RCTs (Supplementary Table 13).

*Studies with a personalized approach* Immune-checkpoint inhibitors, like anti-programmed death (PD)-1 antibodies, while not yet proven to enhance survival, also appear safe and could improve immune recovery in one non-randomized interventional study and two RCTs with patients with absolute lymphocyte count ≤ 1.1 × 10^3^ cells/μL [77–79].

### Combination of therapies and other therapies

See the Supplementary Results for studies investigating combination of therapies (Supplementary Table 14) and treatments that could not be classified into the previously mentioned treatment groups (Supplementary Table 15).

### Ongoing trials

A search on ClinicalTrials.gov yielded 78 sepsis studies, reflecting ongoing research across the entire immunotherapy spectrum (Supplementary Table 16). Notably, sixteen studies (21%) implement a personalized approach. For the RCTs (n = 14) applying a personalized approach see Table 1. One of these trials, employing a double-dummy design, is studying the impact of precision immunotherapy on sepsis phenotypes like hyperinflammation (using very high ferritin levels as a marker for macrophage activation-like syndrome (MALS)) and immunoparalysis (using low expression of HLA-DR on monocytes as marker of immunoparalysis) [80]. Patients, stratified by biomarkers are assigned to receive either placebo or active immunotherapy as an adjunct to standard care. The active treatments include anakinra for MALS and interferon-gamma for immunoparalysis.

**Table 1:** Overview of ongoing RCTs in sepsis patients applying a personalized approach

Treatment group	Treatment	Recruitment status	Number of patients	Setting	Personalized approach	Sepsis definition	Population	Endpoint	NCT number
Strategies that modulate excessive inflammation									
Innate immune response	Anakinra	Recruiting	60	Not specified	Prepepsin > 350 pg/ml	Adjusted sepsis-3 (quick SOFA)	Pneumonia	SOFA score	NCT05785442
Non-pharmalogical	CytoSorb	Recruiting	160	Not specified	IL-6 ≥ 1000 ng/l and need for CRRT	Not specified	General	Change in noradrenaline	NCT04963920
	CytoSorb	Recruiting	32	Not specified	IL-6 ≥ 1000 ng/l and need for CRRT	Not specified	General	Catecholamine dose	NCT04013269
	Hemoperfusion with polymyxin B	Recruiting	20	Not specified	PCT > 2ng/mL, CRP > 150mg/L and EAA > 0.6	Sepsis-3, shock	General	Endotoxin activity level, vasopressors	NCT04920565
	Hemoperfusion with polymyxin B	Recruiting	150	Not specified	EAA ≥ 0.60 to < 0.90	Not specified	Cardiac surgery patients	Mortality	NCT03901807
Endothelial dysfunction	Recombinant Human Thrombopoietin	Recruiting	200	ICU	Platelets < 50 × 10^9/L	Sepsis-3	General	Mortality	NCT02707497
Pleiotropic drugs	Fludrocorti-sone and/or hydrocortisone	Recruiting	1800	ICU	Biomarker-guided, adaptive Bayesian design	Not specified	CAP	Mortality, persistent organ dysfunction	NCT04280497
	High-dose vitamin C	Recruiting	152	ICU	PCT ≥ 2 ng/ml	Sepsis-3	General	Mortality	NCT05194189
Immunonutrition	Smoflipid	Recruiting	68	ICU	Total cholesterol ≤ 100 mg/dL or HDL-C + LDL-C ≤ 70 mg/dL	Sepsis-3	General	Maximum tolerated dose, total cholesterol	NCT03405870
	Xanthohumol	Recruiting	50	Not specified	PCT > 5 ng/ml and IL-6 > 100 pg/ml	Not specified	General	Mortality, length of stay, inflammatory cytokines, glycocalyx damage	NCT06225258
Concomitant treatments	Human albumin	Not yet recruiting	100	ICU	Albumin plasma level < 35g/L and lymphocytes count < 1,100 cel/mL	Sepsis-3, shock	CAP, urinary, skin or biliary infection	B cell response	NCT05645887
Strategies aiming at immune stimulation									
Immunostimulatory cytokines & growth factors	Interferon-γ	Recruiting	132	ICU	HLA-DR < 8000 antibody/cell	Not specified	Ventilator-associated pneumonia	Ventilation-free days	NCT05843786
Intravenous immunoglobulins	Pentaglobulin	Recruiting	200	Not specified	IL-6 is ≥ 1000 pg/ml	Not specified	Peritonitis	Multiple organ failure	NCT03334006
Combinations									
Combination of therapies	Anakinra or Interferon-γ	Active, not recruiting	280	Not specified	Patients with hyperinflammation receive anakinra, patient with immunoparalysis interferon-gamma	Sepsis-3	Pneumonia or blood stream infection	SOFA score	NCT04990232

Ongoing trials registered on ClinicalTrails.gov on January 29, 2024 investigating immunomodulatory treatments in adult patients with sepsis using a personalized approach

*CAP* Community-acquired pneumonia; *CRRT* Continuous renal replacement therapy; *EAA* Endotoxin activity assay; *HDL-C* High-density lipoprotein cholesterol; *HLA-DR* Human Leukocyte Antigen – DR; *ICU* Intensive care unit; *IL* Interleukin; *LDL-C* Low-density lipoprotein cholesterol; *NCT* National Clinical Trial; *PCT* Procalcitonin; *RCT* Randomized controlled trial; *SOFA* Sequential organ failure assessment

## Discussion

This scoping review provides a comprehensive overview of immunomodulatory treatments investigated in adult patients with sepsis, highlighting studies with a personalized treatment approach. Our results show that trials often showed conflicting results. Possibly due to the lack of patient stratification, requiring the need to confirm positive findings in large multicenter populations or the potential influence of severity and timing on immunomodulatory therapy results. Several immunomodulatory treatments described in this review suggest possible efficacy, laying the groundwork for future trials to demonstrate their effectiveness. If a personalized approach is applied, clinical benefits of treatment appear to emerge in several studies. This emphasizes the need to decipher different host response endo-/phenotypes for the intervention to modulate.

Over 700 studies investigating immunotherapy in patients with sepsis have been performed and despite this body of evidence, the 2021 surviving sepsis campaign guidelines only include intravenous hydrocortisone as an immunomodulatory treatment for patients with vasopressor refractory septic shock [81]. Perhaps patient stratification might be the way forward, in which we have witnessed notable advancements in recent years, including therapies targeted by biomarker measurements. For instance, the ratio of IFN-γ to IL-10 has been used to guide corticosteroid therapy decisions [63], while HLA-DR levels on monocytes and plasma IL-10 concentrations have been used for stratification of treatment with either GM-CSF or IFN-γ [82]. In ongoing and upcoming sepsis trials, an increase in patient stratification has been observed. The personalized approach most applied is a cut-off value for inflammatory markers such as IL-6 or procalcitonin. Two studies use more complex stratification methods. One is the ImmunoSep trial (NCT04990232) which uses biomarker stratification to identify patients with either hyperinflammation or immunoparalysis [80]. Another example is the RECORDS trial, which aims at defining endotypes in sepsis adults associated with responsiveness to corticosteroids [83]. This multicenter, placebo-controlled, biomarker-guided, adaptive Bayesian design basket trial will randomly assign 1800 adults to a biomarker stratum to identify resistant or sensitive sepsis to corticosteroid treatment. In our opinion, using biomarker-based protocols for patient stratification will be the way forward in sepsis research.

The strengths of this review include the systematic search and comprehensive inclusion of all studies investigating immunomodulatory treatments in sepsis, including studies on immunonutrition, Traditional Chinese Medicine and concomitant treatments. Furthermore, it gives an extensive overview of studies that used a personalized approach, which can be used as the foundation for new study designs and aims. A few limitations should be mentioned. Given that the objective of this review was to provide a comprehensive overview of all studies examining immunomodulatory treatments in sepsis, a scoping review was considered the most suitable approach. Consequently, a risk of bias assessment was not conducted [84]. Although inevitable, different criteria for sepsis have been used over time [85], leading to heterogeneity in the population included. In this review treatments are considered personalized when a subgroup of subphenotype was selected based on biological characteristics possibly making the patient benefit more from a specific treatment, however, the is no uniform definition for ‘personalized medicine’. Since no qualitative methods such as qualitative text analysis, evidence maps or evidence gap maps, were deployed, no information on the research gaps in the field could be given. Due to the extensive body of evidence, we refrained from reporting cohort studies in a structured manner in the main text. Even though these observational studies and non-randomized interventional studies were included in the supplementary materials, not discussing them in the main text of the paper could be perceived as selective reporting bias. Lastly, since the search yielded a large amount of studies there was only limited possibility for in-depth description of important trials, including descriptions of the different dosages given.

## Conclusions

Decades of extensive investigation into immunomodulatory treatments has led to over 700 studies investigating these treatment for sepsis, with often conflicting results. The lack of therapeutic efficacy appears to be related to the difficulty to enroll the right patients for the intervention. Since it is highly unlikely that one single immunomodulatory treatment will be universally effective in all sepsis patients, a personalized approach seems the way forward. To date, only a small proportion of studies have looked into enrichment strategies in sepsis, and for several interventions the therapeutic efficacy appears to emerge when a personalized approach was used. Patient stratification will play a pivotal role in the identification of patients that may benefit from targeted immunotherapy.

### Supplementary Information

Supplementary material 1.

## Data Availability

Not applicable.

## Abbreviations

Abs: Antibodies
APC: Activated protein C
ARDS: Acute respiratory distress syndrome
C: Complement
COVID-19: Coronavirus disease 2019
CRP: C-reactive protein
DIC: Disseminated intravascular coagulation
EAA: Endotoxin activity assay
G-CSF: Granulocyte-colony stimulating factor
GM-CSF: Granulocyte–macrophage colony-stimulating factor
HLA-DR: Human leukocyte antigen DR
ICU: Intensive care unit
IL: Interleukin
INF: Interferon
(IV)Ig: (Intravenous) immunoglobulins
LPS: Lipopolysaccharide
MALS: Macrophage activation-like syndrome
PD-1: Programmed death-1
Ra: Receptor antagonists
RCT: Randomized controlled trial
SOFA: Sequential organ failure assessment
TLR: Toll-like receptor
TNF: Tumor necrosis factor
TREM-1: Triggering receptor expressed on myeloid cells-1
