# Supplementary material for: Towards personalized medicine: a scoping review of immunotherapy in sepsis
Source: Crit Care. 2024 May 28;28:183. doi: 10.1186/s13054-024-04964-6 (PMC11134696; doi:10.1186/s13054-024-04964-6)
Supplement: Supplementary file 1 — Supplementary material 1. [file 13054_2024_4964_MOESM1_ESM.docx]

**Online Data Supplement**

**Towards personalized medicine: a scoping review of immunotherapy in sepsis**

Marleen A. Slim*, Niels van Mourik*, Lieke Bakkerus, Katherine Fuller, Lydia Acharya, Tatiana Giannidis, Joanna C. Dionne, Simon J.W. Oczkowski, Mihai G. Netea, Peter Pickkers, Evangelos J. Giamarellos-Bourboulis, Marcella C.A. Müller, Tom van der Poll, W. Joost Wiersinga, in collaboration with the ImmunoSep Consortium, Alexander P.J. Vlaar^, Lonneke A. van Vught^

*^ Both authors contributed equally.

| **Content** | **Page** |
| --- | --- |
| Supplementary Methods. | 2 |
| Supplementary Results. | 7 |
| Supplementary Table 1. Data-extraction form | 10 |
| Supplementary Table 2. Innate immune response | 11 |
| Supplementary Table 3. Non-pharmacological treatments | 16 |
| Supplementary Table 4. Complement system | 19 |
| Supplementary Table 5. Coagulation and endothelial dysfunction | 20 |
| Supplementary Table 6. Pleiotropic drugs | 25 |
| Supplementary Table 7. Immunonutrition | 30 |
| Supplementary Table 8. Concomitant treatments | 32 |
| Supplementary Table 9. Traditional Chinese Medicine | 34 |
| Supplementary Table 10. Immunostimulatory cytokines and growth factors | 35 |
| Supplementary Table 11. Intravenous immunoglobulins | 37 |
| Supplementary Table 12. Mesenchymal stem cells | 39 |
| Supplementary Table 13. Immune-checkpoint inhibitors | 40 |
| Supplementary Table 14. Combinations of therapies | 41 |
| Supplementary Table 15. Other therapies | 45 |
| Supplementary Table 16. Ongoing trials | 47 |
| References | 54 |

**Supplementary Methods.**

*The full search string*

**PubMed**

("Sepsis"[Majr] OR sepsis[tiab] OR septic[tiab])

AND

("Immunotherapy"[Mesh] OR "Precision Medicine"[Mesh] OR "Immunosuppression"[Mesh] OR "Anti-Inflammatory Agents/therapeutic use"[Mesh] OR "Anti-Infective Agents/therapeutic use"[Mesh] OR "Glucocorticoids"[Mesh] OR "Hydroxycorticosteroids"[Mesh] OR "Anticoagulants/therapeutic use"[Mesh] OR "Cytokines/antagonists and inhibitors"[Mesh] OR "Cytokines/therapeutic use"[Mesh] OR "Granulocyte Colony-Stimulating Factor/therapeutic use"[Mesh] OR "Granulocyte-Macrophage Colony-Stimulating Factor/therapeutic use"[Mesh] OR "Immunoglobulins, Intravenous/therapeutic use"[Mesh] OR "Programmed Cell Death 1 Receptor/therapeutic use"[Mesh] OR "Toll-Like Receptors/therapeutic use"[Mesh] OR "Polymyxin B/therapeutic use"[Mesh] OR "Thymosin/therapeutic use"[Mesh] OR "Protein C/therapeutic use"[Mesh] OR "Mesenchymal Stem Cells"[Mesh] OR immunotherap*[tiab] OR interleukin*[tiab] OR interferon[tiab] OR toll-like-receptor agonist*[tiab] OR anti-endotoxin compound*[tiab] OR blocking complement*[tiab] OR activated vitamin C[tiab] OR mesenchymal stem cell*[tiab] OR immune checkpoint inhibitor*[tiab] OR extracorporeal blood removal[tiab] OR corticosteroid*[tiab])

AND

("Clinical Trial" [Publication Type] OR clinical trial*[tiab] OR randomized trial*[tiab] OR randomized trial*[tiab] OR controlled trial*[tiab])

NOT

(("Child"[Mesh] OR "Infant"[Mesh] OR "Pediatrics"[Mesh] OR child*[tiab] OR infan*[tiab] OR newborn*[tiab] OR neonat*[tiab] OR baby[tiab] OR babies[tiab] OR pediatr*[tiab] OR paediatr*[tiab]) NOT "Adult"[Mesh])

NOT

("Editorial"[Publication Type] OR "Letter"[Publication Type] OR "News"[Publication Type] OR "Comment"[Publication Type] OR "Review" [Publication Type] OR "Case Reports"[Publication Type] OR "letter*"[Title] OR "comment*"[Title] OR editorial[ti])

NOT

(("Animals"[MeSH Terms] OR "Animal Experimentation"[MeSH Terms] OR "animals, laboratory"[MeSH Terms] OR "models, animal"[MeSH Terms] OR "animal*"[Title/Abstract] OR "rat"[Title/Abstract] OR "rats"[Title/Abstract] OR "mice"[Title/Abstract] OR "mouse"[Title/Abstract] OR "dog"[Title/Abstract] OR "dogs"[Title/Abstract] OR "pig"[Title/Abstract] OR "pigs"[Title/Abstract] OR "cow"[Title/Abstract] OR "cows"[Title/Abstract] OR "monkey"[Title/Abstract] OR "monkeys"[Title/Abstract] OR "horse"[Title/Abstract] OR "horses"[Title/Abstract] OR "sheep"[Title/Abstract] OR "ovine"[Title/Abstract] OR "lamb"[Title/Abstract] OR "lambs"[Title/Abstract] OR "goat*"[Title/Abstract] OR "swine"[Title/Abstract] OR "porcine"[Title/Abstract] OR "pup"[Title/Abstract] OR "pups"[Title/Abstract] OR "canine"[Title/Abstract] OR "bitch*"[Title/Abstract] OR "beagle"[Title/Abstract] OR "feline"[Title/Abstract] OR "rodent*"[Title/Abstract] OR "rabbit*"[Title/Abstract] OR "murine"[Title/Abstract] OR "ape"[Title/Abstract] OR "apes"[Title/Abstract] OR "gorilla"[Title/Abstract] OR "gorillas"[Title/Abstract] OR "catfish"[Title/Abstract]) NOT "Humans"[MeSH Terms])

[**Cochrane Central Register of Controlled Trials**](https://www.cochranelibrary.com/)

ID Search Hits

#1 (sepsis or septic):ti,ab,kw

#2 (immunotherap* OR interleukin* OR interferon OR toll-like-receptor agonist* OR anti-endotoxin compound* OR blocking complement* OR activated vitamin C OR mesenchymal stem cell* OR immune checkpoint inhibitor* OR extracorporeal blood removal OR corticosteroid*):ti,ab,kw

#3 (precision medicine OR immunosuppress* OR anti-nflammatory agent* OR anticoagulant* OR cytokines OR granulocyte colon stimulating factor* OR granulocyte macrophage colony-stimulating factor OR immunoglobulin* OR programmed cell death 1 receptor* OR Toll-Like Receptors OR polymyxin B OR thymosin OR protein OR anti-infective agent* OR glucocorticoid* OR hydroxycorticosteroid* OR mesenchymal stem cell*):ti,ab,kw

#4 #2 or #3

#5 #1 and #4 in Trials

**ClinicaTrials.gov**

Studies found for: Interventional Studies | sepsis | Adult, Older Adult

**EMBASE (Ovid):**

Database(s): Embase Classic+Embase 

**Search Strategy EMBASE:**

| **#** | **Searches** |
| --- | --- |
| **1** | exp *sepsis/dt, th or (sepsis or septic).ti,ab,kw. |
| **2** | exp immunotherapy/ or personalized medicine/ or exp immunosuppressive treatment/ |
| **3** | exp antiinflammatory agent/dt, th or exp antiinfective agent/dt, th or drotrecogin/ or exp corticosteroid/dt, th or exp anticoagulant agent/dt, th or exp cytokine/dt or granulocyte colony stimulating factor/dt or granulocyte macrophage colony stimulating factor/dt or exp immunoglobulin/dt, th or programmed death 1 receptor/dt or toll like receptor/dt or polymyxin B/ or thymosin/dt or exp mesenchymal stem cell/ |
| **4** | ((immunotherap* or interleukin* or interferon or toll-like-receptor agonist* or anti-endotoxin compound* or blocking complement* or activated vitamin C or mesenchymal stem cell* or immune checkpoint inhibitor* or extracorporeal blood removal or corticosteroid*) and (treat* or therap*)).ti,ab,kw. |
| **5** | 2 or 3 or 4 |
| **6** | exp clinical trial/ or controlled clinical trial/ or controlled study/ or randomized controlled trial/ |
| **7** | ((randomized or randomised or controlled) adj3 trial*).ti,ab,kw. |
| **8** | clinical trial*.ti,ab,kw. |
| **9** | 6 or 7 or 8 |
| **10** | 1 and 5 and 9 |
| **11** | (exp child/ or exp pediatrics/ or (child* or infan* or newborn* or neonat* or baby or babies or pediatr* or paediatr*).ti,ab.) not adult/ |
| **12** | 10 not 11 |
| **13** | (exp animal/ or exp animal experiment/ or exp animal model/ or exp veterinary medicine/ or (animal* or monkey* or sheep or ovine or lamb or lambs or goat* or pig or pigs or swine or porcine or pup or pups or dog or dogs or canine or bitch* or beagle or feline or rodent* or rabbit* or rat or rats or mice or mouse or murine or cow or cows or horse or horses or ape or apes or gorilla or gorillas).ti,ab,kw.) not human/ |
| **14** | 12 not 13 |
| **15** | letter/ or editorial/ or note/ or case report/ or conference paper/ or "review"/ or (letter or comment* or editorial or case report).ti. |
| **16** | 14 not 15 |

*Therapies with a potential or hypothesized immunomodulatory effect*

Therapies with a hypothesized immunomodulatory effect include nutrition, inotropic drugs and vasopressors, macrolides, resuscitation strategies, sedatives, statins, and target temperature management, if they specifically investigated and reported the immunomodulatory effects of the treatment in one of the following two ways: 1) by literally stating that the authors were interested in the treatment effect on the immune system or 2) by mentioning the effect on (inflammatory) cell populations, protein biomarkers and/or immune assays.

*Screening and data-extraction*

Two authors (MS, NvM) independently screened titles and abstracts of all identified studies, followed by the full texts. Disagreement between the two assessors was resolved until consensus. Data-extraction was performed by nine authors (MS, NvM, LB, KF, LA, TG, JCD, SJWO, LvV). Data from included studies were charted in the predesigned electronic data extraction form that can be found in **Supplementary Table 1**. Data-extraction of ten percent of the included studies was checked by another author to ensure interrater consistencty. In the initial protocol we planned to appraise the quality of all individual studies, however, the large number of included studies precluded this (1). A snowball approach (2) finding additional studies in the reference lists of included articles was deemed not relevant nor feasible due to the extensive number of studies included.

**Supplementary Results**

*Immunonutrition*

The gut surface, containing a large collection of immune cells, has an impaired defense mechanism during infections. Delivery of substances for enterocytes to improve the immune response is an attractive hypothesized effect of immunonutrition (3). Immununutrition is studied in 1 (3%) observational study and 34 (97%) RCTs (**Supplementary Table 7**). Treatments most studied were selenium (n=9, 27%) and fat emulsions and/or fish oil (n=6, 18%).

*RCTs without a personalized approach –* Fish oil treatment resulted in lower inflammatory markers in 4 RCTS (**Supplementary Table 7**) but showed conflicting results on mortality in 2 RCTs (n=25-60) (4, 5). In all identified RCTs examining selenium, treatment did not lead to mortality reduction (**Supplementary Table 7**) (6). No studies investigating immunonutrition used stratification.

*Concomitant treatments*

The hypothesized effect of concomitant treatments is to support the immune system and restore homeostasis (7). Concomitant treatments as adjunctive therapy in sepsis is studied in 2 (8%) observational studies, 2 (8%) interventional non-RCTs and 20 (83%) RCTs (**Supplementary Table 8**). Treatments most studied included dexmedetomidine (n=6, 25%) and levosimendan (n=4, 17%).

*RCTs without a personalized approach –* In all identified RCTS studying dexmetomidine sedation, it resulted in decreased inflammatory biomarkers (**Supplementary Table 8**), in the RCT in ICU mechanically ventilated septic shock patients; mortality did not differ (13% vs. 21%, n=201) (8). In 516 septic patients with cardiac dysfunction, no effect on mortality of levosimendan was observed (9). No studies used stratification.

*Traditional Chinese Medicine*

Chinese herbs are hypothesized to restore Yang and Qi to physical homeostasis (10). Traditional Chinese Medicine in sepsis is studied in 1 (8%) observational study and 12 (92%) RCTs (**Supplementary Table 9**). Most studied treatments included Xuebijing injection (n=4, 31%) and Shenfu injection (n=2, 15%).

*RCTs without a personalized approach –* A large recently published RCT showed that Xuebijing injections led to an absolute mortality reduction of 7% in 1817 sepsis patients admitted to the ICU (11). No studies investigating Traditional Chinese Medicine used stratification.

*Combinations of therapies*

Combining immunomodulatory agents from different treatment groups might provide beneficial results. This was examined in 34 studies (**Supplementary Table 14**), of which 16 (47%) were observation studies, 1 (3%) interventional non-RCT and 17 (50%) RCTs. Most studied combinations were corticosteroids and vasopressors (n=6, 19%), and ulinastatin and thymosin α1 (n=5, 16%).

*RCTs without a personalized approach –* In all identified RCT studying ulinastatin and thymosin α1, a positive effect on mortality was found (**Supplementary Table 14**), for example in 120 patients with sepsis due to carbapenem-resistant bacteria a 60-day mortality reduction was found (53% vs. 27%) (12).

*Studies with a personalized approach –* In a retrospective observational study including 100 patients with sepsis requiring norepinephrine (compared with 100 historical controls), a patient-tailored therapy protocol resulted in lower ICU-mortality (32 vs. 57%). The patient-tailored therapy protocol included the use of IgM-enriched immunoglobulins for patients with low plasma IgM levels, blood purification strategies for patients with high plasma levels of cytokines or endotoxin, albumin correction and modulation of vasoactive agents (13). In the PROVIDE IIa study, 240 sepsis patients were screened using ferritin and monocyte HLA-DR expression as biomarkers; treatment with either anakinra or interferon-γ was used to limited number of patients who were meeting the criteria of macrophage activation-like syndrome (MALS) or immunoparalysis. Findings of increased survival on day 7 (at the end of immunotherapy) (43% vs. 10%) did not last until day 28 (14).

*Other therapies*

Treatments that could not be classified into the previously mentioned treatment groups were studied in 2 (12%) observational studies and 15 (88%) RCTs (**Supplementary Table 15**). Most studied were alkaline phosphatase (n=4, 29%) and N-acetylcysteine (n=3, 21%).

*RCTs without a personalized approach –* Alkaline phosphatase did not improve short-term renal function, but did decrease the incidence of major adverse kidney events at day 90 and mortality (n=301) (15). Patient stratification was not studied in this treatment group.

**Supplementary Table 1. Data-extraction form**

|  |  | |
| --- | --- | --- |
| Study title |  | |
| First author |  | |
| Year of publication |  | |
| Country |  | |
| Study aim(s) |  | |
| Treatment group | - Innate immune response - Complement system - Coagulation and endothelial dysfunction - Pleiotropic drugs - Immunonutrition - Concomitant treatments - Non-pharmalogical treatments | - Traditional Chinese Medicine - Immunostimulatory cytokines and growth factors - Intravenous immunoglobulins - Mesenchymal stem cells - Immune-checkpoint inhibitors - Combinations of therapies - Other therapies |
| Study design | - Interventional, non-RCT - Interventional, RCT | - Observational |
| Sepsis definition used | - Sepsis, Sepsis-1 (1991) - Severe sepsis, Sepsis-1 (1991) - Septic shock, Sepsis-1 (1991) - Sepsis, Sepsis-2 (2001) - Severe sepsis, Sepsis-2 (2001) | - Septic shock, Sepsis-2 (2001) - Sepsis, Sepsis-3 (2016) - Septic shock, Sepsis-3 (2016) - Other, please describe: |
| Admission type | - Ward - Intensive Care Unit | - Emergency Department - Other, please describe: |
| Population type | - Hematology/neutropenic - Oncology - Elderly - Surgical | - Medical - Trauma - Other, please describe: |
| Number of patients studied |  |  |
| Intervention |  |  |
| Comparator (if any) |  |  |
| Primary endpoint |  |  |
| Analyses (crude and adjusted) |  |  |
| Were immune profiles/biomarkers measured? | Which one(s)? |  |
| Main findings |  |  |
| Was personalized medicine implemented? | How? |  |
| Limitations as stated by author |  |  |

**Supplementary Table 2. Innate immune response**

| **Treatment** | **Number of studies** | **Year** | **Total patients (median per study)** | **Setting** | **Personalized approach** | **If yes, specification** | **Treatment** | **Sepsis definition** | **Population** | **Clinical primary endpoint** | **Biochemi-cal primary endpoint** | **Main findings** |
| --- | --- | --- | --- | --- | --- | --- | --- | --- | --- | --- | --- | --- |
| **Observational studies** | | | | | | | | | | | | |
| **Anti-endotoxin**  (16-19) | 4 | 1993-1994 | 3354 (806) | ICU or not specified | No |  | HA-1A monoclonal antibody therapy | Sepsis-1 or not specified | General | Evaluation of criteria for administration of HA-1A, survival and hospital discharge, changes in hemodynamics, cost-effectiveness |  | Criteria for administration of HA-1A are not sensitive or specific; no difference in survival and hemodynamics; expensive treatment, not cost-saving |
| **IL-1 receptor antagonist**  (20-23) | 4 | 1996-2017 | 2256 (646) | ICU | Yes, two studies  (21, 22) | Re-analysis in patients with features of MAS; baseline IL-1 level | IL-1 receptor antagonist | Sepsis-1 or not specified | Medical or general | Interaction between IL-1 concentration and IL-1 ra treatment on mortality; mortality in patients with features of MALS, cardiac function; relationship between Predicted Risk of Mortality and efficacy of rhIL-1ra |  | Significant improvement in survival in patients with features of MALS, no significant interaction between IL-1 concentration and treatment, no effect on cardiac function; increase survival when using Predicted Risk of Mortality |
| **IL-6 receptor antagonist**  (24) | 1 | 2023 | 11643 (11643) | Not specified | No |  | Analysis single nucleotide polymorphisms (SNPs) in and near IL6R to evaluate the likely causal effects of IL6R blockade | Not specified | General | Mortality |  | Decreased mortality |
| **NSAID**  (25) | 1 | 2023 | 7694 (7694) | ICU | No |  | Aspirin | Sepsis-3 | General | Mortality |  | Decreaed mortality |
| **Ulinastatin**  (26) | 1 | 2021 | 182 (182) | ICU | No |  | Ulinastatin in combination with restrictive resuscitation fluid | Sepsis-3, shock | General | Extravascular lung water index, pulmonary vascular permeability index, systemic vascular resistance index, cardiac function, and renal function | Lactate, coagulation function | Lower lactate, inflammatory edema, vascular permeability and pulmonary edema; restored cardiac and renal functions |
| **Interventional studies** | | | | | | | | | | | | |
| ***Non-RCTs*** | | | | | | | | | | | | |
| **Anti-TNFα**  (27-30) | 4 | 1993-1995 | 129  (20) | ICU | Yes, one study  (30) | Subanalysis of patients with higher TNFα levels | Murine anti-TNFα antibody (MAK 195F, B-C7 MoAb, CB006) | Sepsis-1 | Medical and/or surgical | Hemodynamics, safety | Skeletal muscle pO_2_, cytokine levels and pharmacokinetics | Decrease in skeletal muscle pO_2_ might be indicator of sepsis improvement; higher dosage did not improve cytokine levels and hemodynamics; safe treatment; patients with higher TNFα levels might have a survival benefit |
| **Apoptotic cells**  (31) | 1 | 2021 | 10  (10) | ICU, ED | No |  | Allocetra™-OTS | Sepsis-3 | Medical | Safety |  | Safe treatment |
| ***RCTs*** | | | | | | | | | | | | |
| **Anti-endotoxin**  (32-41) | 10 | 1981-1995 | 4231  (145) | Ward, ICU or not specified | No |  | Human anti-LPS-specific globulin (LG-1), J5 antiserum (antiserum against endotoxin core), E5 (murine monoclonal antibody against endotoxin), HA-1A monoclonal antibody therapy, anti-LPS IgG | Sepsis-1 or not specified | Medical and/or (neuro)surgical or not specified, one trial ARDS patients, and one trial obstretic and gynaecogi-cal patients | Mortality, reversal of ARF, safety |  | No difference in ARF, may reduce early mortality, different results on mortality; safe treatment; lower TNF-levels |
| **Anti-TNFα**  (42-54) | 13 | 1995-2014 | 7744  (122) | Ward, ICU or not specified | Yes, two trials  (51, 52) | Patients with IL-6 levels> 1000 pg/mL | TNFα antibody (afelimomab/MAK 195F, AZD9773, BAY times 1351, cA2, CDP571, CytoFab) | Sepsis-1, sepsis-2, or not specified | Medical and/or surgical | Mortality, ventilator-free-days (VFDs); safety and tolerability | Pattern of cytokine activation, IL-6 and TNFα levels | Lower IL-6 and TNFα levels, no difference in mortality; different findings on mortality in patients with IL-6 >1000 pg/mL and VFDs; no difference in pattern of cytokine activation; safe and tolerable treatment |
| **Antitoxin liposomal agent**  (55) | 1 | 2019 | 19  (19) | ICU | No |  | Antitoxin liposomal agent (CAL02) | Not specified | CAP with S. pneumo-niae | Safety and tolerability |  | Safe and tolerable treatment |
| **Anti-CD14**  (56) | 1 | 2004 | 40  (40) | ICU | No |  | CD14 monoclonal antibody (IC14) | Sepsis-2 | Medical and/or surgical | Safety and tolerability |  | Safe and tolerable treatment |
| **Bradykinin Antagonist**  (57) | 1 | 1997 | 504 (504) | Not specified | No |  | Deltibant (CP-0127) | Not specified | General | Mortality |  | No effect |
| **Coenzyme Q10**  (58, 59) | 2 | 2015, 2020 | 78  (39) | ICU, ED | No |  | Coenzyme Q10 (CoQ10/ ubiquinone) | Sepsis-2, sepsis-3 | Medical or general |  | CoQ10 levels, , IL-6, TNFα, glutathione peroxidase and malondialdehyde (MDA) levels | Lower CoQ10, TNFα and MDA levels |
| **IL-11**  (60) | 1 | 2015 | 105  (105) | Ward, ICU or ED | Yes (60) | Thrombo-cytopenia | IL-11 therapy | Sepsis-2 | Patient with thrombo-cytopenia |  | Platelet recovery | Acceleration of platelet recovery |
| **IL-1 receptor antagonist**  (61-66) | 6 | 1994-1997 | 1770  (68) | Ward, ICU or not specified | No |  | IL-1 receptor antagonist | Sepsis-1 | Medical and/or surgical | Mortality; safety | Biomarker responses up to 72h | Reduction in activation markers up to 72-96h, no difference in mortality; safe treatment |
| **Mycobacterium w**  (67, 68) | 2 | 2015, 2021 | 252  (126) | ICU and Respiratory ICU | No |  | Mycobacterium w | Sepsis-1, sepsis-2 | Medical or general | Mortality |  | Different findings on mortality |
| **Nangibotide**  (69, 70) | 2 | 2020, 2023 | 404  (202) | ICU | Yes, one study (70) | sTREM-1 ≥ 400 pg/mL | Nangibotide | Sepsis-3, shock | Medical, surgical or general | AEs, death, organ function, SOFA score | Pharmaco-dynamics | No difference in TEAEs, different results on decrease in SOFA |
| **NSAID**  (71-75) | 5 | 1991-2004 | 1009  (40) | ICU | Yes, one study (71) | Hypothermic patients | Ibuprofen, lornoxicam | Sepsis-1 or not specified | Medical and/or surgical (in one trial only hypother-mic septic patients) or general | Duration of ventilation, length of ICU stay, 30-day mortality and organ failure; pathophysiologic features, safety and physiologic actions | Metabo-lites and cytokine levels | Different results on in mortality, no reduction in organ failure, duration of ventilation or length of ICU stay; reduction in arachidonic acid metabolism and associated pathophysiological features; safe treatment, poor rectal drug absorption |
| **p55 TNF receptor fusion protein**  (76-78) | 3 | 1997-2001 | 2005  (498) | Ward, ICU or not specified | No |  | p55 TNF receptor fusion protein (p55-IgG/lenercept) | Sepsis-1 or not specified | Medical | Mortality |  | No difference |
| **Phospholipid emulsion**  (79) | 1 | 2009 | 1379  (1379) | ICU | No |  | Phospholipid emulsion | Sepsis-2 | General | Mortality |  | No difference |
| **Pirfenidone**  (80) | 1 | 2021 | 176  (176) | Not specified | No |  | Pirfenidone | Sepsis-3 | Medical |  | Creatinine and urinary volume | No difference |
| **Taurolidine**  (81) | 1 | 1995 | 100  (100) | ICU | No |  | Taurolidine | Not specified | Medical | Clinical or bacteriologic outcome | Endotoxin activity | No difference |
| **TLR4a**  (82-85) | 4 | 2010-2013 | 2827  (296) | ICU | No |  | Eritoran, TAK-242 | Sepsis-1 or not specified | Medical | Mortality | IL-6 levels | No difference |
| **TNFR:Fc**  (86) | 1 | 1995 | 141  (141) | ICU | No |  | Extracellular portion of TNF receptor + Fc portion of IgG1 (TNFR:Fc) | Sepsis-1, shock | Medical | Mortality |  | No difference |
| **Ulinastin**  (87, 88) | 2 | 2020, 2023 | 128  (64) | ICU | No |  | Ulinastin | Sepsis-1 or sepsis-3, shock | Medical | Mortality | Inflammatory markers | Decrease in inflammatory markers and mortality |

Abbreviations: ARDS, acute respiratory stress syndrome; ARF, acute respiratory failure; CAP, community acquired pneumonia; ED, emergency department; ICU, intensive care unit; IgG, immunoglobulin G; IL, interleukin; LPS, lipopolysaccharides; MALS, macrophage acting-like syndrome; NSAID, non-steroidal anti-inflammatory drugs; RCT, randomized controlled trial; SOFA, Sequential Organ Failure Assessment; TLR, toll-like receptor; TNF(r), tumor necrosis factor (receptor); TREM-1, triggering receptor expressed on myeloid cells-1 .

| **Supplementary Table 3. Non-pharmacological treatments** | | | | | | | | | | |  | |  |  |  |
| --- | --- | --- | --- | --- | --- | --- | --- | --- | --- | --- | --- | --- | --- | --- | --- |
| **Treatment** | **Number of studies** | **Year** | **Total patients (median per study)** | | **Setting** | **Perso-nalized ap-proach** | **If yes, specifi-cation** | **Treatment** | **Sepsis definition** | **Population** | | **Clinical primary endpoint** | | **Bioche-mical primary endpoint** | **Main findings** |
| **Observational studies** | | | | | | | | | | | | | | | |
| **Blood purification**  (89-138) | 50 | 1998 – 2023 | 61825 (45) | | ICU, Ward, ED, or not specified | Yes, four studies  (104, 107, 137, 138) | Patients with EAA 0.6 - 0.89; IL-6 > 1000 ng/l | CytoSorb, CytoSorb + ECMO, endotoxin and cytokine adsorption, (polymyxin B) hemoperfusion | Sepsis-1, shock sepsis-2, shock, sepsis-3, shock or not specified | Medical, surgical or general | | Mortality, hemodynamics, SOFA score,  inotropic scores, multiple organ failure, recovery from cardiomyopathy, subgroups likely to benefit | | Inflammatory markers, Neutrophil ROS, endotoxins, inflammatory markers | Different findings on mortality, significant reduction in SOFA score, lower EAA. Blood pressure increased post-treatment in a number of studies. Different findings on inflammatory markers, neutrophil ROS was not affected by hemoperfusion, effect on recovery from cardiomyopathy. Blood endotoxin level decreased. Subgroups with PT-INR > 1.4 or lactate > 3 mmol/L benefit most |
| **Filtration**  (139-149) | 11 | 2001 – 2023 | 1468 (35) | | ICU, ED or not specified | Yes, one study  (147) | Hyperthermic patiens | Continuous renal replacement, hemodialysis or  hemofiltration | Sepsis-1, shock, sepsis-3, shock or not specified | Medical, surgical or general | | Mortality, mechanical ventilation, vasopressor requirements, MAP, resuscitation time, SOFA score | | Cytokine levels | Significantly lower mortality rate. Fewer days on vasopressors and mechanical ventilation. Lower levels of inflammatory cytokines. |
| **Plasma treatments**  (150, 151) | 2 | 2013, 2015 | 45 (23) | | ICU or not specified | No |  | Coupled plasma filtration adsorption | Sepsis-2, shock or not specified | Surgical or general | | Safety | |  | Safe treatment. Increased MAP, decreased EAA and PCT. No difference in survival rate |
| **Interventional studies** | | | | | | | | | | | | | | | |
| ***Non-RCTs*** | | | | | | | | | | | | | | | |
| **Blood purification**  (152-185) | 34 | 1997 - 2021 | 1080  (21) | | ICU or not specified | Yes, two studies  (152, 175) | IL-6 > 1000 ng/l and a vasopressor dependency index > 3; EAA level > 0.6 | Cytokine adsorption, endotoxin absorption or polymyxin B hemoperfusion | Sepsis-1, shock, sepsis-2, shock or not specified | Medical, surgical or general | | Survival and mortality data, hemodynamic and respiratory parameters, vasopressor requirements | | Cytokine levels and other indices of inflammation | Different finding on mortality, reduction in vasopressor requirements, improved hemodynamics and lactate, decreased cytokine levels |
| **Filtration**  (186-196) | 11 | 1993 - 2014 | 233  (16) | | ICU | No |  | Hemo(dia)filtration | Sepsis-1, shock or not specified | Medical, surgical or general | | Hemodynamics | | Cytokine levels | Results varied; the majority of studies showed significant toxin elimination. Improved hemodynamics, decreased cytokines and lactate level |
| **Plasma treatments**  (197-200) | 4 | 2002 - 2018 | 52  (13) | | ICU | No |  | Coupled plasma filtration adsorption, extracorporeal plasma treatment or plasma exchange | Sepsis-1, shock, sepsis-3, shock or not specified | Medical or surgical | | MAP, vasopressor requirements and adverse events | | Cytokine levels, changes in plasma levels of LPS | Increased antiinflammatory and decreased proinflammatory cytokines; increased MAP and decreased vasopressors, not mortality difference, no advserve events |
| **Leukaplasmapharesis**  (201) | 1 | 1992 | 13  (13) | | ICU | No |  | Leukaplasmapharesis | Not specified | Medical | | Mortality | |  | Decreased mortality |
| **LPS adsorption and haemodialysis**  (202) | 1 | 2014 | 26  (26) | | ICU | No |  | LPS adsorption and haemodialysis | Not specified | Surgical | | Effectiveness and safety | | PCT | survival, increased MAP, decreased PCT |
| ***RCTs*** | | | | | | | | | | | | | | | |
| **Blood purification**  (203-229) | 27 | 1999 - 2023 | 2553 (64) | ICU or not specified | | Yes, three studies  (219, 224, 228) | In patients with blood EAA level > 0.6, hypo-thermic patients | Blood purification, cytokine adsorption, endotoxin absorption, hemoperfusion, LPS adsorber therapy, polymyxin B hemoperfusion | Sepsis-1, shock, sepsis-2, shock, sepsis-3, shock or not specified | Medical, surgical or general | | Mortality, safety, APACHE and SOFA score, length of ICU stay, lung injury measurements, hemodynamics, adverse events, resolution of shock | | Cytokine and endotoxin levels | Different findings on inflammatory cytokine levels, no difference in SOPA and APACHE, decreased vasopressor requirements, blood pressure increased post-treatment, shorter ICU stays, lower lung injury scores, different findings on mortality, no difference in adverse events. |
| **Filtration**  (230-254) | 25 | 1995 - 2023 | 1482 (30) | ICU or not specified | | No |  | Continuous renal replacement, hemodialysis or hemo(dia)filtration | Sepsis-1, shock, sepsis-2, shock, sepsis-3, shock or not specified | Medical, surgica or general | | Mortality, ICU/hospital stay length, hemodynamics | | Inflammatory cytokines, iron levels, renal function indices, endotoxin levels | Improved renal function parameters, lower endotoxin levels, different findings on inflammatory cytokine levels, lower vasopressor requirements, and shorter ICU stay time, different findings on mortality |
| **Treatment** | **Number of studies** | **Year** | **Total patients (median per study)** | | **Setting** | **Perso-nalized ap-proach** | **If yes, specifi-cation** | **Treatment** | **Sepsis definition** | **Population** | | **Clinical primary endpoint** | | **Bioche-mical primary endpoint** | **Main findings** |
| **Plasma treatments**  (255-260) | 6 | 2012 - 2022 | 330  (32) | ICU | | No |  | Coupled plasma filtration adsorption or plasma exchange | Sepsis-1, shock, sepsis-2, shock, sepsis-3, shock or not specified | Medical, surgical or not specified | | Mortality, hemodyanmics | | Cytokine levels | Improved hemodynamics, lower levels of IL-6, no difference in mortality rates |
| **Hemoperfusion + hemofiltration**  (261) | 1 | 2020 | 30  (30) | ICU | | No |  | Hemoperfusion + hemofiltration | Sepsis-3, shock | Medical | | None | | Cytokine levels | Decreased inflammatory markers |

Abbreviations: EAA, endotoxin activity assay; APACHE, Acute Physiology And Chronic Health Evaluation; ECMO, extracorporeal membrane oxygenation; ED, emergency department; HR, heart rate; ICU, intensive care unit; IL, interleukin; LPS, lipopolysaccharides; MAP, mean arterial pressure; PCT, procalcitonin; RCT, randomized controlled trial; ROS, reactive oxygen species; SOFA, sequential organ failure assessment.

**Supplementary Table 4. Complement system**

| **Treatment** | **Number of studies** | **Year** | **Total patients (median per study)** | **Setting** | **Personalized approach** | **If yes, specification** | **Treatment** | **Sepsis definition** | **Population** | **Clinical primary endpoint** | **Biochemical primary endpoint** | **Main findings** |
| --- | --- | --- | --- | --- | --- | --- | --- | --- | --- | --- | --- | --- |
| **Interventional studies** | | | | | | | | | | | | |
| ***RCTs*** | | | | | | | | | | | | |
| **Complement inhibition**  (262-265) | 4 | 2002-2021 | 213  (51) | ICU | No |  | C1-inhibitor, C1-esterase inhibitor, vilobelimab | Sepsis-1, sepsis-3, shock | Medical, surgical | Mortality pharmaco-dynamics, pharmacokinetics and safety | C1-inhibitor antigen and activity levels, EA complex and lactoferrin levels levels | Increased plasma C1-inhibitor antigen and activity, neutralizes C5a in a dose-dependent manner, higher C3 levels and decreased CRP; reduced EA levels; safe, reduced all-cause mortality as well as sepsis-related mortality |

Abbreviations: EA, elastase- α1-antitrypsin; C, complement; CRP, C-reactive protein; ICU, intensive care unit; RCT, randomized controlled trial.

**Supplementary Table 5. Coagulation and endothelial dysfunction**

| **Treatment** | **Number of studies** | **Year** | **Total patients (median per study)** | **Setting** | **Personali-zed approach** | **If yes, specifica-tion** | **Treatment** | **Sepsis definition** | **Population** | **Clinical primary endpoint** | **Biochemical primary endpoint** | **Main findings** |
| --- | --- | --- | --- | --- | --- | --- | --- | --- | --- | --- | --- | --- |
| **Observational studies** | | | | | | | | | | | | |
| **Activated protein C/drotrecogin alfa**  (266-321) | 56 | 2000  -  2023 | 127724 (328) | Ward, ED, ICU, critical care unit, or not specified | Yes, three articles  (288, 320, 321) | Based on troponin levels, on clinical and genetic marker-defined  patient subgroups and on previously identified ARDS subphenotypes | Protein C concentrate, Drotrecogin alfa (activated), DrotAA, rhAPC, APC | Sepsis-1, shock, sepsis-2, shock, or not specified | Surgical, medical or not specified | Mortality, length-of-stay, costs, HRQoL, SAEs, filter changes, wastage of agent, length till discharge of ICU, clinical characteristics, MAP, heartrate, CVP and norepinephrine dose | Biomarkers, kidney function, S100B, neopterin levels, fibrinolytic biomarkers | Reversal of peripheral ischemia, improvement of DIC, reduction of mortality, higher cost effectiveness ratio, less serious bleeding events, modest cost/QALY, shorter stay in hospital, better physical component score long term, no effect on kidney function, lower CRP, IL-6 and PAI-1, lower norepinephrine use and quicker improvement of MAP, decrease of S100B, no effect on long-term mortality. Better survival in some marker-defined patient subgroups and in the hyperinflammatory subphenotype. |
| **Antithrombin therapy**  (322-336) | 15 | 2002  -  2023 | 18871 (648) | ICU, ED, critical care department, or not specified | Yes, two articles  (334, 335) | Based on AT activity | Antithrombin III, antithrombin, AT1500 vs AT3000 | Sepsis-1, shock, sepsis-2, shock, or not specified | Surgical, medical or not specified | Mortality, outcome, red cell transfusion rate, bleeding events, PICS | Antithrombin activit | Decreased mortality, increase in bleeding incidence, better results in septic patients with DIC than without, better results in patients with very low AT activity and improved AT after treatment. No difference in occurrence of PICS |
| **Antithrombin therapy + Thrombomodulin**  (337-345) | 9 | 2016  -  2023 | 12622  (1198) | Ward, ICU, or not specified | Yes, two articles (344, 345) | High PT-INR, four groups  depending on antithrombin and fibrinogen levels | Antithrombin + recombinant thrombomodulin, antithrombin + TM-alpha | Sepsis-1, shock, sepsis-2, shock, or not specified | Surgical, medical or not specified | SOFA, DIC score, mortality, outcomes | Coagulation and inflammation markers | Different findings on mortality. Improvement of platelets and D-dimer. No increase in risk of bleeding. No decrease of SOFA, more red cell transfusion. Most benefit on survival when 1) AT < 50% and fibrinogen < 1.5 g/L or 2) PT-INR ≥ 1.5 |
| **Endothelin**  (346) | 1 | 2000 | 23  (23) | ICU | No |  | Afelimomab | Sepsis-1, shock | Medical | Organ function | Proinflammatory cytokines | Decrease in big-endothelin |
| **Heparin**  (347-349) | 3 | 2008  -  2023 | 7523  (2326) | ICU | No |  | Heparin, UFH | Sepsis-1, shock; sepsis-3 | Medical, sepsis-induced coagulopathy | Mortality |  | Decreased mortality, more successful liberation of mechanical ventilation and vasopressors. No difference in major hemorrhage or transfusion. |
| **Pentoxifylline**  (350) | 1 | 1996 | 24  (24) | ICU | No |  | Pentoxifylline | Sepsis-1 septic shock | Medical |  | TNF-R and IL-6 | Decrease in TNF and cardiac index, increase in IL-6 and systemic vascular resistance |
| **Several anticoagu-lants**  (351-354) | 4 | 2016  -  2019 | 9933  (2780) | ICU | No |  | Any anticoagulation | Sepsis-2, shock or not specified | Surgical, medical | Mortality |  | Decreased mortality for DIC patients and high risk patients. |
| **Thrombo-modulin**  (355-366) | 12 | 2013  -  2023 | 15315  (542) | ICU or not specified | Yes, two studies (360, 361) | Based on baseline coagulation biomarker levels, based on kidney function; platelet count between 30 × 10/L and 150 × 10/L or greater than 30% decrease within 24 hours | RhTM, ART-123, TM-alfa | Sepsis-2, shock sepsis-3, or not specified | Neutropenic, surgical, medical, pediatric, DIC | DIC score, mortality, ventilator free days, side effects | DIC resolution | Decrease in mortality and D-dimer in DIC patients, increase of prothrombin and thrombin-antithrombin complex. Reduction of mortality in subgroup of high risk patients. No reduction of mortality overall. Recommended plasma concentration of TM alfa for treatment of septic DIC is 1,010 ng/mL. |
| **Thrombo-poetin**  (367, 368) | 2 | 2014, 2020 | 285 (143) | Ward, ICU | Yes (367, 368) | Thrombocytopenia | rhTPO | Sepsis-2 severe sepsis; sepsis-3 sepsis | Surgical, thrombocytopenia |  | Platelets | Greater increase platelets, shorter ICU treatment, no difference mortality |
| **Interventional studies** | | | | | | | | | | | | |
| ***Non-RCTs*** | | | | | | | | | | | | |
| **Activated protein C/drotre-cogin alfa**  (369-375) | 7 | 2004  -  2018 | 4310  (40) | ICU | No |  | Drotecogin activated, DrotAA, APC, PC concentrates | Sepsis-2 or not specified | Oncology, medical or not specified | Mortality, safety and efficacy, microcirtculatory alterations, ptCO2 | Improvement of coagulation and inflammatory markers | Lower mortality, improved survival rate and decreased thrombosis, improvement of sepsis-induced microvascular alterations. Increased serious bleeding. |
| **Thrombo-modulin**  (376, 377) | 2 | 2011 | 151  (76) | ICU | No |  | RhTM | Sepsis-2 or not specified | Medical | DIC resolution rate, SOFA and lung injury score |  | Lower mortality, decreased SOFA |
| ***RCTs*** | | | | | | | | | | | | |
| **ACE inhibitor**  (378) | 1 | 1998 | 40  (40) | ICU | No |  | Enalaprilat | Sepsis-1, shock | Surgical |  | endothelin-1, angiotensin II, soluble thrombomodulin, and soluble adhesion molecules (endothelial leukocyte adhesion molecule-1, intercellular adhesion molecule-1, vascular cell adhesion molecule-1, and granule membrane protein-140) | Decrease in soluble thrombomodulin and soluble adhesion molecules, less severe sepsis and septic shock. No difference in survival rate. |
| **Activated protein C/drotre-cogin alfa**  (379-392) | 14 | 2001  -  2016 | 19319  (1960) | Ward, ICU, or not specified | Yes, two articles  (390, 391) | Based on severity of protein C deficiency | rhAPC, Drotecogin alfa activated, DrotAA, heparin + DrotAA | Sepsis-1, shock, sepsis-2, shock or not specified | Surgical, medical, or not specified | SAEs, mortality, costs, survival, time to resolution of vasopressor-dependent hypotension | APC antibody response, pharmacokinetics, APTT, D-dimer, protein C, IL-6 | Different findings on serious bleeding events and reduction of mortality. No difference in SAEs, decrease in SOFA, cost effective in APACHE II >25, increase in survival. Higher length of stay. |
| **Adrenome-dullin Ab**  (393) | 1 | 2021 | 301  (301) | ICU | Yes  (393) | Adrenomedullin > 70pg/mL | Adrecizumab | Not specified | Surgical, medical | SAEs, TEAEs, mortality, blood pressure, heart rate, episodes of interruption of infusion |  | No difference in TEAEs or mortality, well tolerated |
| **Antithrom-bin therapy**  (394-403) | 10 | 1993  -  2013 | 5027  (41) | ICU or not specified | No |  | ATIII, ATIII concentrate, Kybernin P, antithrombin substitution | Sepsis-1, shock or not specified | Surgical, medical, or not specified | Mortality, long term effect, survival, Karnofsky scale and visual analog scales in 6 domains, recovery from DIC | Coagulatory variables, TEG, plasmatic coagulation tests, plasma levels of fibrinogen, and platelet counts | Reduction of DIC duration, correction of ATIII levels, trend towards survival, increased PF ratio, decrease in pulmonary hypertension index, decrease in bilirubin and less RRT, well tolerated, decrease in mortality, decrease SOFA, decrease of IL-6, sICAM-1, sE-selection and CRP, improved QoL, increase in platelets. No effect on IL-8, PMN elastase or leukocytes or SOFA |
| **Heparin + antithrom-bin therapy**  (404) | 1 | 2006 | 2314  (2314) | ICU | No |  | Heparin + antithrombin | Not specified | Surgical, medical | Mortality |  | Increased risk of bleeding, no difference in 28-day mortality |
| **Heparin**  (405-410) | 6 | 2013  -  2023 | 2699  (160) | ICU, ED | No |  | Heparin, UFH | Sepsis-1, sepsis-2, shock, sepsis-3 or not specified | Surgical, medical | LOS-MOD score, active bleeding rate | Plasma F1+2, ATIII, PAI-1, heparanase and syndecan-1 | Safe, decrease in prothrombin fragement, thrombin-antithrombin complex, heparanase and syndecan-1, increase in ventilator free days, shorter stay ICU, conflicting results on bleeding, decrease in D-dimer. No effect on mortality or SOFA. |
| **Iloprost + eptifiba-tide**  (411) | 1 | 2019 | 24  (24) | ICU | No |  | Prostacyclin or eptifibatide | Sepsis-2, shock | Surgical, medical |  | Change in biomarkers, change in platelet count at 48 h, change in d-dimer and firing split products indicative of fibrinolysis | Decrease in SOFA, decrease of sE-selectin, fibrin monomers and sVEGFR1, no increase of thrombomodulin and nucleasomes. |
| **Pentoxifyl-line**  (412-419) | 8 | 1995  -  2020 | 282  (35) | ICU | No |  | Pentoxifylline | Sepsis-1, shock, sepsis-3, shock | Surgical, medical or not specified | Survival rate, VO2 differences, SOFA | TNF, IL-6, IL-8, IL-10, platelet aggregation, sELAM-1, sICAM-1, soluble granule membrane protein-140, thrombomodulin, protein C, (free) protein S, and thrombin-antithrombin plasma concentrations | Decrease in TNF, decrease IL-10, less impaired platelet aggregation, increase in protein C concentration, decrease in SOFA, improvement of PF ratio improvement pressure adjusted heart rate. No difference in IL-6, IL-8, endotoxin and TNF- α. |
| **Platelet-activating factor receptor antagonist**  (420-423) | 4 | 2000  -  2004 | 469  (115) | ICU or not specified | No |  | BB - 882, Lexipafant, BN 52021, TCV-309 | Sepsis-1, sepsis-3, shock or not specified | Surgical, medical | Mortality, MOF score, recovery from shock, safety |  | No effect on mortality or cytokines. More recovery of shock. |
| **Thrombo-modulin**  (424-428) | 5 | 2013  -  2019 | 3505  (750) | ICU or not specified) | No |  | ART-123, trombome-dulin, rhTM, rhsTM | Sepsis-1, shock, sepsis-2, shock or not specified | Medical or not specified | Mortality, survival, effect and safety, DIC resolution |  | No effect on mortality, organ function, inflammatory markers, bleeding or thrombosis or new infections. More recovery from DIC, lower D-dimer. |
| **Tissue factor antagonist**  (429-432) | 4 | 2001  -  2012 | 4120  (982) | ICU, or not specified | No |  | ALT-836, Tifacogin, recombinant tissue factor pathway inhibitor | Sepsis-2 or not specified | Medical | Mortality, safety | Pharmacokinetics | Conflicting effects on mortality, different findings on risk of bleeding. |

Abbreviations: Ab, antibody; ACE, angiotensin-converting-enzyme; APACHE, Acute Physiology And Chronic Health Evaluation; (A)PC, (activated) protein C; aptt, activated partial thromboplastin time; AT, antithrombin; CVP, central venous pressure; CRP, C-reactive protein; DIC, disseminated intravascular coagulation; DrotAA, drotrecogin alfa; ED, emergency department; HRQoL, health-related quality of life; ICU, intensive care unit; IL, interleukin; LOS-MOD, length of stay-multiple organ dysfunction; MAP, mean arterial pressure; MOF, multiple organ failure; PAI-1, plasminogen activator inhibitor-1; PF ratio, pO2/FiO2 ratio; PICS, persistent inflammation, immunosuppression, and catabolism syndrome; PMN, polymorphonuclear; ptCO2, transcutaneous carbon dioxide; RCT, randomized controlled trial; (rh)APC, (recombinant human) activated protein C; (rh)(s)TM, (recombinant human) (soluble) thrombomodulin; (rh)TPO, (recombinant human) thrombopoetin; RRT, renal replacement therapy; SAE, severe adverse events; sELAM-1, soluble endothelial leucocyte adhesion molecule-1; sE-selectin; soluble E-selectin; sICAM-1, soluble intercellular adhesion molecule-1; SOFA, sequential organ failure assessment; sVEGFR1, soluble vascular endothelial growth factor receptor 1; TEAE, treatment emergent adverse events; TEG, thromboelastography; TNF(-R), tumor necrosis factor(-receptor); UFH, unfractioned heparin.

**Supplementary Table 6. Pleiotropic drugs**

| **Treatment** | **Number of studies** | **Year** | **Total patients (median per study)** | **Setting** | **Persona-lized approach** | **If yes, specifi-cation** | **Treatment** | **Sepsis definition** | **Population** | **Clinical primary endpoint** | **Biochemi-cal primary endpoint** | **Main findings** |
| --- | --- | --- | --- | --- | --- | --- | --- | --- | --- | --- | --- | --- |
| **Observational studies** | | | | | | | | | | | | |
| **Antibiotics**  (433-437) | 5 | 2003  -  2021 | 1845  (237) | Ward, ICU, ED | No |  | Macrolides (azithromycin), cephalosporins, vancomycin + piperacillin-tazobactam, etimicin sulfate combined with cefotaxime vs cefotaxime alone | Sepsis-1, sepsis-2, shock or not specified | Medical or surgical | Treatment effect, need for RRT, mechanical ventilation time, length of hospital stay, mortality | Inflamma-tory levels, levels of kidney function biomarkers | Azithromycin led to less adverse kidney events; cephalosporins led to an increase release of TNF-α; macrolides led to a lower mortality; vancomycin + piperacillin-tazobactam led to AKI, no difference in mortality; etimicin sulfate combined with cefotaxime led to lower inflammatory markers and better treatment effect |
| **Corticosteroids - low dose**  (438-470) | 33 | 1999  -  2024 | 145421  (219) | ICU, MCU and/or ward | Yes, nine studies  (438, 444, 446, 449-451, 458, 466, 470) | Adrenal insufficiency (tested by corticotropin test), CRP >150 mg/L, allocated to either transcript-mic signatures SRS1 or SRS2, expression of adrenocorti-cal candidate genes or the estimated individual treatment effect as derived from machine learning, low serum IFNγ/IL10 ratio, transciptomic data, actor-critic RL algorithm, prediction model with logistic regression | Corticosteroids - low dose (<400mg/day hydrocortisone or equivalent) vs placebo or standard care, one study early vs late treatment, two studies hydrocortisone vs methylpred-nisone, one study fludrocortisone and hydrocortisone vs hydrocortisone, abrupt versus taper discontinuation of corticosteroids | Sepsis-1, shock, sepsis-2, shock, sepsis-3, shock or not specified | Medical and/or surgical or not specified | Vasopressor use, hemodyna-mics, SOFA score, shock reversal, vasopressor dependency index and cardiac power index, mortality, length of hospital stya, PTSD, safety, effectiveness, QoL, prediction of cortico-sensitivity or resistance | Inflamma-tory levels | Different findings on mortality and time to shock reversal, methylprednisone and hydrocortisone had similar effects, transcriptomic profile at onset of septic shock was associated with response to corticosteroids, higher length of hospital stay. Positive effects of the estimation of the individual treatment effect with machine based learning and other prediction models, low serum IFNγ/IL10 ratio predicted increased survival, no association between adrenocortical candidate gene expression and mortality, improved vasopressor use with early treatment vs late, reduced incidence of PTSD, decrease in IL-12 |
| **Corticosteroids - high dose**  (471) | 1 | 2015 | 2164  (2164) | Not speci-fied | No |  | Corticosteroids - high dose (>400mg/day hydrocortisone or equivalent) | Not specified | Surgical | Mortality |  | Decrease in mortality |
| **Corticosteroids - late vs. high dose**  (472) | 1 | 2021 | 319  (319) | ICU | No |  | Corticosteroids - low vs dose (low = <400mg/day or high = >400mg/day hydrocortisone or equivalent) | Not specified | Medical | Shock reversal |  | No difference |
| **Corticosteroids - not specified***  (473-481) | 9 | 2014  -  2023 | 18186  (351) | ICU or not speci-fied | Yes, three studies  (474, 478, 480) | CRP > 15 mg/dL, neutropenic patients or genes to generate continuous scores to define previously established subclasses of sepsis | Corticosteroids* | Sepsis-1, sepsis-3, shock or not specified, shock | Medical, neutropenia or not specified | Mortality | Lympho-cyte count | Different findings on mortality, response to corticosteroids may differ according to lymphocyte count, one of the gene expression scores may be harmed by hydrocortisone |
| **Corticosteroids + Vitamin C**  (482) | 1 | 2020 | 144  (144) | ICU | No |  | Vitamin C and hydrocortisone | Not specified, shock | Not specified | Mortality |  | No difference |
| **Corticosteroids + Vitamin C + Thiamine**  (483-490) | 8 | 2017  -  2023 | 817  (99) | ICU | Yes, one study (490) | Baseline serum active renin levels | Vitamin C, hydrocortisone, and thiamine | Sepsis-1, shock, sepsis-3, shock or not specified, shock | Medical and/or surgical or not specified | Improvement of refractory shock, HR-QoL, mortality |  | Lower mechanical ventilation and administration of vessopressors duration, no difference in HR-QoL, different findings on mortality. Baseline serum active renin levels are associated with mortality |
| **Statin**  (491) | 1 | 2022 | 511  (511) | ICU | Yes  (491) | High baseline plasma IL-18 (≥ 800 pg/ml) | Simvastatin | Not specified | Not specified | Mortality |  | Lower mortality in patients with high baseline plasma IL-18 |
| **Thiamine**  (492) | 1 | 2020 | 68571  (68571) | Ward, ICU, ED | No |  | Thiamine | Sepsis-3, shock | Medical | Mortality |  | No difference |
| **Vitamin C**  (493, 494) | 2 | 2022,  2023 | 73480  (36740) | ICU | No |  | Vitamin C | Sepsis-3, shock or not specified | Medical | Mortality |  | Decrease in mortality, abrupt termination may increase mortality |
| **Vitamin C + Thiamine**  (495, 496) | 2 | 2022, 2023 | 217  (109) | ICU, ED | No |  | Vitamin C and thiamine | Sepsis-3, shock | Medical or not specified | Mortality | Inflamma-tory levels | No difference in inflammatory levels, different findings on mortality |
| **Interventional studies** | | | | | | | | | | | | |
| ***Non-RCTs*** | | | | | | | | | | | | |
| **Antibiotics**  (497) | 1 | 2023 | 70  (70) | Not specified | No |  | Silver sulfadiazine | Not specified | Patients with burns | Mortality | Inflamma-tory levels | Decreased inflammatory reactions,  and lower mortality |
| **Corticosteroids - low dose**  (498-500) | 3 | 1994 - 2018 | 182  (57) | ICU or ED | No |  | Corticosteroids - low dose (<400mg/day hydrocortisone or equivalent) | Sepsis-1, sepsis-2, shock or not specified | Medical and/or surgical | Hemodyna-mics, sublingual microcircu-lation | Inflamma-tory levels | Arteriolar vasoconstriction, anti-inflammatory effects and hemodynamic reversal |
| **Corticosteroids - high dose**  (501) | 1 | 1984 | 20  (20) | Not speci-fied | No |  | Bethametha-sone | Not specified, shock | Medical and/or surgical | Shock reversal |  | Reversal of shock was achieved in all patients |
| **Vitamin C**  (502) | 1 | 2022 | 30  (30) | ICU | No |  | Vitamin C | Sepsis-3, shock | Not specified | Skin microvascular reactivity |  | Increase in skin microvascular reactivity |
| ***RCTs*** | | | | | | | | | | | | |
| **Aminophylline**  (503) | 1 | 2022 | 100  (100) | ICU | No |  | Aminophylline | Sepsis-3 | Medical | Mortality |  | Decrease in mortality |
| **Antibiotics**  (504-515) | 12 | 1995 - 2022 | 1518  (57) | Ward, ICU or not speci-fied | No |  | Imipenem vs ceftazidim, imipenem vs meropenem, cefuroxim vvs ciprofloxacin, ceftazidim vs cirpofloxacin, clarithromycin vs placeo, doxycycline vs placebo | Sepsis-1, shock, sepsis-2, shock, sepsis-3, shock or not specified | Medical and/or surgical or general | Mortality, signs and reversal of systemic inflammation/infection, duration of mechanical ventilation | Inflamma-tory and endotoxin levels | Different findings on levels of endotoxin when comparing imipenem and ceftazidim, no differences in cytokine levels. Imipenem or meropenem did not affect systemic endotoxemia. Clarithromycin accelerated the resolution of infection and weaning of mechanical ventilation, increase expression of TNF-α, IL-6 and sTREM-1 by monocytes, lower TNF-α and IL-6 levels, different findings on mortality. Doxycline did not affect MMP-8, -9 or TIMP-1 concentrations. Cefuroxime and ciprofloxacin did not result in different LPS and TNF-α levels. |
| **Corticosteroids - low dose**  (516-541) | 26 | 2002 - 2023 | 11011  (119) | Ward, ICU | Yes, two trials  (538, 540) | Adrenocorticotropic hormone (ACTH)-cortisol response (delta <13 µg/dL), CRP > 150 | Corticosteroids - low dose (<400mg/day hydrocortisone or equivalent) vs placebo or standard care, one study early vs late treatment, one study dexamethasone vs methylpredni-sone | Sepsis-1, shock, sepsis-2, shock, sepsis-3, shock or not specified, shock | Medical and/or surgical or general | Renal function, vasopressor use, shock reversal, development of septic shock, mortality, treatment failure, QoL | Inflamma-tory levels, microal-buminuria to creatinine ratio, glucose levels | Different results on mortality and shock reversal, no effect on QoL, not reduction in the risk of septic shock, lower IL-6, apoptosis rate of CD4+ cell, microalbuminuria to creatinine ratio, CRP and lower SOFA score, improvements in hemodynamics and renal function, 100mg methylprednisone led to less hyperglycemia than 200mg, intermittent bolus led to higher shock reversal than continuous bolus and no difference in hyperglycemia, no effect of early initiation |
| **Corticosteroids - high dose**  (542-550) | 9 | 1970 - 2005 | 1210  (50) | ICU or not speci-fied | No |  | Corticosteroids - high dose (>400mg/day hydrocortisone or equivalent) | Sepsis-1, shock, sepsis-2, shock or not specified | Medical and/or surgical | Shock reversal, development of septic shock or ARDS, mortality, PTSD | Inflamma-tory levels, P/F ratio | No effect on shock reversal or ARDS, different findings on mortality, reduced incidence of PTSD, lower P/F ratio, reduction in IL-8 serum, but not in S-100B serum |
| **Corticosteroids - late vs. high dose**  (551) | 1 | 2016 | 122  (122) | ICU | No |  | High (100 mg bolus + 300mg per day) hydrocortisone vs low (200mg per day) | Sepsis-2, shock | Medical and/or surgical | Mortality |  | No difference |
| **Corticosteroids - not specified***  (552-556) | 5 | 1987 - 2007 | 345 (29) | ICU or not speci-fied | No |  | Corticosteroids* | Sepsis-1, shock or not specified | Medical or general | Vasopressor use, shock reversal, mortality | Inflamma-tory levels, P/F ratio | No effect on mortality, reduction in time to cessation of vasopressor therapy, lower IL-6 and IL-8, no effect on TNF or IL-10 |
| **Corticosteroids + Vitamin C + Thiamine**  (557-571) | 15 | 2018 - 2023 | 2411 (106) | ICU | No |  | Vitamin C, hydrocortisone, and thiamine vs hydrocortisone or placebo | Sepsis-2, shock, sepsis-3, shock or not specified, shock | Medical and/or surgical or general | Perfusion vascular density, SOFA score,ventila-tor- and vasopressor-free day, shock reversal and mortality. Long-term cognitive, psychological, and functional outcomes | Inflamma-tory levels, lactate | Different findings on SOFA score, shock reversal, ventilator-free days and mortality. Improves microcirculation, lower vasopressor use, PCT, lactate and leukocyte level and faster lactate clearance. No effect on long-term cognitive, psychological, and functional outcomes |
| **Statin**  (572-578) | 7 | 2010 - 2017 | 1469 (101) | Ward, ICU | No |  | Atorvastatin, simvastatin or rosuvastatin | Sepsis-1, sepsis-2, shock or not specified | Medical and/or surgical or general | SOFA, sepsis progressing to severe sepsis, length of ICU stay, mortality | Inflamma-tory levels, TLR4 surface expression levels | Different findings on cytokine levels, no effect on mortality, lower SOFA score, decreased TLR4 surface expression level, lower ventilator and vasopressor use, less progression to severe sepsis |
| **Thiamine**  (579) | 1 | 2023 | 88  (88) | ICU | No |  | Thiamine | Not specified | Medical |  | Serum creatinine | No difference |
| **Vitamin C**  (580-586) | 7 | 2014 - 2023 | 1196  (40) | ICU | No |  | Vitamin C | Sepsis-3, shock or not specified | Medical and/or surgical or general | Vasopressor use, SOFA score, incidence of VAP, safety, urine output, mortality or persistent organ dysfunction | CRP, microcircu-latory parameters | No effect on vasopressor use and incidence of VAP, safe and well tolerated. Different findings on SOFA score, no effect on CRP. Improvement of microcirculation and glycocalyx, no effect on urine output. Higher risk of death or persistent organ dysfunction. |
| **Vitamin C + thiamine**  (587) | 1 | 2021 | 50  (50) | ICU | No |  | Vitamin C and thiamine | Not specified | General |  | MMP-9 levels | Decrease in MMP-9 |

* Dose not specified or both low- and high-dose corticosteroids both included (low = <400mg/day or high >400mg/day hydrocortisone or equivalent). Abbreviations: AKI, acute kidney injury; CRP, ARDS, acute respiratory stress syndrome; C-reactive protein; ED, emergency department; (HR-)QoL, (health-related) quality of life; ICU, intensive care unit; IL, interleukin; IFN-γ, interferon- γ; MMP, Matrix metalloproteinases; LPS, lipopolysaccharides; PCT, procalcitonin; P/F ratio, PaO2/FiO2 ratio; PTSD, post-traumatic stress disorder; RCT, randomized controlled trial; RRT, renal replacement therapy; SOFA, sequential organ failure assessment; SRS, sepsis response signatures; sTREM-1, soluble triggering receptor expressed on myeloid cells-1; TIMP, tissue inhibitor of metalloproteinases; TLR-4, Toll-like receptor 4; TNF- α, tumor necrosis factor-α; VAP, ventilator acquired pneumonia.

**Supplementary Table 7. Immunonutrition**

| **Treatment** | **Number of studies** | **Year** | **Total patients (median per study)** | **Setting** | **Personali-zed approach** | **If yes, specifi-cation** | **Treatment** | **Sepsis definition** | | **Population** | | **Clinical primary endpoint** | | **Biochemical primary endpoint** | **Main findings** |
| --- | --- | --- | --- | --- | --- | --- | --- | --- | --- | --- | --- | --- | --- | --- | --- |
| **Observational studies** | | | | | | | | | | | | | | | |
| **Selenium**  (588) | 1 | 2013 | 72  (72) | ICU | No |  | Selenium | Sepsis-2 | | Medical and surgical | |  | | Routine biochemical and hematological parameters | No differences |
| **Interventional studies** | | | | | | | | | | | | | | | |
| ***RCTs*** | | | | | | | | | | | | | | | |
| **Amino acid enriched nutrition**  (589-591) | 3 | 2008-2015 | 185 (45) | ICU | No |  | Glutamine + calcium caseinate, alanine + glutamine, taurine | | Sepsis-1 | | General or medical | |  | Pro-albumin, CRP levels, IL-6, IL-10, WBC and glycolytic parameters | Lower CRP and IL-6 and, higher pro-albumin and IL-10, no difference in glycolytic parameters |
| **Antioxidants**  (592, 593) | 2 | 2020, 2023 | 228  (114) | ICU | No |  | Vitamin C, Vitamin E, NAC, Melatonin. antioxidants | | Sepsis-3, shock or unspecified | | Medical | | SOFA score | Inflammatory markers | Decreases SOFA score and inflammatory markers |
| **Dextrose**  (594) | 1 | 2021 | 58  (58) | ICU | No |  | Dextrose | | Sepsis-3 | | Medical | |  | IL-6 | No difference |
| **Fat emulsions/Fish oil**  (4, 5, 595-598) | 6 | 2003-2014 | 253  (36) | ICU | No |  | MCT/LCT-based fat emulsion, fish oil, omega-3 fatty acids, n-6 lipid infusion, medium-chain fatty acids and soybean oil, TPN containing 80% olive oil, 20% soy oil and fish oil | | Sepsis-1 or not specified | | Medical and/or surgical | | SOFA score, mortality and lenght of stay | Serum proteins, lipids, inflammatory markers | Reduction in SOFA score, not in length of stay. The effect on inflammatory markers and mortality differed per trial. |
| **Genistein**  (599) | 1 | 2023 | 32  (32) | ICU | No |  | Genistein | | Sepsis-3 | | Medical | |  | Inflammatory cytokines | Partly normalized inflammatory cytokines |
| **l-Carnitine**  (600) | 1 | 2019 | 21  (21) | ICU, ED | No |  | l-carnitine | | Sepsis-3, shock | | General (only males) | |  | Metabolites profiles | Elevated metabolite markers of vascular inflammation in the carnitine-treated non-survivors |
| **Immune-enhancing nutrition**  (3, 601) | 2 | 2000, 2008 | 236  (118) | ICU | No |  | Enteral feed enriched with arginine, mRNA, and fish oil or enteral pharmaconutrition supplement | | Sepsis-1, sepsis-2 | | Medical | | Mortality rate, SOFA score |  | Reduction in mortality rate and SOFA score |
| **Kukoamine B**  (602) | 1 | 2021 | 34  (34) | ICU | No |  | Kukoamine B | | Sepsis-3 | | General | | SOFA score |  | 0.24 mg/kg is recommended dose based on SOFA score decrease |
| **(Nano)**  **curcumin**  (603-606) | 4 | 2020-2022 | 160 (40) | ICU | No |  | (Nano)curcumin | | Not specified | | Medical and/or surgical | | SOFA score, nutritional status | Inflammatory markers, endothelial markers, full blood count and oxidative stress | Reduction in SOFA score, inflammatory markers, endothelial markers and oxidative stress, no difference in full blood count, nutritional status did not change. |
| **Selenium**  (6, 607-613) | 8 | 2007-2019 | 1693 (78) | ICU | No |  | Selenium (selenase or selenite) | | Sepsis-1, sepsis-2 or not specified | | General, medical and/or surgical | | Length of ICU stay, mortality, effect on RRT | Inflammatory markers | Reduction in length of ICU stay. No effect on the requirement of RRT. The effect on inflammatory markers and mortality differed per trial. |
| **Vitamin A**  (614) | 1 | 2018 | 64  (64) | ICU | No |  | Vitamin A | | Not specified | | General | | Length of ICU stay, days on ventilator, blood pressure support and mortality |  | No differences |
| **Vitamin D**  (615-617) | 3 | 2014-2018 | 197  (67) | ICU or not specified | No |  | Vitamin D, calcitriol, cholecalciferol | | Sepsis-1 or sepsis-2 | | Medical and/or surgical | |  | WBC, plasma cathelicidin protein, 25OHD and bioavailable 25OHD levels | Reduction in WBC and higher cathelicidin and 25OHD levels |
| **Vitamin E**  (618) | 1 | 2008 | 40  (40) | ICU | No |  | Alpha-tocopherol | | Sepsis-1 | | Medical | |  | Cytokine levels and gastric intramucosal pH | No differences |

Abbreviations: CRP, C-reactive protein; ED, emergency department; ICU, intensive care unit; IL, interleukin; LCT, long-chain triacylglycerols; MCT, medium-chain triacylglycerols; NAC, n-acetylcysteine; RCT, randomized controlled trial; RRT, renal replacement therapy; SOFA, sequential organ failure assessment; TPN, total parental nutrition; WBC, white blood cell.

**Supplementary Table 8. Concomitant treatments**

| **Treatment** | **Number of studies** | **Year** | **Total patients (median per study)** | **Setting** | **Persona-lized approach** | **If yes, specifi-cation** | **Treatment** | **Sepsis definition** | **Population** | **Clinical primary endpoint** | **Biochemical primary endpoint** | **Main findings** |
| --- | --- | --- | --- | --- | --- | --- | --- | --- | --- | --- | --- | --- |
| **Observational studies** | | | | | | | | | | | | |
| **Albumin or Crystalloids**  (619) | 1 | 2021 | 60  (60) | ICU | No |  | Albumin and crystalloid | Sepsis-3, shock | General | Oxidation status | Pentraxin 3, presepsin, chromogranin A | Reduction in SOFA score |
| **Dexmedetomi-dine**  (620) | 1 | 2010 | 63  (63) | ICU | No |  | Dexmedetomi-dine | Sepsis-2 | Surgical or medical | Delirium/coma-free days |  | Reduction in delirium/coma-free days, mortality and increase in ventilator-free days |
| **Interventional studies** | | | | | | | | | | | | |
| ***Non-RCTs*** | | | | | | | | | | | | |
| **L-NAME**  (7) | 1 | 1998 | 11  (11) | ICU | No |  | L-NAME | Sepsis-1 | Surgical | Mortality | Inflammatory markers | Decreased plasma TNF-R, Increase MAP and SVR |
| **Resuscitation**  (621) | 1 | 2008 | 66  (66) | ED, ICU | No |  | ARAS-2 Resuscitation | Sepsis-1, shock | Medical or surgical | Mortality | CRP, lactate | Decreased mortality |
| ***RCTs*** | | | | | | | | | | | | |
| **Dexmedetomi-dine** (8, 622-625) | 5 | 2018-2023 | 565  (80) | ICU or not specified | No |  | Dexmedetomidine | Sepsis-1, shock or not specified | Surgical, medical or general | AKI | Inflammatory markers | Different results on inflammatory markers, decreased AKI incidence and ICU duration |
| **Dexmedetomi-dine and propofol**  (626) | 1 | 2009 | 40  (40) | ICU | No |  | Dexmedetomi-dine and propofol | Sepsis-1 | Surgical | IAP | Inflammatory markers | Decreased inflammatory markers and IAP with dexmedetomidine |
| **Dopamine vs Dobutamine vs Norepinephrine**  (627) | 1 | 2021 | 75  (75) | ICU | No |  | Dopamine vs Dobutamine vs Norepinephrine | Not specified | General | Hemodynamics | Vascular elastic indexes, inflammatory markers | Norepinephrine showed the biggest effects in hemodynamics, vascular elasticity and lowering inflammatory markers |
| **Esmolol**  (628) | 1 | 2022 | 40  (40) | ED | No |  | Esmolol | Sepsis-1 | Medical | Hemody-namics | Inflammatory markers | Decreased CRP and oxygen consumption |
| **Glyemic control**  (629) | 1 | 2009 | 90  (90) | ICU | No |  | Glycemic control | Sepsis-2, shock | Surgical |  | Inflammatory markers, fibrinolysis | Enhanced fibrinolysis and decreased morbidity |
| **Levosimendan**  (9, 630-632) | 4 | 2019-2022 | 724 (89) | ICU | No |  | Levosimendan | Sepsis-1, sepsis-3 or not specified | Medical or general | Mortality, cardiac function, SOFA | Cardiac and inflammatory markers | No difference |
| **Lidocaine**  (633) | 1 | 2013 | 14  (14) | Ward, ICU | No |  | Lidocaine | Sepsis-2, shock | Medical or surgical |  | Chemokine-induced neutrophil arrest | Inhibition of chemokine-induced arrest and transmigration of neutrophils |
| **Midazolam**  (634) | 1 | 2015 | 104  (104) | ICU, Ward | No |  | Midazolam | Sepsis-2 | Burned patients |  | Inflammatory markers | Enhanced immune response |
| **Norepinephrine and terlipressin**  (635) | 1 | 2022 | 50  (50) | ICU | No |  | Norepinephrine and terlipressin | Sepsis-3 | Medical | MAP | Serum lactate, procalcitonin level, CRP | Decrease in serum lactate, SOFA, vasopressor |
| **Resuscitation**  (636) | 1 | 2017 | 226  (226) | ICU | No |  | 6% Hydroxyethyl starch | Sepsis-2 | Surgical or medical | Mortality | TNF- α, IL-6 and IL-10 | No difference |
| **Target temperature management**  (637, 638) | 2 | 2017, 2020 | 78 (38) | ICU, ED | No |  | Target temperature management | Sepsis-2, shock, sepsis-3 | Medical | ICU stay, anti-infection cost, survival rate, fever control | Inflammatory markers | Decreased pro-inflammatory cytokines in low temperature and ICU stay reduced with higher temperature control |
| **Therapeutic hyperthermia**  (639) | 1 | 2022 | 56  (56) | ICU | No |  | External Warming | Sepsis-2, sepsis-3 | Medical or surgical |  | HLA-DR, CD3/CD28, IFN | Decreased mortality and hospital free days, no significant changes in biomarkers |

Abbreviations: AKI, acute kidney injury; ARAS, advanced resuscitation algorithm for septic shock; CRP, C-reactive protein; ED, emergency department; IAP, intra-abdominal pressure; ICU, intensive care unit; IFN, interferon; IgM, immunoglobulin M; IL, interleukin; L-NAME, L-NG-Nitro arginine methyl ester; MAP, mean arterial pressure; MCU, medical care unit; RCT, randomized controlled trial; SOFA, sequential organ failure assessment; SVR, systemic vascular resistance; TNF- α, tumor necrosis factor- α; TNF-R, tumor necrosis factor-receptor.

**Supplementary Table 9. Traditional Chinese Medicine**

| **Treatment** | **Number of studies** | **Year** | **Total patients (median per study)** | **Setting** | **Personali-zed approach** | **If yes, specifi-cation** | **Treatment** | **Sepsis definition** | **Population** | **Clinical primary endpoint** | **Biochemical primary endpoint** | **Main findings** |
| --- | --- | --- | --- | --- | --- | --- | --- | --- | --- | --- | --- | --- |
| **Observational studies** | | | | | | | | | | | | |
| **Xuebijing injection**  (640) | 1 | 2017 | 49  (49) | ICU | No |  | Xuebijing injection | Sepsis-2 | Medical |  | Inflammatory markers | Decreased inflammatory markers |
| **Interventional studies** | | | | | | | | | | | | |
| ***RCTs*** | | | | | | | | | | | | |
| **Electroacupuncture**  (641, 642) | 2 | 2018 - 2022 | 144  (72) | ICU | No |  | Electroacu-puncture | Sepsis-3, Sepsis-3 Shock | Medical | Inflammatory markers | Inflammatory markers | Decreased APACHE II score and inflammatory biomarkers |
| **Herbal extract**  (643) | 1 | 2012 | 29  (29) | ICU | No |  | Septimeb | Sepsis-2 or not specified | Surgical or medical | Inflammatory markers, mortality | IL-2, TNF- α, total antioxidant power, Matrix Metalloproteinase2, Platelet Growth Factor | Decreased mortality, SAPS, SOFA, APACHE II |
| **JinHong Formula** (644) | 1 | 2023 | 114  (114) | ICU, ED | No |  | JinHong Formula | Sepsis-3 | Medical | SOFA, mortality | IL-6, TNF- α, superoxide dismutase, IL-17, PCT | Reduced mortality and SOFA score |
| **Shenfu injection**  (10, 645) | 2 | 2017, 2022 | 197 (99) | ICU | No |  | Shenfu injection | Sepsis-3, Shock | Medical or general | Length of ICU stay, duration of vasopressor use, illness severity, organ dysfunction, mortality | Inflammatory markers, lactate | Decreased vasopressor use, APACHE II and Marshall score |
| **Shenling Chengqi Decoction**  (646) | 1 | 2022 | 160  (160) | ICU | No |  | Shenling Chengqi Decoction | Not specified | General | GI symtpoms and imflammatory markers | Inflammatory markers | Reduction in GI symptoms and observation scores, increase in defecation |
| **Shufeng Jiedu**  (647) | 1 | 2020 | 129  (129) | ICU | No |  | Shufeng Jiedu | Sepsis-1 | Medical | APACHE II score | PCT, CRP, WBC | Improved APACHE II, TCM symptom score, healing rate and decreased inflammatory biomarkers |
| **Xuebijing injection**  (11, 648, 649) | 3 | 2014 - 2023 | 2052  (171) | ICU, Ward | No |  | Xuebijing injection | Sepsis-2, sepsis-3 | Medical | DIC, mortality | Inflammatory markers | Decreased DIC incidence, mortality, APACHE II, length of stay, invasive ventilation and inflammatory markers |
| **Xinmailong infusion**  (650) | 1 | 2021 | 192  (192) | ICU | No |  | Xinmailong infusion | Sepsis-3, Shock | Surgical or medical | Incidence of sepsis-induced myocardial dysfunction | BNP | Decrease in diastolic SIMD, increase in BNP |

Abbreviations: APACHE, Acute Physiology And Chronic Health Evaluation; BNP, brain natriuretic peptide; CRP, C-reactive protein; DIC, disseminated intravascular coagulation; ED, emergency department; GI, gastro-intestinal; ICU, intensive care unit; IL, interleukin; PCT, procalcitonin; RCT, randomized controlled trial; SAPS, subacromial pain syndrome; SIMD, sepsis-induced myocardial dysfunction; SOFA, sequential organ failure assessment; TCM, traditional Chinese medicine; TNF- α, tumor necrosis factor- α; WBC, white blood cell.

**Supplementary Table 10. Immunostimulatory cytokines and growth factors**

| **Treatment** | **Number of studies** | **Year** | **Total patients (median per study)** | **Setting** | **Personali-zed approach** | **If yes, specifi-cation** | **Treatment** | **Sepsis definition** | **Population** | **Clinical primary endpoint** | **Biochemical primary endpoint** | **Main findings** |
| --- | --- | --- | --- | --- | --- | --- | --- | --- | --- | --- | --- | --- |
| **Observational studies** | | | | | | | | | | | | |
| **G-CSF**  (651, 652) | 2 | 2002, 2004 | 78 (39) | ICU | No |  | G-CSF | Septic shock | General | Mortality, safety |  | Safe to use, reduction in mortality, meliodosis patients particular benefit |
| **Interventional studies** | | | | | | | | | | | | |
| ***Non-RCTs*** | | | | | | | | | | | | |
| **G-CSF**  (653-655) | 3 | 1994  -  2000 | 74  (24) | ICU, ward | Yes, two studies (653, 655) | Neutropenia or granulocytopenia | Filgrastim, rhG-CSF | Sepsis-1 or not specified | Surgical, neutropenic, or general | Safety | Leukocyte counts, CRP, IL-6, IL-8, TNF- a, IL-1 b, M-CSF, G-CSF, IFN-γ, and sIL-2r | Granuclocyte increase associated with survival; leukocyte counts increased; GCF increased; no adverse events |
| **GM-CSF**  (656) | 1 | 2003 | 9  (9) | ICU | No |  | rhGM-CSF | Sepsis-1 | Medical |  | HLA-DR expression | Increase in HLA expression |
| **Granulocyte infusion**  (657) | 1 | 1972 | 76  (76) | Not specified | No |  | PMN transfusion | Not specified | Neutropenic | Survival |  | Reduced mortality |
| **Interferon γ-1b**  (658) | 1 | 1997 | 10  (10) | ICU | Yes  (658) | Based on HLA-DR expression | Interferon γ-1b | Not specified | Surgical and/or medical |  | HLA-DR expression | Increased HLA-DR expression |
| **Leucocyte transfusions** (659) | 1 | 1999 | 30  (30) | Ward or ICU | Yes  (659) | Neutropenic patients | Leucocyte transfusions from rhG-CSF or prednisolone stimulated donors | Not specified | Immuno-compromised neutropenic patients |  | Leucocyte concentrates | Increase in leucocyte and ANC |
| ***RCTs*** | | | | | | | | | | | | |
| **G-CSF**  (660-665) | 6 | 2001  -  2008 | 999 (45) | ICU, Ward | No |  | Filgrastim, lenograstim, G-CSF | Sepsis-1, shock | Surgical and/or medical | Mortality, safety | Cytokines, endotoxins, Leukocyte counts, CRP, thrombomodulin | No mortality reduction; increased leukocyte count; decreased CRP |
| **GM-CSF**  (666-671) | 6 | 2002  -  2023 | 379 (48) | ICU, ward | Yes, three studies  (666, 669, 670) | HLA-DR guided therapy | GM-CSF, mol-gramostim | Sepsis-1 or not specified | Medical or surgical | Mortality, LOS, organ support, ICU-acquired infection | mHLA-DR increase, cytokine levels, reduction in absolute neutrophil count | No survival benefit or effect on ICU-acquired infections. Improved PF ratio and absolute neutrophil count. Shorter duration of ventilation; shorter LOS; HLA-DR normalization |
| **Granulocyte transfusion**  (672) | 1 | 1977 | 15  (15) | Not specified | No |  | Granulocyte transfusion | Not specified | Neutropenic | Mortality |  | Mortality reduction |
| **IL-7**  (673, 674) | 2 | 2018  ,  2023 | 48  (24) | ICU | Yes, both studies  (673, 674) | Lymphopenic patients | CYT107 (glycosylated recombinant human IL-7) | Sepsis-1, sepsis-2 | Medical | Safety | Lymphocyte counts; circulating CD4+, CD8+ cells | Increased lymphocyte counts and circulating T cells. Transient respiratory distress when administerd intramuscular. Intravenous well tolerated. |
| **Thymosin α-1**  (675) | 1 | 2013 | 361  (361) | ICU | No |  | Ta1 (Zadaxin) | Not specified | Medical | Mortality | mHLA-DR | No difference in mortality reduction. Increase HLA-DR. |

Abbreviations: APACHE, Acute Physiology And Chronic Health Evaluation; CRP, C-reactive protein; (rh)G(M)-CSF, (recombinant human)granulocyte(-macrophage) colony-stimulating factor; ICU, intensive care unit; IFN- γ, interferon- γ; (m)HLA-DR, (monocyte) Human Leukocyte Antigen-DR; IL, interleukin; LOS, length of stay; PF ratio, pO2/FiO2 ratio; RCT, randomized controlled trial; sIL-2r, soluble interleukin-2 receptor; TNF- α, tumor necrosis factor- α.

**Supplementary Table 11. Intravenous immunoglobulins**

| **Treatment** | **Number of studies** | **Year** | **Total patients (median per study)** | **Setting** | **Personali-zed approach** | **If yes, specifi-cation** | **Treatment** | **Sepsis definition** | **Population** | **Clinical primary endpoint** | **Biochemical primary endpoint** | **Main findings** |
| --- | --- | --- | --- | --- | --- | --- | --- | --- | --- | --- | --- | --- |
| **Observational studies** | | | | | | | | | | | | |
| **Ig**  (676-680) | 5 | 2015  - 2023 | 5760  (239) | ICU | Yes, one study (678) | Low IgG levels | Ig | Sepsis-1, shock, Sepsis-3, shock or unspecified | Surgical, medical and/or general | Mortality | Il6, CRP | Conflicting results on mortality, IL6 and CRP decreased |
| **IgG**  (681-684) | 4 | 1991  -  2021 | 3538  (132) | ICU, ED | No |  | IgG | Sepsis-1, shock, Sepsis-3 | DIC, medical and/or surgical | Mortality, APACHE II scores, SOFA scores, DIC scores | Infection and coagulation related markers | No mortality reduction; TNF was higher in intervention group, clinical severity indices decreased |
| **IgM enriched Ig**  (685-690) | 6 | 2005  -  2022 | 1560 (83) | ICU | No |  | (Polyclonal) IgM enriched Ig | Sepsis-1, Sepsis-2, sepsis-3 | Surgical, medical and/or general | Mortality, SOFA score | Biomarker levels | Lower LOS. Mortality reduction in MDR gram negative infections. In general sepsis population conflicting results on mortality reduction, conflicting results on SOFA. No differences in NO, endocan, pentraxin and presepsin biomarkers. |
| **IgMA enriched Ig**  (691, 692) | 2 | 2004, 2021 | 76 (38) | ICU | No |  | Ig preparation enriched in IgM and IgA | Sepsis-1,  shock, Sepsis-3, shock | General | Difference in patient characteristics between survivors and nonsurvivors | IgM and IgA levels | No differences found in patient characteristics; IgM increase in survivors; IgA increase in survivors and non-survivors; IgG the same. |
| **Interventional studies** | | | | | | | | | | | | |
| ***Non-RCTs*** | | | | | | | | | | | | |
| **IgG**  (693, 694) | 2 | 1989, 2020 | 87  (44) | ICU or not specified | No |  | IgG | Sepsis-2 or not specified | Medical |  | Levels of serum IgG, IgA, IgM, PAI-1, and protein C. | Higher serum IgM levels in IVIG group. Lower PAI-1 levels; increased Protein C levels. |
| **IgM enriched Ig**  (695, 696) | 2 | 1992, 2016 | 47  (24) | ICU, Ward | Yes, one study (695) | Neutropenic patients | IgM-IVIg, human polyclonal IgM-enriched immunoglobulin preparation (Pentaglobin) | Sepsis-2 or not specified | Surgical or neutropenic |  | Endotoxin activity and IgM and IgG antibodies against lipid A and LPS | Endotoxin activity decreased |
| ***RCTs*** | | | | | | | | | | | | |
| **Ig**  (697-701) | 5 | 1981  -  2013 | 268 (55) | ICU, ward or not specified | Yes, one study (698) | Thrombocytopenia | Polyglin; endobulin; Pentaglobin; pepsin-treated human gamma globulin (gamma-venin) | Sepsis-1 or not specified, shock | General, surgical and/or medical | Mortality; SOFA score | Inflammatory markers; platelet count | Conflicting results on mortality; lower endotoxin titers; differences in SIRS; IL6 and blood lactate levels in high dose group |
| **IgG**  (702-706) | 5 | 1988  -  2008 | 873 (62) | ICU | No |  | ivIgG; Polyglin; endobulin; polyvalent IgG with stabilized Fc sub- units | Sepsis-1, sepsis-2, or not specified | General, surgical and/or medical | Mortality | Il6, TNF | Conflicting results on mortality; lower death rate septic shock or more severe sepsis; no biomarker effects |
| **IgG AB to E. Coli**  (707) | 1 | 1988 | 71  (71) | ICU | No |  | J5-IVIG vs IVIG | Not specified | Medical and/or surgical | Mortality; shock reversal |  | No differences |
| **IgM Ab**  (708, 709) | 2 | 1991 | 1312 (656) | ICU or not specified | No |  | Murine monoclonal Ab directed against gram-negative endotoxin (E5); MAB-T88 | Sepsis-1 or not specified | Medical | Mortality, safety, organ failure |  | Conflicting results on mortality. More adverse events in treatment group. |
| **IgMA-enriched Ig**  (710) | 1 | 2006 | 211  (211) | Unspecified | Yes, one study (710) | Neutropenic patients | Intravenous IgMA-enriched immunoglobulin | Sepsis-1 | Neutropenic | Mortality |  | No mortality reduction |
| **IgM enriched Ig**  (711-715) | 5 | 2002  -  2019 | 188 (38) | ICU | No |  | Pentaglon; IgM-enriched immunoglobulin; | Sepsis-1, sepsis-2, or not specified | General, surgical and/or medical | Mortality, SOFA, microperfusion | PCT | No clinical outcome effects; increased microperfusion; decreased PCT |
| **IgGAM**  (716) | 1 | 2018 | 160  (160) | ICU | Yes  (716) | Subgroup analyses in patients with different CRP and IgM levels | Trimodulin | Not specified | Medical | Ventilator free days |  | No differences in general population; significant reductions in mortality in patients with high CRP, low IgM, and high CRP/low IgM at baseline. |

Abbreviations: Ab, antibody; APACHE, Acute Physiology And Chronic Health Evaluation; CRP, C-reactive protein; DIC, disseminated intravascular coagulation; E, enterococcus; ED, emergency department; g-CSF, granulocyte colony-stimulating factor; ICU, intensive care unit; IFN- γ, interferon- γ; IL, interleukin; Ig, immunoglobulins; LPS, lipopolysaccharides; LOS, length of stay; MDR, multi-drug resistant; NO, nitric oxide; PAI-1, plasminogen activator inhibitor-1; PCT, procalcitonin; RCT, randomized controlled trial; SIRS, systemic inflammatory response syndrome; SOFA, sequential organ failure assessment; TNF, tumor necrosis factor.

**Supplementary Table 12. Mesenchymal stem cells**

| **Treatment** | **Number of studies** | **Year** | **Total patients (median per study)** | **Setting** | **Personali-zed approach** | **If yes, specifi-cation** | **Treatment** | **Sepsis definition** | **Population** | **Clinical primary endpoint** | **Biochemical primary endpoint** | **Main findings** |
| --- | --- | --- | --- | --- | --- | --- | --- | --- | --- | --- | --- | --- |
| **Observational studies** | | | | | | | | | | | | |
| **Mesenchymal stem cells**  (717) | 1 | 2023 | 30  (30) | ICU | No |  | Bone marrow-derived MSCs | Not specified | Medical |  | Metabolite change | Effect on on serum metabolic change |
| **Interventional studies** | | | | | | | | | | | | |
| ***Non-RCTs*** | | | | | | | | | | | | |
| **Mesencymal stromal cells**  (718-721) | 4 | 2018  -  2022 | 105  (30) | ICU | No |  | MSCs | Sepsis-2, Sepsis-3, shock or not specified | General, surgical and/or medical | Safety, tolerability, mortality, SOFA | Cytokine levels | No mortality effect; decrease SOFA score. No significant cytokine changes. Safe. |
| ***RCTs*** | | | | | | | | | | | | |
| **Mesenchymal stem cells**  (722, 723) | 2 | 2018  ,  2024 | 113  (57) | ICU | Yes, one study (722) | Neutropnic patients | Bone marrow-derived MSCs, allogeneic adipose-derived mesenchymal stem cell | Sepsis-2 or not specified | Medical | Safety |  | Different results on survival rates, well tolerated. |

Abbreviations: ICU, intensive care unit; MSCs, mesenchymal stromal cells; RCT, randomized controlled trial; SOFA, sequential organ failure assessment.

**Supplementary Table 13. Immune-checkpoint inhibitors**

| **Treatment** | **Number of studies** | **Year** | **Total patients (median per study)** | **Setting** | **Personali-zed approach** | **If yes, specifi-cation** | **Treatment** | **Sepsis definition** | **Population** | **Clinical primary endpoint** | **Biochemical primary endpoint** | **Main findings** |
| --- | --- | --- | --- | --- | --- | --- | --- | --- | --- | --- | --- | --- |
| **Interventional studies** | | | | | | | | | | | | |
| ***Non-RCTs*** | | | | | | | | | | | | |
| **Anti-PD-1 antibody**  (724) | 1 | 2020 | 13  (13) | ICU | Yes (724) | Lymphocyte count of ≤1,100/μL | Nivolumab | Sepsis-3 | Medical | Safety | Lymphocyte counts; mHLA-DR | One drug related AE; lymphocyte count and mHLA-DR increased over time |
| ***RCTs*** | | | | | | | | | | | | |
| **Anti-PD-1 antibody**  (725, 726) | 2 | 2019 | 55 (28) | ICU | Yes, two trials  (725, 726) | Immunosuppressed patients | Nivolumab/BMS-936559 | At least one of three organ dysfunction criteria: hypotension; respiratory failure or AKI. | Medical and/or surgical | Safety and tolerability | Immune status | Well tolerated, with no evidence of drug-induced hypercytokinemia or cytokine storm, and at higher doses, some indication of restored immune status over 28 days. |

Abbreviations: AE, adverse event; anti-PD-1, anti-programmed cell death protein 1; AKI, acute kidney injury; ICU, intensive care unit; (m)HLA-DR, (monocyte) Human Leukocyte Antigen-DR; RCT, randomized controlled trial.

**Supplementary Table 14. Combinations of therapies**

| **Treatment** | **Number of studies** | **Year** | **Total patients (median per study)** | **Setting** | **Personali-zed approach** | **If yes, specifi-cation** | **Treatment** | **Sepsis definition** | **Population** | **Clinical primary endpoint** | **Biochemical primary endpoint** | **Main findings** |
| --- | --- | --- | --- | --- | --- | --- | --- | --- | --- | --- | --- | --- |
| **Observational studies** | | | | | | | | | | | | |
| **Antibiotics and/or IVIG**  (727, 728) | 2 | 2014,  2022 | 141 (71) | ICU, not specified | No |  | Clindamycin and/or IVIG | Sepsis-3 or not specified | Medical | Mortality and case fatality rate |  | Clindamycin reduced mortality; IVIG did not |
| **Corticosteroids and/or vasopressors**  (729-734) | 6 | 2009  -  2023 | 2086  (178) | ICU | No |  | Vasopressin or hydrocortisone; vasopressin + corticosteroids or norepinephrine + corticosteroids; AVP + hydrocortisone; noradrenaline and vasopressin, with and without hydrocortisone | Sepsis-1, shock, sepsis-2, shock, sepsis-3, shock or not specified | Medical or surgical | Mortailty, severe adverse events (new onset arrhythmia, hyperglycemia, hyponatremia, or superinfection), shock reversal | Cytokine trajectories over 3 days | Lower adverse event rate with vasopressin; different results on mortality with vasopressin + corticosteroids; no difference with AVP + hydrocortisone; hydrocortisone has anti-cytokine effect, faster shock reserval with vasopressin + corticosteroids |
| **Drotrecogin alfa (activated) and/or steroids**  (735, 736) | 2 | 2005, 2013 | 1120 (560) | ICU | No |  | Drotrecogin alfa (activated) and/or steroids | Sepsis-1, Sepsis-3, shock | General | Mortality |  | Conflicting result on mortality, regardless of steroid use |
| **Patient tailored protocol*** (13) | 1 | 2020 | 114  (114) | ICU | Yes  (13) | Patient tailored* | Vasopressor | Sepsis-3 | General | ICU-mortality | Inflammatory markers | Reduction in ICU-mortality |
| **Prostaglandin E with continuous renal replacement therapy**  (737) | 1 | 2020 | 114  (114) | ICU | No |  | Prostaglandin E with continuous renal replacement therapy | Sepsis-2 | Patients with AKI |  | Urinary augmenter of liver regeneration, urinary Na+/H+ exchanger 3, inflammatory cytokines | Improved renal function and reduced inflammatory responses |
| **Resuscitation, antibiotic, corticosteroids and/or drotrecogin alfa**  (738) | 1 | 2009 | 2796  (2796) | ICU | No |  | Early broadspectrum antibiotics, fluid challange, low-dose steroids and/or drotrecogin alfa | Sepsis-2 | Medical and/or surgical | Mortality |  | Early administration of broadsprectum AB in all patients and drotrecogin alfa in the most severe patients reduced mortality |
| **Oxymatrine and entecavir**  (739) | 1 | 2022 | 90  (90) | Not specified | No |  | Oxymatrine and entecavir | Sepsis-1 | Medical | Clinical efficacy |  | Improved clinical efficacy |
| **Statin and/or hemoperfusion**  (740) | 1 | 2012 | 173  (173) | ICU | No |  | Statin and/or hemoperfusion with PMX-F | Sepsis-1, shock | Medical | Mortality |  | Statin use and PMX-F treatment independently correlated to 28-day survival |
| **Thrombomodulin and Polymyxin B Hemoperfusion**  (741) | 1 | 2020 | 2350  (2350) | ICU | No |  | ART‑123 and PMX-HP | Sepsis-1, shock | Medical | Mortality |  | Lower mortality with ART-123, benefical effect of ART-123 and PMX-HP treatment |
| **Interventional studies** | | | | | | | | | | | | |
| ***Non-RCTs*** | | | | | | | | | | | | |
| **Thrombomodulin and Polymyxin B Hemoperfusion**  (742) | 1 | 2013 | 22  (22) | ICU | No |  | Combination Therapy of Polymyxin-B Direct and Hemoperfusion and Recombinant Thrombomodulin | Sepsis-1 | Patients with sepsis-induced DIC | Mortality |  | Lower mortality |
| ***RCTs*** | | | | | | | | | | | | |
| **Anakinra or interferon-γ**  (14) | 1 | 2022 | 240  (240) | Not specified | Yes  (14) | Identifi-cation of either immune-paralysis or hyperin-flammation; treatment based on phenotype | Anankinra or interferon-γ | Sepsis-3 | Medical | Mortality |  | Increased 7-day survival, no difference at 28-days |
| **Traditional Chinese Medicine and thymosin α1**  (743) | 1 | 2022 | 81  (81) | Not specified | No |  | Thymosin α1 and Xuebijing | Sepsis-3 | Medical | Hemodynamics | Inflammatory biomarkers | Improved hemodynamics and decreased inflammatory biomarkers |
| **Dexamethasone, colchicine and pentoxifylline**  (744) | 1 | 1997 | 26  (26) | Not specified | No |  | Dexamethasone, colchicine and pentoxifylline | Not specified | Medical |  | TNF-α levels | Decreased TNF-α levels |
| **Corticosteroids and/or vasopressors**  (745-747) | 3 | 2014  ,  2023 | 533  (63) | ICU | No |  | Vasopressin and hydrocortisone, vasopressin and placebo, norepinephrine and hydrocortisone, or norepinephrine and placebo | Sepsis-1, shock or not specified | Medical and/or surgical | Kidney failure–free days during in 28-days, vasopressin dosage and duration | Plasma vasopressin concentration | Plasma vasopressin concentration were not different in the hydrocortisone group compared to placebo, patients treated with hydrocortisone required lower and shorter dose of vasopressin; vasopressin compared with norepinephrine did not improve the number of kidney failure–free days |
| **Rhubarb and/or ulinastatin**  (748) | 1 | 2019 | 75  (75) | ICU | No |  | Rhubarb and/or ulinastatin | Not specified | Medical | APACHE II score, organ function | Inflammatory biomarkers, tissue perfusion | All combinations decreased CRP, WBC, lactate and APACHE II scores, ulinastatin + rhubarb also lowered PCT |
| **Thymosin α1 and blood purification**  (749) | 1 | 2022 | 86  (86) | ED | No |  | Thymosin α1 and blood purification | Not specified | Medical | Duration of shock, length of ICU stay, adverse reactions | Decreased duration of shock and length of ICU stay; no difference in adverse reactions | Thymosin α1 and blood purification |
| **Ulinastatin and norepinephrine**  (750) | 1 | 2022 | 100  (100) | Ward | No |  | Norepinephrine and ulinastatin | Not specified | General | Time for shock improvement, ICU and hospital stay, mortality | Inflammatory biomarkers | Positive effect on all outcomes |
| **Ulinastatin and alanyglutamine**  (751) | 1 | 2020 | 84  (84) | Not specified | No |  | Ulinastatin and alanyl glutamine | Sepsis-3, shock | General | APACHE II score, multiple organ dysfunction syndrome, mortality | Inflammatory biomarkers | Positive effect on all outcomes, except mortality |
| **Ulinastatin and continuous renal replacement**  (752) | 1 | 2017 | 106  (106) | ICU | No |  | Ulinastatin and continuous renal replacement | Sepsis-3 | Patients with AKI |  | Inflammatory cytokines, oxidative stress level, kidney and blood coagulation functions | Decrease in inflammatory cytokines, improved coagulation and kidney function |
| **Ulinastatin and thymosin α1**  (12, 753-756) | 5 | 2008  -  2019 | 428 (70) | ICU | No |  | Ulinastatin and thymosin α1 | Sepsis-1, sepsis-2 | Medical and/or surgical or not specified | Mortality; cardiopulmonary function | Inflammatory biomarkers | Decreased mortality; better cardiopulmonary function; decreased inflammatory biomarkers |
| **Ulinastatin or metabolic resuscitation**  (757) | 1 | 2021 | 60  (60) | ICU | No |  | Ulinastatin or hydrocortisone, ascorbic acid and thiamine | Not specified | General | SOFA score |  | Lower SOFA score in ulinastatin group |

*The patient-tailored therapy protocol included the use of IgM-enriched immunoglobulins for patients with low plasma IgM levels, blood purification strategies for patients with high plasma levels of cytokines or endotoxin, albumin correction and modulation of vasoactive agents. Abbreviations: AB, antibiotics; AKI, acute kidney injury; ANC, absolute neutrophil count; APACHE, Acute Physiology And Chronic Health Evaluation; AVP, arginine-vasopressin; CRP, C-reactive protein; ED, emergency department; (rh)G-CSF, (recombinant human) granulocyte colony-stimulating factor; ICU, intensive care unit; IVIG, intravenous immunoglobulins; PCT, procalcitonin; PMX-F, polymyxin B-immobilized fiber; PMX-HP, polymyxin B immobilised haemoperfusion; RCT, randomized controlled trial; SOFA, sequential organ failure assessment; WBC, white blood cell.

**Supplementary Table 15. Other therapies**

| **Treatment** | **Number of studies** | **Year** | **Total patients (median per study)** | **Setting** | **Personali-zed approach** | **If yes, specifi-cation** | **Treatment** | **Sepsis definition** | **Population** | **Clinical primary endpoint** | **Biochemical primary endpoint** | **Main findings** |
| --- | --- | --- | --- | --- | --- | --- | --- | --- | --- | --- | --- | --- |
| **Observational studies** | | | | | | | | | | | | |
| **Channa striata**  (758) | 1 | 2023 | 44  (44) | ICU | Yes (758) | hypoalbuminemia < 4 g/dl | Channa striata (albumin extract) | Unspecified | Medical |  | Syndecan-1 levels | Decrease in syndecan-1 levels |
| **Paracetamol** (759) | 1 | 2023 | 50  (50) | ICU | Yes (759) | two polymorphisms that encode for proteins related to the hepatic metabolism of paracetamol | Paracetamol | Unspecified | Medical |  | Plasma levels of paracetamol | Patients carrying CYP3A5 rs776746 A/G genotypes showed significantly higher plasma levels |
| **Interventional studies** | | | | | | | | | | | | |
| ***RCTs*** | | | | | | | | | | | | |
| **Alkaline phosphatase**  (15, 760-762) | 4 | 2009  -  2018 | 409 (36) | ICU | No |  | Alkaline phosphatase | Sepsis-1, sepsis-2 | Medical and/or surgical (in two trial in AKI patients) | Renal function; safety | Pharmocakynetics | Lower creatinine and NO metabolite production, different findings on renal function; linear, dose-proportional pharmacokynetics |
| **Edaravone**  (763) | 1 | 2016 | 60  (60) | ICU | No |  | Edaravone | Sepsis-1 | Medical |  | NFKB, MAPK, HSP72 and TAC | Decrease in NFKB and MAPK, increase in HSP72 and TAC |
| **Homeopathic treatment**  (764) | 1 | 2005 | 70  (70) | ICU | No |  | Homeopathic treatment (homeopathic doctors were free to decide which should be applied) | Sepsis-1 | Medical | Mortality |  | Lower mortality |
| **Melatonin and/or propolis**  (765) | 1 | 2022 | 55  (55) | ICU | No |  | Propolis, melatonin or both | Sepsis-1 | Medical | Mortality | Inflammatory markers | Melatonin + propolis reduced IL-6 and CRP, no difference in mortality |
| **Methylene blue**  (766) | 1 | 2002 | 30  (30) | ICU | No |  | Methylene blue | Sepsis-1 | Medical and/or surgical |  | Cytokine levels | No difference |
| **N-acetylcysteine**  (767-769) | 3 | 2003  -  2014 | 112 (39) | ICU | No |  | N-acetylcysteine | Sepsis-1 | Medical and/or surgical | Duration of ventilation, intensive care unit stay, ICU-mortality | Cytokines and gastric intramucosal pH, nuclear factor- B activation and circulating cytokine and adhesion molecules | Different results of cytokine responses, no difference in the other outcomes |
| **Physostigmine**  (770) | 1 | 2019 | 20  (20) | ICU | No |  | Physostigmine | Sepsis-1, shock | Surgical | Safety and tolerability |  | Safe and tolerable treatment |
| **Talactoferrin**  (771, 772) | 2 | 2013,  2015 | 499 (250) | ICU, ED | No |  | Talactoferrin | Sepsis-1 | Medical | Mortality and tolerability |  | Different results on mortality, tolerable treatment |
| **Vagus nerve stimulation**  (773) | 1 | 2023 | 20  (20) | ICU | No |  | Transcutaneous auricular vagus nerve stimulation | Sepsis-3 | Medical | SOFA and APACHE II score | Cytokine levels | Positive effect on cytokine levels, no effect on SOFA and APACHE II score |

Abbreviations: AKI, acute kidney injury; APACHE, Acute Physiology and Chronic Health Evaluation; CRP, C-reactive protein; ED, emergency department; ICU, intensive care unit; IL, interleukin; HSP72, heat shock protein 72; MAPK, mitogen-activated protein kinase; NFKB, nuclear transcription factor kappa B activity; NO, nitric oxide; RCT, randomized controlled trial; SOFA, and Sequential Organ Failure Assessment; TAC, total antioxidant capacity.

**Supplementary Table 16. Ongoing trials**

| **Treatment group** | **Treatment** | **Number of studies** | **Recruitment status** | **Number of patients** | **Setting** | **Perso-nalized ap-proach** | **If yes, specifi-cation** | **Treatment** | **Sepsis definition** | **Population** | **Clinical primary endpoint** | **Biochemical primary endpoint** | **NCT number** |
| --- | --- | --- | --- | --- | --- | --- | --- | --- | --- | --- | --- | --- | --- |
| **Observational studies** | | | | | | | | | | | | | |
| **Non-pharmalo-gical** | Continuous Renal Replacement Therapy | 1 | Not yet recruiting | 98 | Not specified | No |  | CRRT with oXiris membrame | Not specified | Septic AKI patients requiring RRT | Mortality, VT and intradialytic hypotension |  | NCT05575024 |
|  | Cytokine adsorption | 2 | Recruiting | 160 | ICU, not specified | Yes, in one study (NCT04306419) | IL-6 > 1000 pg/ml, IL-10 levels >250 pg/ml or TNF-α levels > 100 ng/l | Cytokine adsorption | Sepsis-3 or not specified | Need for CRRT or ECMO or HIV | Mortality | IL-6, IL-8, IL-10, TNF- α | NCT04306419, NCT06145828 |
|  | Hemoperfusion | 1 | Recruiting | 300 | ICU | Yes | IL-6 >25 pg/ml and leukocytes >15x10 9/l, CRP >40 mg/l, procalcitonin >0.9 mg/l | Hemoperfusion | Not specified | ARDS | Mortality |  | NCT05608096 |
|  | Immune Cell Extracorporeal Therapy | 1 | Recruiting | 142 | Not specified | No |  | Immune Cell Extracorporeal Therapy | Sepsis-3 | General | Long-term mortality |  | NCT06143137 |
| **Immunonu-trition** | Dietary Fiber | 1 | Active, not recruiting | 2 | ICU | No |  | Dietary fiber nutrients | Sepsis-3 | General |  | Changes of bone metabolic markers | NCT05623098 |
| **Concomitant treatments** | Fluid therapy | 1 | Recruiting | 60 | ICU | No |  | Saline vs. albumin | Not specified | General | Peripheral tissue hypoperfusion |  | NCT05094856 |
| **Immunostimulatory**  **cytokines & growth**  **factors** | Thymosin α1 | 1 | Not yet recruiting | 900 | Not specified | No |  | Thymosin α1 | Sepsis-3 | General | Long-term mortality |  | NCT04901104 |
| **Interventional studies** | | | | | | | | | | | | | |
| ***Non-RCTs*** | | | | | | | | | | | | | |
| **Non-pharmalo-gical** | Therapeutic plasma exchange | 1 | Recruiting | 54 | ICU | No |  | Therapeutic plasma exchange | Sepsis-2 & sepsis-3 | General | Adverse events |  | NCT04057872 |
| **Mesenchymal stem cells** | Mesenchymal stromal cells | 1 | Not yet recruiting | 21 | Not specified | No |  | Advanced Mesenchymal Enhanced Cell Therapy | Not specified | Bacterial sepsis | Safety and maximum feasible tolerated dose |  | NCT04961658 |
| **Other** | DNase I | 1 | Active, not recruiting | 36 | ICU | No |  | DNase I | Sepsis-3 | General |  |  | NCT05453695 |
| ***RCTs*** | | | | | | | | | | | | | |
| **Innate immune response** | Anakinra | 1 | Recruiting | 60 | Not specified | Yes | Presepsin > 350 pg/ml | Anakinra | Adjusted sepsis-3 (quick SOFA) | Pneumonia | SOFA score |  | NCT05785442 |
|  | Apoptotic cells | 1 | Recruiting | 160 | ICU | No |  | Allocetra-OTS | Sepsis-3 | CAP, UTI, abdominal infections | SOFA score, safety |  | NCT04612413 |
|  | Neutrophil Elastase Inhibitor | 2 | Not yet recruiting or recruiting | 338 | ICU | No |  | Sivelestat sodium | Sepsis-3 or not specified | ARDS caused by sepsis or general | Mechanical ventilation time, progression to ARDS |  | NCT05672472, NCT04973670 |
|  | Ulinastin | 2 | Not yet recruiting or recruiting | 295 | ICU | No |  | Ulinastin | Sepsis-3 or not specifed | General | SOFA score, multiple organ dysfunction, mortality |  | NCT05391789, NCT05895240 |
| **Non-pharmalo-gical** | CytoSorb | 2 | Recruiting | 192 | Not specified | Yes, both trials | IL-6 ≥ 1000 ng/l and need for CRRT | CytoSorb | Not specified | General | Change in noradrena-line | Catecholamine dose | NCT04963920, NCT04013269 |
|  | Continuous Renal Replacement Therapy | 1 | Recruiting | 460 | ICU | No |  | CRRT | Sepsis-3 | Patients with AKI | Mortality |  | NCT03175328 |
|  | Hemoabsorption | 1 | Recruiting | 40 | ICU | No |  | Hemoadsorp-tion | Sepsis-3, shock | Patients with AKI | Mortality, days alive without vasoactives, IMV and RRT |  | NCT04997421 |
|  | Hemodiafiltration | 1 | Recruiting | 28 | Not specified | No |  | Hemodiafiltration | Not specified | Patients with AKI |  | CRP reduction | NCT06227104 |
|  | Hemoperfusion | 2 | Recruiting | 150 | ICU or not specified | No |  | Triple-effect blood purification filter, HA-330 hemoperfusion filter hemodialysis vs. conventional hemodialysis | Sepsis-3 or not specified | Abdominal sepsis or patients with AKI | Duration of vasopressors | IL-1ra, IL-6, IL-10, TNF- α | NCT04957316, NCT05941624 |
|  | Immune cell perfusion system | 1 | Recruiting | 142 | ICU | No |  | Extracorporal treatment with purified granulocyte concentrate | Sepsis-3, shock | General | Safety and tolerability |  | NCT05442710 |
|  | NucleoCapture device | 1 | Not yet recruiting | 73 | ICU | No |  | NucleoCapture selective DNA adsorber | Not specified | General |  | Amount of cfDNA/NETs across the NucleoCapture column | NCT05647096 |
|  | Polymyxin B hemoperfu-sion | 2 | Recruiting | 170 | Not specified | Yes, both trials | 1) PCT > 2ng/mL, CRP > 150mg/L and EAA >0.6, 2) EAA ≥ 0.60 to <0.90 | Hemoperfu-sion with polymyxin B | Not specified | Cardiac surgery patients or general | Vasopressors, mortality | Endotoxin activity level | NCT04920565, NCT03901807 |
|  | Therapeutic Plasma Exchange | 1 | Not yet recruiting | 274 | ICU | No |  | Therapeutic Plasma Exchange | Sepsis-3, shock | General | Mortality |  | NCT05726825 |
| **Coagulation and endothelial dysfunction** | Recombinant Human Thrombopo-ietin | 1 | Recruiting | 200 | ICU | Yes | Platelets < 50×10^9/L | Recombinant Human Thrombopo-ietin | Sepsis-3 | General | Mortality |  | NCT02707497 |
|  | Tranexamic acid | 1 | Recruiting | 80 | ICU | No |  | Tranexamic acid | Not specified | General | Mortality |  | NCT04910464 |
|  | Unfractioned heparin | 2 | Recruiting | 700 | ICU | No |  | Unfractioned heparin | Sepsis-3 or not specified | Gram-Negative Bacterial sepsis or suspected DIC | Mortality |  | NCT04861922, NCT02654561 |
| **Pleiotropic drugs** | Corticostero-ids | 4 | Recruiting or not yet recruiting | 2312 | ICU, ward or not specified | Yes, one trial (NCT04280497) | Biomarker-guided, adaptive Bayesian design | Low vs. high dose dexamethasone, fludrocortisone and/or hydrocorti-sone | Sepsis-3 or not specified | General or CAP | Mortality, persistant organ dysfunction, composite adverse events |  | NCT05136560, NCT04280497, NCT06217939, NCT05818826 |
|  | Vitamin C | 2 | Recruiting | 182 | ICU | Yes, one trial [NCT05194189] | PCT ≥2 ng/ml | High-dose vitamin C | Sepsis-3 or sepsis-3, shock | General | Mortality, time to cessation of vasopressor |  | NCT05194189, NCT04796636 |
|  | Vitamin C and hydrocorti-sone and thiamine | 3 | Recruiting | 632 | ICU | No |  | Vitamin C and hydrocortisone and thiamine | Not specified or sepsis-3 | General or necrotizing soft tissue infection | Mortality, cerebral autoregula-tion and cerebral blood flow |  | NCT03649633, NCT03592693, NCT05157360 |
| **Immunonu-trition** | Alpha-Lipoic Acid group | 1 | Recruiting | 60 | ICU | No |  | Alpha-Lipoic Acid | Sepsis-3 | General | Mortality |  | NCT05808946 |
|  | L-citrulline | 1 | Recruiting | 130 | ICU | No |  | L-citrulline | Sepsis-3 | General | SOFA score |  | NCT04513288 |
|  | Lipid emulsion | 1 | Recruiting | 68 | ICU | Yes | Total cholesterol ≤ 100 mg/dL or HDL-C + LDL-C ≤ 70 mg/dL | Smoflipid | Sepsis-3 | General | Maximun tolerated dose | Total cholesterol | NCT03405870 |
|  | Kukoamine B | 1 | Recruiting | 424 | ICU | No |  | Kukoamine B | Sepsis-3 | Bacterial sepsis | SOFA score |  | NCT04803955 |
|  | Para-Tyrosine | 1 | Not yet recruiting | 296 | ICU | No |  | Para-Tyrosine supplementation | Sepsis-3 | General | Mortality |  | NCT03278730 |
|  | Senolytic therapy | 1 | Recruiting | 220 | Ward | No |  | Fisetin | Sepsis-3 | General | CRR-SOFA score |  |  |
|  | Vitamin D Receptor Activation | 1 | Not yet recruiting | 90 | ICU | No |  | Paricalcitol | Sepsis-3 | General | Mortality |  | NCT06209268 |
|  | Xanthohumol | 1 | Recruiting | 50 | Not specified | Yes | PCT >5 ng/ml and IL-6 > 100 pg/ml | Xanthohumol | Not specified | General | Mortality, length of stay | Inflammatory cytokines, glycocalyx damage | NCT06225258 |
|  | Zinc | 1 | Active, not recruiting | 30 | ICU | No |  | Zinc | Not specified | General |  | Pharmacoki-netics/phar-macodyna-mics | NCT01162109 |
| **Concomitant treatments** | Albumin | 1 | Not yet recruiting | 100 | ICU | Yes | Albumin plasma level <35g/L and lymphocytes count < 1,100 cel/mL | Human albumin | Sepsis-3, shock | CAP, urinary, skin or biliary infection | B cell response |  | NCT05645887 |
|  | β-antagonist | 2 | Active, not recruiting or recruiting | 89 | ICU. Not specified | No |  | Esmolol, landiolol | Sepsis-3, shock | General | Hymodyna-mic parameters | Immunomo-dulatory effect | NCT02120404, NCT04931225 |
|  | Dexmedetomidine | 1 | Recruiting | 116 | ICU | No |  | Dexmedetomidine | Sepsis-3 | General | Delirium |  | NCT04876937 |
|  | Dobutamine | 1 | Recruiting | 270 | ICU | No |  | Dobutamine | Sepsis-3 | Septic cardiomyopathy | SOFA score |  | NCT04166331 |
|  | Esketamine | 2 | Recruiting | 224 | ICU | No |  | Esketamine | Sepsis-3 or not specified | General | ICU-length of stay, doses of remifentanil and norepinephrine | Inflammatory cytokines, lymphocyte subsets | NCT04843982, NCT05839366 |
|  | Ondansetron | 1 | Recruiting | 40 | Not specified | No |  | Ondansetron | Sepsis-3 | General | SOFA score |  | NCT05402553 |
|  | Sevoflurane | 1 | Not yet recruiting | 153 | ICU | No |  | Sevoflurane | Not specified | General |  | IL-6 | NCT03643367 |
|  | Vasopressors | 1 | Not yet recruiting | 45 | Not specified | No |  | Angiotensin II + norepinephrine vs. vasopressin + norepineprhine vs. norepinephrine | Sepsis-3 | General | Renal cortical tissue blood flow |  | NCT06234592 |
| **Traditional Chinese Medicine** | Electroacupuncture | 1 | Recruiting | 40 | Not specified | No |  | Electroacupuncture | Sepsis-3 | ARDS |  | Lipoxin A4 levels | NCT06006325 |
|  | Shenfu Injection | 1 | Recruiting | 40 | Not specified | No |  | Shenfu Injection | Sepsis-3 | General | Henodynamic parameters, proportion of perfused vessels, microvascular flow index |  | NCT06157320 |
|  | YiQiFuMai Injection | 1 | Not yet recruiting | 80 | Not specified | No |  | YiQiFuMai Injection | Sepsis-3 | General | Mortality, secondary infection, length of stay | Hematologic and inflammatory markers | NCT05265130 |
| **Immunostimulatory**  **cytokines & growth**  **factors** | Interferon γ | 1 | Recruiting | 132 | ICU | Yes | HLA-DR < 8000 antibody/cell | Interferon γ | Not speficied | Ventilator-associated pneumonia | Ventilation-free days |  | NCT05843786 |
| **Intravenous**  **immunoglobulins** | Pentaglobin | 1 | Recruiting | 200 | Not specified | Yes | IL-6 is ≥ 1000 pg / ml | Pentaglobin | Not speficied | Peritonitis | Multiple Organ Failure |  | NCT03334006 |
| **Mesenchymal stem cells** | Mesenchymal stromal cells | 1 | Not yet recruiting | 296 | ICU | No |  | Umbilical cord-derived human mesenchymal stromal cells | Sepsis-3 | General | Days free from mechanical ventilation and/or vasopressors and/or renal replacement therapy |  | NCT05969275 |
| **Other/combi-nation of therapies** | Alirocumab | 1 | Recruiting | 50 | ICU | No |  | Alirocumab | Not specified | General |  | Bacterial endotoxin level, lipoteichoic acid level | NCT05469347 |
|  | Anakinra or Interferon-γ | 1 | Active, not recruiting | 280 | Not specified | Yes | Patients with hyperinflammation receive anakinra, patient with immunopa-ralysis interferon-gamma | Anakinra or Interferon-γ | Sepsis-3 | Pneumonia or blood stream infection | SOFA score |  | NCT04990232 |
|  | Apigenin | 1 | Not yet recruiting | 20 | ICU | No |  | Apigenin | Sepsis-3 | General | SOFA score |  | NCT05999682 |
|  | Epirubicin | 1 | Recruiting | 45 | ICU | No |  | Epirubicin | Not specified | General | Myelotoxicity |  | NCT05033808 |
|  | External Cooling | 1 | Recruiting | 820 | ICU | No |  | External Cooling | Sepsis-3, shock | General | Mortality |  | NCT04494074 |
|  | Metformin | 2 | Not yet recruiting or recruiting | 185 | Not specified | No |  | Metformin | Not specified or sepsis-3 | Diabetic and non-dianbetic or general | Mortality |  | NCT05572060, NCT05979038 |
|  | Metformin and lactoferrin | 1 | Not yet recruiting | 60 | ICU | No |  | Metformin vs. lactoferrin | Sepsis-3 | General | Mortality |  | NCT06181422 |
|  | Paracetamol and Ibuprofen | 1 | Recruitng | 84 | Not specified | No |  | Paracetamol vs. ibuprofen | Sepsis-3 | General | Fever |  | NCT06061575 |
|  | SSRI | 1 | Recruiting | 20 | ICU | No |  | Paroxetine | Not specified | General | Cardiovascu-lair SOFA score |  | NCT05725837 |
|  | Vagus Nerve Stimulation | 1 | Recruiting | 34 | Not specified | No |  | Stimulation of the vagus nerve | Not specified | General |  | TNF- α | NCT03992378 |

Abbreviations: AKI, acute kidney injury; ARDS, acute respiratory stress syndrome; CAP, community-acquired pneumonia; COVID-19, coronavirus disease of 2019; CRP, C-reactive protein; CRR, cardiovascular, respiratory, and renal; (C)RRT, (continuous) renal replacement therapy; DIC, disseminated intravascular coagulation; EAA, endotoxin activity assay; ECMO, extracorporeal membrane oxygenation; ED, emergency department; ICU, intensive care unit; IL(ra), interleukin (receptor antagonist); IMV, invasive mechanical ventilation; NCT, National Clinical Trial; PCT, procalcitonin; RCT, randomized controlled trial; RRT, renal replacement therapy; SOFA, sequential organ failure assessment; SSRI, selective serotonin reuptake inhibitors; TNF- α, tumor necrosis factor- α; UTI, urinary tract infection; VT, ventricular tachycardia.

**References**

1. Arksey H, O'Malley L. Scoping studies: towards a methodological framework. International Journal of Social Research Methodology. 2005;8(1):19-32.

2. Wohlin C, editor Guidelines for snowballing in systematic literature studies and a replication in software engineering. Proceedings of the 18th international conference on evaluation and assessment in software engineering; 2014.

3. Beale RJ, Sherry T, Lei K, Campbell-Stephen L, McCook J, Smith J, et al. Early enteral supplementation with key pharmaconutrients improves Sequential Organ Failure Assessment score in critically ill patients with sepsis: outcome of a randomized, controlled, double-blind trial. Crit Care Med. 2008;36(1):131-44.

4. Barbosa VM, Miles EA, Calhau C, Lafuente E, Calder PC. Effects of a fish oil containing lipid emulsion on plasma phospholipid fatty acids, inflammatory markers, and clinical outcomes in septic patients: a randomized, controlled clinical trial. Crit Care. 2010;14(1):R5.

5. Hall TC, Bilku DK, Al-Leswas D, Neal CP, Horst C, Cooke J, et al. A randomized controlled trial investigating the effects of parenteral fish oil on survival outcomes in critically ill patients with sepsis: a pilot study. JPEN J Parenter Enteral Nutr. 2015;39(3):301-12.

6. Bloos F, Trips E, Nierhaus A, Briegel J, Heyland DK, Jaschinski U, et al. Effect of Sodium Selenite Administration and Procalcitonin-Guided Therapy on Mortality in Patients With Severe Sepsis or Septic Shock: A Randomized Clinical Trial. JAMA Intern Med. 2016;176(9):1266-76.

7. Avontuur JA, Stam TC, Jongen-Lavrencic M, van Amsterdam JG, Eggermont AM, Bruining HA. Effect of L-NAME, an inhibitor of nitric oxide synthesis, on plasma levels of IL-6, IL-8, TNF alpha and nitrite/nitrate in human septic shock. Intensive Care Med. 1998;24(7):673-9.

8. Ohta Y, Miyamoto K, Kawazoe Y, Yamamura H, Morimoto T. Effect of dexmedetomidine on inflammation in patients with sepsis requiring mechanical ventilation: a sub-analysis of a multicenter randomized clinical trial. Crit Care. 2020;24(1):493.

9. Antcliffe DB, Santhakumaran S, Orme RML, Ward JK, Al-Beidh F, O'Dea K, et al. Levosimendan in septic shock in patients with biochemical evidence of cardiac dysfunction: a subgroup analysis of the LeoPARDS randomised trial. Intensive Care Med. 2019;45(10):1392-400.

10. Zhang N, Liu J, Qiu Z, Ye Y, Zhang J, Lou T. Shenfu injection for improving cellular immunity and clinical outcome in patients with sepsis or septic shock. Am J Emerg Med. 2017;35(1):1-6.

11. Liu S, Yao C, Xie J, Liu H, Wang H, Lin Z, et al. Effect of an Herbal-Based Injection on 28-Day Mortality in Patients With Sepsis: The EXIT-SEP Randomized Clinical Trial. JAMA Internal Medicine. 2023;183(7):647-55.

12. Zhang Y, Chen H, Li YM, Zheng SS, Chen YG, Li LJ, et al. Thymosin alpha1- and ulinastatin-based immunomodulatory strategy for sepsis arising from intra-abdominal infection due to carbapenem-resistant bacteria. J Infect Dis. 2008;198(5):723-30.

13. Casarotta E, Damiani E, Domizi R, Carsetti A, Scorcella C, Adrario E, et al. Variation in the Outcome of Norepinephrine-Dependent Septic Patients After the Institution of a Patient-Tailored Therapy Protocol in an Italian Intensive Care Unit: Retrospective Observational Study. Front Med (Lausanne). 2020;7:592282.

14. Leventogiannis K, Kyriazopoulou E, Antonakos N, Kotsaki A, Tsangaris I, Markopoulou D, et al. Toward personalized immunotherapy in sepsis: The PROVIDE randomized clinical trial. Cell Rep Med. 2022;3(11):100817.

15. Pickkers P, Mehta RL, Murray PT, Joannidis M, Molitoris BA, Kellum JA, et al. Effect of Human Recombinant Alkaline Phosphatase on 7-Day Creatinine Clearance in Patients With Sepsis-Associated Acute Kidney Injury: A Randomized Clinical Trial. Jama. 2018;320(19):1998-2009.

16. The French National Registry of HA-1A (Centoxin) in septic shock. A cohort study of 600 patients. The National Committee for the Evaluation of Centoxin. Arch Intern Med. 1994;154(21):2484-91.

17. Bates DW, Lee TH. Projected impact of monoclonal anti-endotoxin antibody therapy. Arch Intern Med. 1994;154(11):1241-9.

18. Chalfin DB, Holbein ME, Fein AM, Carlon GC. Cost-effectiveness of monoclonal antibodies to gram-negative endotoxin in the treatment of gram-negative sepsis in ICU patients. Jama. 1993;269(2):249-54.

19. Kett DH, Quartin AA, Sprung CL, Fisher CJ, Jr., Peña MA, Heard SO, et al. An evaluation of the hemodynamic effects of HA-1A human monoclonal antibody. Crit Care Med. 1994;22(8):1227-34.

20. Knaus WA, Harrell FE, Jr., LaBrecque JF, Wagner DP, Pribble JP, Draper EA, et al. Use of predicted risk of mortality to evaluate the efficacy of anticytokine therapy in sepsis. The rhIL-1ra Phase III Sepsis Syndrome Study Group. Crit Care Med. 1996;24(1):46-56.

21. Meyer NJ, Reilly JP, Anderson BJ, Palakshappa JA, Jones TK, Dunn TG, et al. Mortality Benefit of Recombinant Human Interleukin-1 Receptor Antagonist for Sepsis Varies by Initial Interleukin-1 Receptor Antagonist Plasma Concentration. Crit Care Med. 2018;46(1):21-8.

22. Shakoory B, Carcillo JA, Chatham WW, Amdur RL, Zhao H, Dinarello CA, et al. Interleukin-1 Receptor Blockade Is Associated With Reduced Mortality in Sepsis Patients With Features of Macrophage Activation Syndrome: Reanalysis of a Prior Phase III Trial. Crit Care Med. 2016;44(2):275-81.

23. Vincent JL, Slotman G, Van Leeuwen PA, Shelly M, Nasraway S, Tenaillon A, et al. IL-1ra administration does not improve cardiac function in patients with severe sepsis. J Crit Care. 1999;14(2):69-72.

24. Hamilton FW, Thomas M, Arnold D, Palmer T, Moran E, Mentzer AJ, et al. Therapeutic potential of IL6R blockade for the treatment of sepsis and sepsis-related death: A Mendelian randomisation study. PLoS Med. 2023;20(1):e1004174.

25. Chen S, Li S, Kuang C, Zhong Y, Yang Z, Yang Y, Liu F. Aspirin reduces the mortality risk of sepsis-associated acute kidney injury: an observational study using the MIMIC IV database. Front Pharmacol. 2023;14:1186384.

26. Dong R, Zhang X, Zhao Z. Ulinastatin as an Adjuvant Therapy to Restricting Volumes of Resuscitation Fluid Strategy for Patients with Septic Shock after Initial Management. Evid Based Complement Alternat Med. 2021;2021:4231454.

27. Boekstegers P, Weidenhöfer S, Zell R, Holler E, Kapsner T, Redl H, et al. Changes in skeletal muscle pO2 after administration of anti-TNF alpha-antibody in patients with severe sepsis: comparison to interleukin-6 serum levels, APACHE II, and Elebute scores. Shock. 1994;1(4):246-53.

28. Boekstegers P, Weidenhöfer S, Zell R, Pilz G, Holler E, Ertel W, et al. Repeated administration of a F(ab')2 fragment of an anti-tumor necrosis factor alpha monoclonal antibody in patients with severe sepsis: effects on the cardiovascular system and cytokine levels. Shock. 1994;1(4):237-45.

29. Boillot A, Capellier G, Racadot E, Wijdenes J, Herve P, Barale F. Pilot clinical trial of an anti-TNF alpha monoclonal antibody for the treatment of septic shock. Clin Intensive Care. 1995;6(2):52-6.

30. Fisher CJ, Jr., Opal SM, Dhainaut JF, Stephens S, Zimmerman JL, Nightingale P, et al. Influence of an anti-tumor necrosis factor monoclonal antibody on cytokine levels in patients with sepsis. The CB0006 Sepsis Syndrome Study Group. Crit Care Med. 1993;21(3):318-27.

31. van Heerden PV, Abutbul A, Sviri S, Zlotnick E, Nama A, Zimro S, et al. Apoptotic Cells for Therapeutic Use in Cytokine Storm Associated With Sepsis- A Phase Ib Clinical Trial. Front Immunol. 2021;12:718191.

32. Aitchison JM, Arbuckle DD. Anti-endotoxin in the treatment of severe surgical septic shock. Results of a randomized double-blind trial. S Afr Med J. 1985;68(11):787-9.

33. Bigatello LM, Greene RE, Sprung CL, Panacek EA, Straube RC, Zimmerman JL, et al. HA-1A in septic patients with ARDS: results from the pivotal trial. Intensive Care Med. 1994;20(5):328-34.

34. Bone RC, Balk RA, Fein AM, Perl TM, Wenzel RP, Reines HD, et al. A second large controlled clinical study of E5, a monoclonal antibody to endotoxin: results of a prospective, multicenter, randomized, controlled trial. The E5 Sepsis Study Group. Crit Care Med. 1995;23(6):994-1006.

35. Greenberg RN, Wilson KM, Kunz AY, Wedel NI, Gorelick KJ. Observations using antiendotoxin antibody (E5) as adjuvant therapy in humans with suspected, serious, gram-negative sepsis. Crit Care Med. 1992;20(6):730-5.

36. Lachman E, Pitsoe SB, Gaffin SL. Anti-lipopolysaccharide immunotherapy in management of septic shock of obstetric and gynaecological origin. Lancet. 1984;1(8384):981-3.

37. McCloskey RV, Straube RC, Sanders C, Smith SM, Smith CR. Treatment of septic shock with human monoclonal antibody HA-1A. A randomized, double-blind, placebo-controlled trial. CHESS Trial Study Group. Ann Intern Med. 1994;121(1):1-5.

38. Wortel CH, von der Möhlen MA, van Deventer SJ, Sprung CL, Jastremski M, Lubbers MJ, et al. Effectiveness of a human monoclonal anti-endotoxin antibody (HA-1A) in gram-negative sepsis: relationship to endotoxin and cytokine levels. J Infect Dis. 1992;166(6):1367-74.

39. Ziegler E, McCutchan J, Douglas H, Braude A. Successful treatment of human gram-negative bacteremia with antiserum against endotoxin core. Transactions of the Association of American physicians. 1981;94:39-43.

40. Ziegler EJ, McCutchan JA, Fierer J, Glauser MP, Sadoff JC, Douglas H, Braude AI. Treatment of gram-negative bacteremia and shock with human antiserum to a mutant Escherichia coli. N Engl J Med. 1982;307(20):1225-30.

41. Ziegler EJ, Fisher CJ, Jr., Sprung CL, Straube RC, Sadoff JC, Foulke GE, et al. Treatment of gram-negative bacteremia and septic shock with HA-1A human monoclonal antibody against endotoxin. A randomized, double-blind, placebo-controlled trial. The HA-1A Sepsis Study Group. N Engl J Med. 1991;324(7):429-36.

42. Abraham E, Anzueto A, Gutierrez G, Tessler S, San Pedro G, Wunderink R, et al. Double-blind randomised controlled trial of monoclonal antibody to human tumour necrosis factor in treatment of septic shock. NORASEPT II Study Group. Lancet. 1998;351(9107):929-33.

43. Abraham E, Wunderink R, Silverman H, Perl TM, Nasraway S, Levy H, et al. Efficacy and safety of monoclonal antibody to human tumor necrosis factor alpha in patients with sepsis syndrome. A randomized, controlled, double-blind, multicenter clinical trial. TNF-alpha MAb Sepsis Study Group. Jama. 1995;273(12):934-41.

44. Aikawa N, Takahashi T, Fujimi S, Yokoyama T, Yoshihara K, Ikeda T, et al. A Phase II study of polyclonal anti-TNF-α (AZD9773) in Japanese patients with severe sepsis and/or septic shock. J Infect Chemother. 2013;19(5):931-40.

45. Bernard GR, Francois B, Mira JP, Vincent JL, Dellinger RP, Russell JA, et al. Evaluating the efficacy and safety of two doses of the polyclonal anti-tumor necrosis factor-α fragment antibody AZD9773 in adult patients with severe sepsis and/or septic shock: randomized, double-blind, placebo-controlled phase IIb study*. Crit Care Med. 2014;42(3):504-11.

46. Clark MA, Plank LD, Connolly AB, Streat SJ, Hill AA, Gupta R, et al. Effect of a chimeric antibody to tumor necrosis factor-alpha on cytokine and physiologic responses in patients with severe sepsis--a randomized, clinical trial. Crit Care Med. 1998;26(10):1650-9.

47. Cohen J, Carlet J. INTERSEPT: an international, multicenter, placebo-controlled trial of monoclonal antibody to human tumor necrosis factor-alpha in patients with sepsis. International Sepsis Trial Study Group. Crit Care Med. 1996;24(9):1431-40.

48. Dhainaut JF, Vincent JL, Richard C, Lejeune P, Martin C, Fierobe L, et al. CDP571, a humanized antibody to human tumor necrosis factor-alpha: safety, pharmacokinetics, immune response, and influence of the antibody on cytokine concentrations in patients with septic shock. CPD571 Sepsis Study Group. Crit Care Med. 1995;23(9):1461-9.

49. Gallagher J, Fisher C, Sherman B, Munger M, Meyers B, Ellison T, et al. A multicenter, open-label, prospective, randomized, dose-ranging pharmacokinetic study of the anti-TNF-alpha antibody afelimomab in patients with sepsis syndrome. Intensive Care Med. 2001;27(7):1169-78.

50. Morris PE, Zeno B, Bernard AC, Huang X, Das S, Edeki T, et al. A placebo-controlled, double-blind, dose-escalation study to assess the safety, tolerability and pharmacokinetics/pharmacodynamics of single and multiple intravenous infusions of AZD9773 in patients with severe sepsis and septic shock. Crit Care. 2012;16(1):R31.

51. Panacek EA, Marshall JC, Albertson TE, Johnson DH, Johnson S, MacArthur RD, et al. Efficacy and safety of the monoclonal anti-tumor necrosis factor antibody F(ab')2 fragment afelimomab in patients with severe sepsis and elevated interleukin-6 levels. Crit Care Med. 2004;32(11):2173-82.

52. Reinhart K, Menges T, Gardlund B, Harm Zwaveling J, Smithes M, Vincent JL, et al. Randomized, placebo-controlled trial of the anti-tumor necrosis factor antibody fragment afelimomab in hyperinflammatory response during severe sepsis: The RAMSES Study. Crit Care Med. 2001;29(4):765-9.

53. Reinhart K, Wiegand-Löhnert C, Grimminger F, Kaul M, Withington S, Treacher D, et al. Assessment of the safety and efficacy of the monoclonal anti-tumor necrosis factor antibody-fragment, MAK 195F, in patients with sepsis and septic shock: a multicenter, randomized, placebo-controlled, dose-ranging study. Crit Care Med. 1996;24(5):733-42.

54. Rice TW, Wheeler AP, Morris PE, Paz HL, Russell JA, Edens TR, Bernard GR. Safety and efficacy of affinity-purified, anti-tumor necrosis factor-alpha, ovine fab for injection (CytoFab) in severe sepsis. Crit Care Med. 2006;34(9):2271-81.

55. Laterre PF, Colin G, Dequin PF, Dugernier T, Boulain T, Azeredo da Silveira S, et al. CAL02, a novel antitoxin liposomal agent, in severe pneumococcal pneumonia: a first-in-human, double-blind, placebo-controlled, randomised trial. Lancet Infect Dis. 2019;19(6):620-30.

56. Reinhart K, Glück T, Ligtenberg J, Tschaikowsky K, Bruining A, Bakker J, et al. CD14 receptor occupancy in severe sepsis: results of a phase I clinical trial with a recombinant chimeric CD14 monoclonal antibody (IC14). Crit Care Med. 2004;32(5):1100-8.

57. Fein AM, Bernard GR, Criner GJ, Fletcher EC, Good JT, Jr., Knaus WA, et al. Treatment of severe systemic inflammatory response syndrome and sepsis with a novel bradykinin antagonist, deltibant (CP-0127). Results of a randomized, double-blind, placebo-controlled trial. CP-0127 SIRS and Sepsis Study Group. Jama. 1997;277(6):482-7.

58. Donnino MW, Mortensen SJ, Andersen LW, Chase M, Berg KM, Balkema J, et al. Ubiquinol (reduced Coenzyme Q10) in patients with severe sepsis or septic shock: a randomized, double-blind, placebo-controlled, pilot trial. Crit Care. 2015;19(1):275.

59. Soltani R, Alikiaie B, Shafiee F, Amiri H, Mousavi S. Coenzyme Q10 improves the survival and reduces inflammatory markers in septic patients. Bratisl Lek Listy. 2020;121(2):154-8.

60. Wan B, Zhang H, Fu H, Chen Y, Yang L, Yin J, et al. Recombinant human interleukin-11 (IL-11) is a protective factor in severe sepsis with thrombocytopenia: A case-control study. Cytokine. 2015;76(2):138-43.

61. Boermeester MA, van Leeuwen PA, Coyle SM, Wolbink GJ, Hack CE, Lowry SF. Interleukin-1 blockade attenuates mediator release and dysregulation of the hemostatic mechanism during human sepsis. Arch Surg. 1995;130(7):739-48.

62. Fisher CJ, Jr., Dhainaut JF, Opal SM, Pribble JP, Balk RA, Slotman GJ, et al. Recombinant human interleukin 1 receptor antagonist in the treatment of patients with sepsis syndrome. Results from a randomized, double-blind, placebo-controlled trial. Phase III rhIL-1ra Sepsis Syndrome Study Group. Jama. 1994;271(23):1836-43.

63. Fisher CJ, Jr., Slotman GJ, Opal SM, Pribble JP, Bone RC, Emmanuel G, et al. Initial evaluation of human recombinant interleukin-1 receptor antagonist in the treatment of sepsis syndrome: a randomized, open-label, placebo-controlled multicenter trial. Crit Care Med. 1994;22(1):12-21.

64. Opal SM, Fisher CJ, Jr., Dhainaut JF, Vincent JL, Brase R, Lowry SF, et al. Confirmatory interleukin-1 receptor antagonist trial in severe sepsis: a phase III, randomized, double-blind, placebo-controlled, multicenter trial. The Interleukin-1 Receptor Antagonist Sepsis Investigator Group. Crit Care Med. 1997;25(7):1115-24.

65. Slotman GJ, Friedman B, Brathwaite C, Mure AJ, Quinn JV, Shapiro E. Interleukin-1 mediates increased plasma levels of eicosanoids and cytokines in patients with sepsis syndrome. Shock. 1995;4(5):318-23.

66. Slotman GJ, Quinn JV, Wry PC, Brathwaite CE, Friedman BM. Unopposed interleukin-1 is necessary for increased plasma cytokine and eicosanoid levels to develop in severe sepsis. Ann Surg. 1997;226(1):77-84.

67. Sehgal IS, Agarwal R, Aggarwal AN, Jindal SK. A randomized trial of Mycobacterium w in severe sepsis. J Crit Care. 2015;30(1):85-9.

68. Sehgal IS, Basumatary NM, Dhooria S, Prasad KT, Muthu V, Aggarwal AN, et al. A Randomized Trial of Mycobacterium w in Severe Presumed Gram-Negative Sepsis. Chest. 2021;160(4):1282-91.

69. François B, Wittebole X, Ferrer R, Mira JP, Dugernier T, Gibot S, et al. Nangibotide in patients with septic shock: a Phase 2a randomized controlled clinical trial. Intensive Care Med. 2020;46(7):1425-37.

70. François B, Lambden S, Fivez T, Gibot S, Derive M, Grouin JM, et al. Prospective evaluation of the efficacy, safety, and optimal biomarker enrichment strategy for nangibotide, a TREM-1 inhibitor, in patients with septic shock (ASTONISH): a double-blind, randomised, controlled, phase 2b trial. Lancet Respir Med. 2023;11(10):894-904.

71. Arons MM, Wheeler AP, Bernard GR, Christman BW, Russell JA, Schein R, et al. Effects of ibuprofen on the physiology and survival of hypothermic sepsis. Ibuprofen in Sepsis Study Group. Crit Care Med. 1999;27(4):699-707.

72. Bernard GR, Reines HD, Halushka PV, Higgins SB, Metz CA, Swindell BB, et al. Prostacyclin and thromboxane A2 formation is increased in human sepsis syndrome. Effects of cyclooxygenase inhibition. Am Rev Respir Dis. 1991;144(5):1095-101.

73. Bernard GR, Wheeler AP, Russell JA, Schein R, Summer WR, Steinberg KP, et al. The effects of ibuprofen on the physiology and survival of patients with sepsis. The Ibuprofen in Sepsis Study Group. N Engl J Med. 1997;336(13):912-8.

74. Haupt MT, Jastremski MS, Clemmer TP, Metz CA, Goris GB. Effect of ibuprofen in patients with severe sepsis: a randomized, double-blind, multicenter study. The Ibuprofen Study Group. Crit Care Med. 1991;19(11):1339-47.

75. Memiş D, Karamanlioğlu B, Turan A, Koyuncu O, Pamukçu Z. Effects of lornoxicam on the physiology of severe sepsis. Crit Care. 2004;8(6):R474-82.

76. Abraham E, Glauser MP, Butler T, Garbino J, Gelmont D, Laterre PF, et al. p55 Tumor necrosis factor receptor fusion protein in the treatment of patients with severe sepsis and septic shock. A randomized controlled multicenter trial. Ro 45-2081 Study Group. Jama. 1997;277(19):1531-8.

77. Abraham E, Laterre PF, Garbino J, Pingleton S, Butler T, Dugernier T, et al. Lenercept (p55 tumor necrosis factor receptor fusion protein) in severe sepsis and early septic shock: a randomized, double-blind, placebo-controlled, multicenter phase III trial with 1,342 patients. Crit Care Med. 2001;29(3):503-10.

78. Pittet D, Harbarth S, Suter PM, Reinhart K, Leighton A, Barker C, et al. Impact of immunomodulating therapy on morbidity in patients with severe sepsis. Am J Respir Crit Care Med. 1999;160(3):852-7.

79. Dellinger RP, Tomayko JF, Angus DC, Opal S, Cupo MA, McDermott S, et al. Efficacy and safety of a phospholipid emulsion (GR270773) in Gram-negative severe sepsis: results of a phase II multicenter, randomized, placebo-controlled, dose-finding clinical trial. Crit Care Med. 2009;37(11):2929-38.

80. Chávez-Iñiguez JS, Poo JL, Ibarra-Estrada M, García-Benavides L, Navarro-Blackaller G, Cervantes-Sánchez C, et al. Effect of Prolonged-Release Pirfenidone on Renal Function in Septic Acute Kidney Injury Patients: A Double-Blind Placebo-Controlled Clinical Trial. Int J Nephrol. 2021;2021:8833278.

81. Willatts SM, Radford S, Leitermann M. Effect of the antiendotoxic agent, taurolidine, in the treatment of sepsis syndrome: a placebo-controlled, double-blind trial. Crit Care Med. 1995;23(6):1033-9.

82. Kalil AC, LaRosa SP, Gogate J, Lynn M, Opal SM. Influence of severity of illness on the effects of eritoran tetrasodium (E5564) and on other therapies for severe sepsis. Shock. 2011;36(4):327-31.

83. Opal SM, Laterre PF, Francois B, LaRosa SP, Angus DC, Mira JP, et al. Effect of eritoran, an antagonist of MD2-TLR4, on mortality in patients with severe sepsis: the ACCESS randomized trial. Jama. 2013;309(11):1154-62.

84. Rice TW, Wheeler AP, Bernard GR, Vincent JL, Angus DC, Aikawa N, et al. A randomized, double-blind, placebo-controlled trial of TAK-242 for the treatment of severe sepsis. Crit Care Med. 2010;38(8):1685-94.

85. Tidswell M, Tillis W, Larosa SP, Lynn M, Wittek AE, Kao R, et al. Phase 2 trial of eritoran tetrasodium (E5564), a toll-like receptor 4 antagonist, in patients with severe sepsis. Crit Care Med. 2010;38(1):72-83.

86. Fisher CJ, Jr., Agosti JM, Opal SM, Lowry SF, Balk RA, Sadoff JC, et al. Treatment of septic shock with the tumor necrosis factor receptor:Fc fusion protein. The Soluble TNF Receptor Sepsis Study Group. N Engl J Med. 1996;334(26):1697-702.

87. Zhou S, Gao H, Chen J, Liu W. The Protective Effect of Ulinastatin in Severe Sepsis. A Mechanistic Approach. Farmacia. 2020;68(1):121-8.

88. Guo M, Zhou B. Clinical efficacy of ulinastatin in the treatment of unliquefied pyogenic liver abscess complicated by septic shock: A randomized controlled trial. Immun Inflamm Dis. 2023;11(4):e822.

89. Zuccari S, Damiani E, Domizi R, Scorcella C, D'Arezzo M, Carsetti A, et al. Changes in Cytokines, Haemodynamics and Microcirculation in Patients with Sepsis/Septic Shock Undergoing Continuous Renal Replacement Therapy and Blood Purification with CytoSorb. Blood Purif. 2020;49(1-2):107-13.

90. Brouwer WP, Duran S, Kuijper M, Ince C. Hemoadsorption with CytoSorb shows a decreased observed versus expected 28-day all-cause mortality in ICU patients with septic shock: a propensity-score-weighted retrospective study. Crit Care. 2019;23(1):317.

91. Kaya Uğur B, Çiçek H, Kul S, Mete Ö, Yılmaz M. Effect of a novel extracorporeal cytokine apheresis method on endocan, copeptin And interleukin-6 levels in sepsis: An observational prospective study. Transfus Apher Sci. 2020;59(6):102919.

92. Mehta Y, Mehta C, Kumar A, George JV, Gupta A, Nanda S, et al. Experience with hemoadsorption (CytoSorb(®)) in the management of septic shock patients. World J Crit Care Med. 2020;9(1):1-12.

93. Paul R, Sathe P, Kumar S, Prasad S, Aleem M, Sakhalvalkar P. Multicentered prospective investigator initiated study to evaluate the clinical outcomes with extracorporeal cytokine adsorption device (CytoSorb(®)) in patients with sepsis and septic shock. World J Crit Care Med. 2021;10(1):22-34.

94. Akil A, Ziegeler S, Reichelt J, Rehers S, Abdalla O, Semik M, Fischer S. Combined Use of CytoSorb and ECMO in Patients with Severe Pneumogenic Sepsis. Thorac Cardiovasc Surg. 2021;69(3):246-51.

95. Shum HP, Chan KC, Kwan MC, Yan WW. Application of endotoxin and cytokine adsorption haemofilter in septic acute kidney injury due to Gram-negative bacterial infection. Hong Kong Med J. 2013;19(6):491-7.

96. Naka T, Shinozaki M, Akizawa T, Shima Y, Takaesu H, Nasu H. The effect of continuous veno-venous hemofiltration or direct hemoperfusion with polymyxin B-immobilized fiber on neutrophil respiratory oxidative burst in patients with sepsis and septic shock. Ther Apher Dial. 2006;10(1):7-11.

97. Tojimbara T, Sato S, Nakajima I, Fuchinoue S, Akiba T, Teraoka S. Polymyxin B-immobilized fiber hemoperfusion after emergency surgery in patients with chronic renal failure. Ther Apher Dial. 2004;8(4):286-92.

98. Tsujimoto H, Ono S, Hiraki S, Majima T, Kawarabayashi N, Sugasawa H, et al. Hemoperfusion with polymyxin B-immobilized fibers reduced the number of CD16+ CD14+ monocytes in patients with septic shock. J Endotoxin Res. 2004;10(4):229-37.

99. Uriu K, Osajima A, Hiroshige K, Watanabe H, Aibara K, Inada Y, et al. Endotoxin removal by direct hemoperfusion with an adsorbent column using polymyxin B-immobilized fiber ameliorates systemic circulatory disturbance in patients with septic shock. Am J Kidney Dis. 2002;39(5):937-47.

100. Yaroustovsky M, Abramyan M, Komardina E, Nazarova H, Popov D, Plyushch M, et al. Selective LPS Adsorption Using Polymyxin B-Immobilized Fiber Cartridges in Sepsis Patients Following Cardiac Surgery. Shock. 2018;49(6):658-66.

101. Mitaka C, Kusao M, Kawagoe I, Satoh D, Iba T, Ronco C. Impact of Extended Duration of Polymyxin B-Immobilized Fiber Column Direct Hemoperfusion on Hemodynamics, Vasoactive Substance Requirement, and Pulmonary Oxygenation in Patients with Sepsis: An Observational Study. Blood Purif. 2022;51(1):62-9.

102. Lee WY, Kim HJ, Kim EY. Impact of polymyxin B hemoperfusion therapy on high endotoxin activity level patients after successful infection source control: a prospective cohort study. Sci Rep. 2021;11(1):24132.

103. Fujimori K, Tarasawa K, Fushimi K. Effectiveness of polymyxin B hemoperfusion for sepsis depends on the baseline SOFA score: a nationwide observational study. Ann Intensive Care. 2021;11(1):141.

104. Klein DJ, Foster D, Walker PM, Bagshaw SM, Mekonnen H, Antonelli M. Polymyxin B hemoperfusion in endotoxemic septic shock patients without extreme endotoxemia: a post hoc analysis of the EUPHRATES trial. Intensive Care Med. 2018;44(12):2205-12.

105. Cutuli SL, Artigas A, Fumagalli R, Monti G, Ranieri VM, Ronco C, Antonelli M. Polymyxin-B hemoperfusion in septic patients: analysis of a multicenter registry. Ann Intensive Care. 2016;6(1):77.

106. Fujimori K, Tarasawa K, Fushimi K. Effects of Polymyxin B Hemoperfusion on Septic Shock Patients Requiring Noradrenaline: Analysis of a Nationwide Administrative Database in Japan. Blood Purif. 2021;50(4-5):560-5.

107. Ikeda T, Ikeda K, Nagura M, Taniuchi H, Matsushita M, Kiuchi S, et al. Clinical evaluation of PMX-DHP for hypercytokinemia caused by septic multiple organ failure. Ther Apher Dial. 2004;8(4):293-8.

108. Iwagami M, Yasunaga H, Doi K, Horiguchi H, Fushimi K, Matsubara T, et al. Postoperative polymyxin B hemoperfusion and mortality in patients with abdominal septic shock: a propensity-matched analysis. Crit Care Med. 2014;42(5):1187-93.

109. Iwagami M, Yasunaga H, Noiri E, Horiguchi H, Fushimi K, Matsubara T, et al. Potential Survival Benefit of Polymyxin B Hemoperfusion in Septic Shock Patients on Continuous Renal Replacement Therapy: A Propensity-Matched Analysis. Blood Purif. 2016;42(1):9-17.

110. Kanesaka S, Sasaki J, Kuzume M, Narihara K, Takahashi Y. Effect of direct hemoperfusion using polymyxin B immobilized fiber on inflammatory mediators in patients with severe sepsis and septic shock. Int J Artif Organs. 2008;31(10):891-7.

111. Kawazoe Y, Sato T, Miyagawa N, Yokokawa Y, Kushimoto S, Miyamoto K, et al. Mortality Effects of Prolonged Hemoperfusion Therapy Using a Polymyxin B-Immobilized Fiber Column for Patients with Septic Shock: A Sub-Analysis of the DESIRE Trial. Blood Purif. 2018;46(4):309-14.

112. Kushi H, Miki T, Sakagami Y, Saito T, Tanjoh K. Criteria for direct hemoperfusion with an immobilized polymyxin B fiber column based on oxygen metabolism. Ther Apher Dial. 2008;12(4):292-7.

113. Lee CT, Tu YK, Yeh YC, Chang T, Shih PY, Chao A, et al. Effects of polymyxin B hemoperfusion on hemodynamics and prognosis in septic shock patients. J Crit Care. 2018;43:202-6.

114. Lee JM, Baek SD, Kim TH, Jeon HR, Han JH, Chang JW. Uncertain Clinical Effect of Polymyxin B Hemoperfusion in Patients with Septic Acute Kidney Injury Requiring Continuous Renal Replacement Therapy. Shock. 2021;56(4):551-6.

115. Miyamoto K, Kawazoe Y, Kato S. Prolonged direct hemoperfusion using a polymyxin B immobilized fiber cartridge provides sustained circulatory stabilization in patients with septic shock: a retrospective observational before-after study. Journal of Intensive Care. 2017;5(1):19.

116. Murakami M, Miyauchi Y, Nishida M, Okada H, Hamano K. Direct hemoperfusion using polymyxin-B immobilized fiber for septic shock after cardiac surgery. Circ J. 2009;73(4):658-61.

117. Nakamura T, Ebihara I, Shimada N, Koide H. Changes in plasma erythropoietin and interleukin-6 concentrations in patients with septic shock after hemoperfusion with polymyxin B-immobilized fiber. Intensive Care Med. 1998;24(12):1272-6.

118. Nakamura T, Ebihara I, Shimada N, Shoji H, Koide H. Modulation of plasma metalloproteinase-9 concentrations and peripheral blood monocyte mRNA levels in patients with septic shock: effect of fiber-immobilized polymyxin B treatment. Am J Med Sci. 1998;316(6):355-60.

119. Nakamura T, Ebihara I, Shimada N, Suzuki S, Ushiyama C, Shoji H, Koide H. Effects of hemoperfusion with polymyxin B-immobilized fibre on serum neopterin and soluble interleukin-2 receptor concentrations in patients with septic shock. J Infect. 1998;37(3):241-7.

120. Nakamura T, Fujiwara N, Sato E, Kawagoe Y, Ueda Y, Yamada S, Koide H. Effect of polymyxin B-immobilized fiber hemoperfusion on serum high mobility group box-1 protein levels and oxidative stress in patients with acute respiratory distress syndrome. Asaio j. 2009;55(4):395-9.

121. Nakamura T, Kawagoe Y, Matsuda T, Ebihara I, Koide H. Effects of polymyxin B-immobilized fiber hemoperfusion on amino acid imbalance in septic encephalopathy. Blood Purif. 2003;21(4-5):282-6.

122. Nakamura T, Kawagoe Y, Matsuda T, Koide H. Effect of polymyxin B-immobilized fiber on bone resorption in patients with sepsis. Intensive Care Med. 2004;30(9):1838-41.

123. Nakamura T, Kawagoe Y, Suzuki T, Shoji H, Ueda Y, Koide H. Polymyxin B-immobilized fiber hemoperfusion with the PMX-05R column in elderly patients suffering from septic shock. Am J Med Sci. 2007;334(4):244-7.

124. Nakamura T, Matsuda T, Suzuki Y, Shoji H, Koide H. Polymyxin B-immobilized fiber hemoperfusion in patients with sepsis. Dialysis and Transplantation. 2003;32(10):602-5.

125. Nakamura T, Sato E, Fujiwara N, Kawagoe Y, Maeda S, Yamagishi S. Suppression of high-mobility group box-1 and receptor for advanced glycation end-product axis by polymyxin B-immobilized fiber hemoperfusion in septic shock patients. J Crit Care. 2011;26(6):546-9.

126. Nakamura T, Suzuki T, Kawagoe Y, Koide H. Polymyxin B-immobilized fiber hemoperfusion attenuates increased plasma atrial natriuretic peptide and brain natriuretic Peptide levels in patients with septic shock. Asaio j. 2008;54(2):210-3.

127. Nakamura T, Suzuki Y, Shimada N, Ebihara I, Shoji H, Koide H. Hemoperfusion with polymyxin B-immobilized fiber attenuates the increased plasma levels of thrombomodulin and von Willebrand factor from patients with septic shock. Blood Purif. 1998;16(4):179-86.

128. Nakamura T, Ushiyama C, Suzuki S, Shoji H, Shimada N, Ebihara I, Koide H. Polymyxin b-immobilized fiber reduces increased plasma endothelin-1 concentrations in hemodialysis patients with sepsis. Ren Fail. 2000;22(2):225-34.

129. Nakamura Y, Kitamura T, Kiyomi F, Hayakawa M, Hoshino K, Kawano Y, et al. Potential survival benefit of polymyxin B hemoperfusion in patients with septic shock: a propensity-matched cohort study. Crit Care. 2017;21(1):134.

130. Nakata H, Yamakawa K, Kabata D, Umemura Y, Ogura H, Gando S, et al. Identifying Septic Shock Populations Benefitting From Polymyxin B Hemoperfusion: A Prospective Cohort Study Incorporating a Restricted Cubic Spline Regression Model. Shock. 2020;54(5):667-74.

131. Ono S, Tsujimoto H, Matsumoto A, Ikuta S, Kinoshita M, Mochizuki H. Modulation of human leukocyte antigen-DR on monocytes and CD16 on granulocytes in patients with septic shock using hemoperfusion with polymyxin B-immobilized fiber. Am J Surg. 2004;188(2):150-6.

132. Saito N, Sugiyama K, Ohnuma T, Kanemura T, Nasu M, Yoshidomi Y, et al. Efficacy of polymyxin B-immobilized fiber hemoperfusion for patients with septic shock caused by Gram-negative bacillus infection. PLoS One. 2017;12(3):e0173633.

133. Takahashi M, Takeuchi H, Kawakubo H, Nakamura R, Takahashi T, Wada N, et al. Effectiveness of polymyxin B-direct hemoperfusion (PMX-DHP) therapy using a polymyxin B-immobilized fiber column in patients with post-esophagectomy sepsis. Esophagus. 2014;11(3):189-96.

134. Tanaka T, Tabata T, Fujino K, Tsujita Y, Eguchi Y. "Impact of timing of polymyxin B-immobilized fiber column direct hemoperfusion on outcome in patients with septic shock: a single-center observational study". Acute Med Surg. 2020;7(1):e446.

135. Kase Y, Obata T, Okamoto Y, Iwai K, Saito K, Yokoyama K, et al. Removal of 2-arachidonylglycerol by direct hemoperfusion therapy with polymyxin B immobilized fibers benefits patients with septic shock. Ther Apher Dial. 2008;12(5):374-80.

136. Cutuli SL, Carelli S, Cascarano L, Cicconi S, Silvestri D, Cicetti M, et al. Clinical implications of endotoxin activity and Polymyxin-B hemoperfusion in critically ill patients with septic cardiomyopathy: A single-center, retrospective, observational study. Artif Organs. 2023;47(12):1865-73.

137. Cutuli SL, De Rosa S, Ferrer R, Ruiz-Rodriguez JC, Forfori F, Ronco C, Antonelli M. Endotoxin activity trend and multi-organ dysfunction in critically ill patients with septic shock, who received Polymyxin-B hemadsorption: A multicenter, prospective, observational study. Artif Organs. 2023;47(8):1361-70.

138. Osawa I, Goto T, Kudo D, Hayakawa M, Yamakawa K, Kushimoto S, et al. Targeted therapy using polymyxin B hemadsorption in patients with sepsis: a post-hoc analysis of the JSEPTIC-DIC study and the EUPHRATES trial. Crit Care. 2023;27(1):245.

139. Shibata M, Miyamoto K, Kato S. Comparison of the circulatory effects of continuous renal replacement therapy using AN69ST and polysulfone membranes in septic shock patients: A retrospective observational study. Ther Apher Dial. 2020;24(5):561-7.

140. Hayashi K, Sasabuchi Y, Matsui H, Nakajima M, Ohbe H, Fushimi K, et al. Additive effectiveness of acrylonitrile-co-methallyl sulfonate surface-treated membranes in the treatment of pneumonia: A propensity score-matched retrospective cohort study. Artif Organs. 2023;47(2):408-16.

141. Chelazzi C, Villa G, D'Alfonso MG, Mancinelli P, Consales G, Berardi M, et al. Hemodialysis with High Cut-Off Hemodialyzers in Patients with Multi-Drug Resistant Gram-Negative Sepsis and Acute Kidney Injury: A Retrospective, Case-Control Study. Blood Purif. 2016;42(3):186-93.

142. Zhang Y, Zhang Y, Pan J. Efficacy Evaluation of High-Volume Hemofiltration in Patients with Severe Acute Respiratory Distress Syndrome. Evid Based Complement Alternat Med. 2022;2022:9488047.

143. Eichhorn T, Hartmann J, Harm S, Linsberger I, König F, Valicek G, et al. Clearance of Selected Plasma Cytokines with Continuous Veno-Venous Hemodialysis Using Ultraflux EMiC2 versus Ultraflux AV1000S. Blood Purif. 2017;44(4):260-6.

144. Hörner C, Schuster S, Plachky J, Hofer S, Martin E, Weigand MA. Hemofiltration and immune response in severe sepsis. J Surg Res. 2007;142(1):59-65.

145. Lumlertgul N, Srisawat N. The haemodynamic effects of oXiris haemofilter in septic shock patients requiring renal support: A single-centre experience. Int J Artif Organs. 2021;44(1):17-24.

146. Mariano F, Tetta C, Guida G, Triolo G, Camussi G. Hemofiltration reduces the serum priming activity on neutrophil chemiluminescence in septic patients. Kidney Int. 2001;60(4):1598-605.

147. Pestaña D, Casanova E, Villagrán MJ, Tormo C, Pérez-Chrzanowska H, Redondo J, et al. Continuous hemofiltration in hyperthermic septic shock patients. J Trauma. 2007;63(4):751-6.

148. Ueno T, Ikeda T, Okihara M, Akashi I, Yokoyama T, Kihara Y, et al. Cytokine modulation in abdominal septic shock via the crucial role of IL-6 signaling in endothelial dysfunction. Front Med (Lausanne). 2023;10:1042487.

149. Wang J, Wei SR, Ding T, Zhang LP, Weng ZH, Cheng M, et al. Continuous renal replacement therapy with oXiris(®) in patients with hematologically malignant septic shock: A retrospective study. World J Clin Cases. 2023;11(26):6073-82.

150. Yaroustovsky M, Abramyan M, Krotenko N, Popov D, Plyushch M, Rogalskaya E. A pilot study of selective lipopolysaccharide adsorption and coupled plasma filtration and adsorption in adult patients with severe sepsis. Blood Purif. 2015;39(1-3):210-7.

151. Abdul Cader R, Abdul Gafor H, Mohd R, Yen Kong W, Arshad N, Kong N. Coupled Plasma Filtration and Adsorption (CPFA): A Single Center Experience. Nephrourol Mon. 2013;5(4):891-6.

152. Wendel Garcia PD, Hilty MP, Held U, Kleinert EM, Maggiorini M. Cytokine adsorption in severe, refractory septic shock. Intensive Care Med. 2021;47(11):1334-6.

153. Friesecke S, Stecher SS, Gross S, Felix SB, Nierhaus A. Extracorporeal cytokine elimination as rescue therapy in refractory septic shock: a prospective single-center study. J Artif Organs. 2017;20(3):252-9.

154. Schefold JC, von Haehling S, Corsepius M, Pohle C, Kruschke P, Zuckermann H, et al. A novel selective extracorporeal intervention in sepsis: immunoadsorption of endotoxin, interleukin 6, and complement-activating product 5a. Shock. 2007;28(4):418-25.

155. Yaroustovsky M, Abramyan M, Krotenko N, Popov D, Plyushch M, Popok Z. Endotoxin adsorption using polymyxin B immobilized fiber cartridges in severe sepsis patients following cardiac surgery. Int J Artif Organs. 2014;37(4):299-307.

156. Yuan KC, Wang SY, Yu MC, Hsu YP, Pan HC, Chen YC. Efficacy of postoperative polymyxin B hemoperfusion in secondary peritonitis patients with septic shock: a propensity-matched analysis. Int J Artif Organs. 2017;39(12):603-10.

157. Zagli G, Bonizzoli M, Spina R, Cianchi G, Pasquini A, Anichini V, et al. Effects of hemoperfusion with an immobilized polymyxin-B fiber column on cytokine plasma levels in patients with abdominal sepsis. Minerva Anestesiol. 2010;76(6):405-12.

158. Ebihara I, Hirayama K, Kaneko S, Nagai M, Ogawa Y, Fujita S, et al. Vascular endothelial growth factor and soluble fms-like tyrosine kinase-1 in septic shock patients treated with direct hemoperfusion with a polymyxin B-immobilized fiber column. Ther Apher Dial. 2008;12(4):285-91.

159. Ebihara I, Hirayama K, Nagai K, Kakita T, Miyamoto Y, Nagai M, et al. Angiopoietin balance in septic shock patients treated by direct hemoperfusion with polymyxin b-immobilized fiber. Ther Apher Dial. 2009;13(6):520-7.

160. Ebihara I, Hirayama K, Nagai M, Koda M, Gunji M, Okubo Y, et al. Soluble vascular endothelial-cadherin levels in patients with sepsis treated with direct hemoperfusion with a polymyxin B-immobilized fiber column. Ther Apher Dial. 2014;18(3):272-8.

161. Ebihara I, Hirayama K, Nagai M, Shiina E, Koda M, Gunji M, et al. Angiopoietin Balance in Septic Shock Patients With Acute Kidney Injury: Effects of Direct Hemoperfusion With Polymyxin B-Immobilized Fiber. Ther Apher Dial. 2016;20(4):368-75.

162. Ebihara I, Nakamura T, Shimada N, Shoji H, Koide H. Effect of hemoperfusion with polymyxin B-immobilized fiber on plasma endothelin-1 and endothelin-1 mRNA in monocytes from patients with sepsis. Am J Kidney Dis. 1998;32(6):953-61.

163. Miyamoto K, Kawazoe Y, Negi S, Shibata N, Ogawa A, Shima N, et al. Effects of prolonged direct hemoperfusion using a polymyxin B immobilized fiber cartridge on interleukin-6 concentration in patients with septic shock: a prospective exploratory trial. Renal Replacement Therapy. 2019;5(1):20.

164. Kojika M, Sato N, Yaegashi Y, Suzuki Y, Suzuki K, Nakae H, Endo S. Endotoxin adsorption therapy for septic shock using polymyxin B-immobilized fibers (PMX): evaluation by high-sensitivity endotoxin assay and measurement of the cytokine production capacity. Ther Apher Dial. 2006;10(1):12-8.

165. Kodama M, Tani T, Hanasawa K, Hirata K, Hirasawa H, Oda S, et al. Treatment of sepsis by plasma endotoxin removal: hemoperfusion using a polymyxin-B immobilized column. Journal of Endotoxin Research. 1997;4(4):293-300.

166. Kushi H, Miki T, Nakahara J, Okamoto K, Kawahara Y, Saito T, Tanjoh K. Hemoperfusion with an immobilized polymyxin B fiber column improves tissue oxygen metabolism. Ther Apher Dial. 2006;10(5):430-5.

167. Kushi H, Miki T, Nakahara J, Okamoto K, Kawahara Y, Saito T, Tanjoh K. Hemoperfusion with an immobilized polymyxin B fiber column reduces circulating interleukin-8 concentrations. Ther Apher Dial. 2006;10(5):425-9.

168. Kushi H, Miki T, Nakahara J, Okamoto K, Saito T, Tanjoh K. Hemoperfusion with an immobilized polymyxin B column reduces the blood level of neutrophil elastase. Blood Purif. 2006;24(2):212-7.

169. Kushi H, Miki T, Okamaoto K, Nakahara J, Saito T, Tanjoh K. Early hemoperfusion with an immobilized polymyxin B fiber column eliminates humoral mediators and improves pulmonary oxygenation. Crit Care. 2005;9(6):R653-61.

170. Kushi H, Miki T, Sakagami Y, Sato J, Saito T, Tanjoh K. Hemoperfusion with an immobilized polymyxin B fiber column decreases macrophage and monocyte activity. Therapeutic Apheresis and Dialysis. 2009;13(6):515-9.

171. Kushi H, Miki T, Sakagami Y, Sato J, Saito T, Tanjoh K. Hemoperfusion with a polymyxin B fiber column decreases clotting activity. Ther Apher Dial. 2009;13(6):528-33.

172. Kushi H, Nakahara J, Miki T, Okamoto K, Saito T, Tanjo K. Hemoperfusion with an immobilized polymyxin B fiber column inhibits activation of vascular endothelial cells. Ther Apher Dial. 2005;9(4):303-7.

173. Maynar J, Martínez-Sagasti F, Herrera-Gutiérrez M, Martí F, Candel FJ, Belda J, et al. Direct hemoperfusion with polymyxin B-immobilized cartridge in severe sepsis due to intestinal perforation: hemodynamic findings and clinical considerations in anticoagulation therapy. Rev Esp Quimioter. 2013;26(2):151-8.

174. Mitaka C, Tsuchida N, Kawada K, Nakajima Y, Imai T, Sasaki S. A longer duration of polymyxin B-immobilized fiber column hemoperfusion improves pulmonary oxygenation in patients with septic shock. Shock. 2009;32(5):478-83.

175. Navas A, Ferrer R, Martínez ML, Gomà G, Gili G, Masip J, et al. Impact of hemoperfusion with polymyxin B added to hemofiltration in patients with endotoxic shock: a case-control study. Ann Intensive Care. 2018;8(1):121.

176. Ruberto F, Pugliese F, D'Alio A, Martelli S, Bruno K, Marcellino V, et al. Clinical effects of use polymyxin B fixed on fibers in liver transplant patients with severe sepsis or septic shock. Transplant Proc. 2007;39(6):1953-5.

177. Ruberto F, Pugliese F, D'Alio A, Martelli S, Bruno K, Marcellino V, et al. Clinical effects of direct hemoperfusion using a polymyxin-B immobilized column in solid organ transplanted patients with signs of severe sepsis and septic shock. A pilot study. Int J Artif Organs. 2007;30(10):915-22.

178. Sakamoto Y, Mashiko K, Matsumoto H, Hara Y, Kutsukata N, Yamamoto Y. Relationship between effect of polymyxin B-immobilized fiber and high-mobility group box-1 protein in septic shock patients. Asaio j. 2007;53(3):324-8.

179. Sakamoto Y, Mashiko K, Obata T, Matsumoto H, Hara Y, Kutsukata N, Yamamoto Y. Clinical responses and improvement of some laboratory parameters following polymyxin B-immobilized fiber treatment in septic shock. Asaio j. 2007;53(5):646-50.

180. Sakamoto Y, Mashiko K, Obata T, Matsumoto H, Hara Y, Kutsukata N, Yamamoto Y. Relationship between treatment resistance to hemoperfusion using a polymyxin B-immobilized fiber column and oxidative stress. Asaio j. 2008;54(4):412-5.

181. Shimizu T, Hanasawa K, Sato K, Umeki M, Koga N, Naganuma T, et al. Direct hemoperfusion with polymyxin-B-immobilized fiber columns improves septic hypotension and reduces inflammatory mediators in septic patients with colorectal perforation. Langenbecks Arch Surg. 2009;394(2):303-11.

182. Shimizu T, Hanasawa K, Tani T, Endo Y, Kurumi Y, Ikeda T, et al. Changes in circulating levels of calcitonin gene-related peptide and nitric oxide metabolites in septic patients during direct hemoperfusion with polymyxin B-immobilized fiber. Blood Purif. 2003;21(3):237-43.

183. Takeyama N, Noguchi H, Hirakawa A, Kano H, Morino K, Obata T, et al. Time to initiation of treatment with polymyxin B cartridge hemoperfusion in septic shock patients. Blood Purif. 2012;33(4):252-6.

184. Tani T, Hanasawa K, Endo Y, Yoshioka T, Kodama M, Kaneko M, et al. Therapeutic apheresis for septic patients with organ dysfunction: hemoperfusion using a polymyxin B immobilized column. Artif Organs. 1998;22(12):1038-44.

185. Matsuno N, Ikeda T, Ikeda K, Nagao T. Beneficial effect of blood purification combined endotoxin absorption column on septic multiple organ failure patients. Annals of Fundeni Hospital. 1997;2:187-91.

186. Dahaba AA, Elawady GA, Rehak PH, List WF. Procalcitonin and proinflammatory cytokine clearance during continuous venovenous haemofiltration in septic patients. Anaesth Intensive Care. 2002;30(3):269-74.

187. Heering P, Morgera S, Schmitz FJ, Schmitz G, Willers R, Schultheiss HP, et al. Cytokine removal and cardiovascular hemodynamics in septic patients with continuous venovenous hemofiltration. Intensive Care Med. 1997;23(3):288-96.

188. Hoffmann JN, Hartl WH, Deppisch R, Faist E, Jochum M, Inthorn D. Effect of hemofiltration on hemodynamics and systemic concentrations of anaphylatoxins and cytokines in human sepsis. Intensive Care Med. 1996;22(12):1360-7.

189. Klouche K, Cavadore P, Portales P, Clot J, Canaud B, Béraud JJ. Continuous veno-venous hemofiltration improves hemodynamics in septic shock with acute renal failure without modifying TNFalpha and IL6 plasma concentrations. J Nephrol. 2002;15(2):150-7.

190. Matsuda K, Moriguchi T, Harii N, Yanagisawa M, Harada D, Sugawara H. Comparison of efficacy between continuous hemodiafiltration with a PMMA high-performance membrane dialyzer and a PAN membrane hemofilter in the treatment of septic shock patients with acute renal failure. Contrib Nephrol. 2011;173:182-90.

191. Morgera S, Rocktäschel J, Haase M, Lehmann C, von Heymann C, Ziemer S, et al. Intermittent high permeability hemofiltration in septic patients with acute renal failure. Intensive Care Med. 2003;29(11):1989-95.

192. Bellomo R, Tipping P, Boyce N. Continuous veno-venous hemofiltration with dialysis removes cytokines from the circulation of septic patients. Crit Care Med. 1993;21(4):522-6.

193. Bellomo R, Tipping P, Boyce N. Interleukin-6 and interleukin-8 extraction during continuous venovenous hemodiafiltration in septic acute renal failure. Ren Fail. 1995;17(4):457-66.

194. Servillo G, Vargas M, Pastore A, Procino A, Iannuzzi M, Capuano A, et al. Immunomodulatory effect of continuous venovenous hemofiltration during sepsis: preliminary data. Biomed Res Int. 2013;2013:108951.

195. Nakada TA, Oda S, Matsuda K, Sadahiro T, Nakamura M, Abe R, Hirasawa H. Continuous hemodiafiltration with PMMA Hemofilter in the treatment of patients with septic shock. Mol Med. 2008;14(5-6):257-63.

196. Shiga H, Hirasawa H, Nishida O, Oda S, Nakamura M, Mashiko K, et al. Continuous hemodiafiltration with a cytokine-adsorbing hemofilter in patients with septic shock: a preliminary report. Blood Purif. 2014;38(3-4):211-8.

197. Mao HJ, Yu S, Yu XB, Zhang B, Zhang L, Xu XR, et al. Effects of coupled plasma filtration adsorption on immune function of patients with multiple organ dysfunction syndrome. Int J Artif Organs. 2009;32(1):31-8.

198. Ronco C, Brendolan A, Lonnemann G, Bellomo R, Piccinni P, Digito A, et al. A pilot study of coupled plasma filtration with adsorption in septic shock. Crit Care Med. 2002;30(6):1250-5.

199. Knaup H, Stahl K, Schmidt BMW, Idowu TO, Busch M, Wiesner O, et al. Early therapeutic plasma exchange in septic shock: a prospective open-label nonrandomized pilot study focusing on safety, hemodynamics, vascular barrier function, and biologic markers. Crit Care. 2018;22(1):285.

200. Bengsch S, Boos KS, Nagel D, Seidel D, Inthorn D. Extracorporeal plasma treatment for the removal of endotoxin in patients with sepsis: clinical results of a pilot study. Shock. 2005;23(6):494-500.

201. Westendorp RG, Brand A, Haanen J, van Hinsbergh VW, Thompson J, van Furth R, Meinders EA. Leukaplasmapheresis in meningococcal septic shock. Am J Med. 1992;92(5):577-8.

202. Yaroustovsky M, Abramyan M, Krotenko N, Popov D, Plyushch M, Rogalskaya E, Nazarova H. Combined extracorporeal therapy for severe sepsis in patients after cardiac surgery. Blood Purif. 2014;37(1):39-46.

203. Zheng S, Weng Q, Wu W, Ding G. Blood purification treatment initiated at the time of sepsis diagnosis effectively attenuates serum HMGB1 upregulation and improves patient prognosis. Exp Ther Med. 2017;14(4):3029-35.

204. Li S, Wang A, Pan X. Effect of continuous blood purification on sepsis caused by abdominal infection or pulmonary infection and its effect on cytokines and residual renal function. Acta Medica Mediterranea. 2020;36(4):2563-70.

205. Zu H, Li Q, Huang P, Wang X. Therapeutic Value of Blood Purification and Prognostic Utilities of Early Serum Procalcitonin, C Reactive Protein, and Brain Natriuretic Peptide Levels in Severely Burned Patients with Sepsis. Cell Biochem Biophys. 2015;72(1):259-63.

206. Zeng Y, Wu D, Zhuo X, Song J. Effects of Continuous Blood Purification without Heparin on Strem-1, NSE, and IL-10 Levels In Patients With Sepsis. Cell Mol Biol (Noisy-le-grand). 2022;68(4):178-87.

207. Hawchar F, László I, Öveges N, Trásy D, Ondrik Z, Molnar Z. Extracorporeal cytokine adsorption in septic shock: A proof of concept randomized, controlled pilot study. J Crit Care. 2019;49:172-8.

208. Schädler D, Pausch C, Heise D, Meier-Hellmann A, Brederlau J, Weiler N, et al. The effect of a novel extracorporeal cytokine hemoadsorption device on IL-6 elimination in septic patients: A randomized controlled trial. PLoS One. 2017;12(10):e0187015.

209. Chen SH, Chan WS, Liu CM, Chiu CT, Chao A, Wu VC, et al. Effects of endotoxin adsorber hemoperfusion on sublingual microcirculation in patients with septic shock: a randomized controlled trial. Ann Intensive Care. 2020;10(1):80.

210. Reinhart K, Meier-Hellmann A, Beale R, Forst H, Boehm D, Willatts S, et al. Open randomized phase II trial of an extracorporeal endotoxin adsorber in suspected Gram-negative sepsis. Crit Care Med. 2004;32(8):1662-8.

211. Shum HP, Leung YW, Lam SM, Chan KC, Yan WW. Alteco endotoxin hemoadsorption in Gram-negative septic shock patients. Indian J Crit Care Med. 2014;18(12):783-8.

212. Huang Z, Wang SR, Su W, Liu JY. Removal of humoral mediators and the effect on the survival of septic patients by hemoperfusion with neutral microporous resin column. Ther Apher Dial. 2010;14(6):596-602.

213. Huang Z, Wang SR, Yang ZL, Liu JY. Effect on extrapulmonary sepsis-induced acute lung injury by hemoperfusion with neutral microporous resin column. Ther Apher Dial. 2013;17(4):454-61.

214. Lipcsey M, Tenhunen J, Pischke SE, Kuitunen A, Flaatten H, De Geer L, et al. Endotoxin Removal in Septic Shock with the Alteco LPS Adsorber Was Safe But Showed no Benefit Compared to Placebo in the Double-Blind Randomized Controlled Trial-the Asset Study. Shock. 2020;54(2):224-31.

215. Vincent JL, Laterre PF, Cohen J, Burchardi H, Bruining H, Lerma FA, et al. A pilot-controlled study of a polymyxin B-immobilized hemoperfusion cartridge in patients with severe sepsis secondary to intra-abdominal infection. Shock. 2005;23(5):400-5.

216. Cantaluppi V, Assenzio B, Pasero D, Romanazzi GM, Pacitti A, Lanfranco G, et al. Polymyxin-B hemoperfusion inactivates circulating proapoptotic factors. Intensive Care Med. 2008;34(9):1638-45.

217. Coudroy R, Payen D, Launey Y, Lukaszewicz AC, Kaaki M, Veber B, et al. Modulation by Polymyxin-B Hemoperfusion of Inflammatory Response Related to Severe Peritonitis. Shock. 2017;47(1):93-9.

218. Cruz DN, Antonelli M, Fumagalli R, Foltran F, Brienza N, Donati A, et al. Early use of polymyxin B hemoperfusion in abdominal septic shock: the EUPHAS randomized controlled trial. Jama. 2009;301(23):2445-52.

219. Dellinger RP, Bagshaw SM, Antonelli M, Foster DM, Klein DJ, Marshall JC, et al. Effect of Targeted Polymyxin B Hemoperfusion on 28-Day Mortality in Patients With Septic Shock and Elevated Endotoxin Level: The EUPHRATES Randomized Clinical Trial. Jama. 2018;320(14):1455-63.

220. Antonelli M, Fumagalli R, Cruz DN, Brienza N, Giunta F. PMX endotoxin removal in the clinical practice: results from the EUPHAS trial. Contrib Nephrol. 2010;167:83-90.

221. Nakamura T, Ebihara I, Shoji H, Ushiyama C, Suzuki S, Koide H. Treatment with polymyxin B-immobilized fiber reduces platelet activation in septic shock patients: decrease in plasma levels of soluble P-selectin, platelet factor 4 and beta-thromboglobulin. Inflamm Res. 1999;48(4):171-5.

222. Nakamura T, Kawagoe Y, Matsuda T, Ueda Y, Koide H. Effects of polymyxin B immobilized fiber on urinary N-acetyl-beta-glucosaminidase in patients with severe sepsis. Asaio j. 2004;50(6):563-7.

223. Nakamura T, Kawagoe Y, Suzuki T, Shoji H, Ueda Y, Kobayashi N, Koide H. Changes in plasma interleukin-18 by direct hemoperfusion with polymyxin B-immobilized fiber in patients with septic shock. Blood Purif. 2005;23(6):417-20.

224. Nakamura T, Ushiyama C, Suzuki Y, Shoji H, Osada S, Shimada N, Koide H. Effect of polymyxin B-immobilized fibre on various mediators in patients with hypothermic sepsis. Clinical Intensive Care. 2001;12(5-6):223-8.

225. Nakamura T, Ushiyama C, Suzuki Y, Shoji H, Shimada N, Koide H. Hemoperfusion with polymyxin B immobilized fibers for urinary albumin excretion in septic patients with trauma. Asaio j. 2002;48(3):244-8.

226. Nemoto H, Nakamoto H, Okada H, Sugahara S, Moriwaki K, Arai M, et al. Newly developed immobilized polymyxin B fibers improve the survival of patients with sepsis. Blood Purif. 2001;19(4):361-8; discussion 8-9.

227. Payen DM, Guilhot J, Launey Y, Lukaszewicz AC, Kaaki M, Veber B, et al. Early use of polymyxin B hemoperfusion in patients with septic shock due to peritonitis: a multicenter randomized control trial. Intensive Care Med. 2015;41(6):975-84.

228. Srisawat N, Tungsanga S, Lumlertgul N, Komaenthammasophon C, Peerapornratana S, Thamrongsat N, et al. The effect of polymyxin B hemoperfusion on modulation of human leukocyte antigen DR in severe sepsis patients. Crit Care. 2018;22(1):279.

229. Rey S, Kulabukhov VM, Popov A, Nikitina O, Berdnikov G, Magomedov M, et al. HEMOPERFUSION USING THE LPS-SELECTIVE MESOPOROUS POLYMERIC ADSORBENT IN SEPTIC SHOCK: A MULTICENTER RANDOMIZED CLINICAL TRIAL. Shock. 2023;59(6):846-54.

230. Broman ME, Hansson F, Vincent JL, Bodelsson M. Endotoxin and cytokine reducing properties of the oXiris membrane in patients with septic shock: A randomized crossover double-blind study. PLoS One. 2019;14(8):e0220444.

231. Peng Y, Yuan Z, Li H. Removal of inflammatory cytokines and endotoxin by veno-venous continuous renal replacement therapy for burned patients with sepsis. Burns. 2005;31(5):623-8.

232. Feng J, Zhang S, Ai T, Wang L, Gao Y, Li W, Zhu M. Effect of CRRT with oXiris filter on hemodynamic instability in surgical septic shock with AKI: A pilot randomized controlled trial. Int J Artif Organs. 2022;45(10):801-8.

233. Wu S, Xu T, Wu C, Lei X, Tian X. Continuous renal replacement therapy in sepsis-associated acute kidney injury: Effects on inflammatory mediators and coagulation function. Asian J Surg. 2021;44(10):1254-9.

234. An N, Chen R, Bai Y, Xu M. Efficacy and prognosis of continuous renal replacement therapy at different times in the treatment of patients with sepsis-induced acute kidney injury. Am J Transl Res. 2021;13(6):7124-31.

235. Xu Z, Zhang, Y., Li, P., Duan, F., Li, C., Yu, J. . Clinical applicatin of continous renal replacemnet therapy in patients with acute renal injury caused by septic shock. Acta medica mediterranea. 2022:38:1447.

236. Braun N, Rosenfeld S, Giolai M, Banzhaf W, Fretschner R, Warth H, et al. Effect of continuous hemodiafiltration on IL-6, TNF-alpha, C3a, and TCC in patients with SIRS/septic shock using two different membranes. Contrib Nephrol. 1995;116:89-98.

237. Chancharoenthana W, Tiranathanagul K, Srisawat N, Susantitaphong P, Leelahavanichkul A, Praditpornsilpa K, et al. Enhanced vascular endothelial growth factor and inflammatory cytokine removal with online hemodiafiltration over high-flux hemodialysis in sepsis-related acute kidney injury patients. Ther Apher Dial. 2013;17(5):557-63.

238. Haase M, Bellomo R, Baldwin I, Haase-Fielitz A, Fealy N, Davenport P, et al. Hemodialysis membrane with a high-molecular-weight cutoff and cytokine levels in sepsis complicated by acute renal failure: a phase 1 randomized trial. Am J Kidney Dis. 2007;50(2):296-304.

239. Ferrari F, Husain-Syed F, Milla P, Lorenzin A, Scudeller L, Sartori M, et al. Clinical Assessment of Continuous Hemodialysis with the Medium Cutoff EMiC®2 Membrane in Patients with Septic Shock. Blood Purif. 2022;51(11):912-22.

240. Bi X, Ying Xian, Mingliang Li, Yuan, X., Xie, D., Xu, W., You, J., Zhang, K. Effects of HVHF therapy on serum inflammatory factors, pulmonary function, and hemodynamic indexes of patients with sepsis complicated by acute respiratory distress syndrome. Int J Clin Exp Med. 2019;12:6014-9.

241. Cole L, Bellomo R, Hart G, Journois D, Davenport P, Tipping P, Ronco C. A phase II randomized, controlled trial of continuous hemofiltration in sepsis. Crit Care Med. 2002;30(1):100-6.

242. Cole L, Bellomo R, Journois D, Davenport P, Baldwin I, Tipping P. High-volume haemofiltration in human septic shock. Intensive Care Med. 2001;27(6):978-86.

243. Ghani RA, Zainudin S, Ctkong N, Rahman AF, Wafa SR, Mohamad M, et al. Serum IL-6 and IL-1-ra with sequential organ failure assessment scores in septic patients receiving high-volume haemofiltration and continuous venovenous haemofiltration. Nephrology (Carlton). 2006;11(5):386-93.

244. Haase M, Silvester W, Uchino S, Goldsmith D, Davenport P, Tipping P, et al. A pilot study of high-adsorption hemofiltration in human septic shock. Int J Artif Organs. 2007;30(2):108-17.

245. Morgera S, Haase M, Kuss T, Vargas-Hein O, Zuckermann-Becker H, Melzer C, et al. Pilot study on the effects of high cutoff hemofiltration on the need for norepinephrine in septic patients with acute renal failure. Crit Care Med. 2006;34(8):2099-104.

246. Morgera S, Haase M, Rocktäschel J, Böhler T, von Heymann C, Vargas-Hein O, et al. High permeability haemofiltration improves peripheral blood mononuclear cell proliferation in septic patients with acute renal failure. Nephrol Dial Transplant. 2003;18(12):2570-6.

247. Guo J, Tao W, Tang D, Zhang J. Th17/regulatory T cell imbalance in sepsis patients with multiple organ dysfunction syndrome: attenuated by high-volume hemofiltration. Int J Artif Organs. 2017;40(11):607-14.

248. Joannes-Boyau O, Honoré PM, Perez P, Bagshaw SM, Grand H, Canivet JL, et al. High-volume versus standard-volume haemofiltration for septic shock patients with acute kidney injury (IVOIRE study): a multicentre randomized controlled trial. Intensive Care Med. 2013;39(9):1535-46.

249. Park JT, Lee H, Kee YK, Park S, Oh HJ, Han SH, et al. High-Dose Versus Conventional-Dose Continuous Venovenous Hemodiafiltration and Patient and Kidney Survival and Cytokine Removal in Sepsis-Associated Acute Kidney Injury: A Randomized Controlled Trial. Am J Kidney Dis. 2016;68(4):599-608.

250. Peng Z, Pai P, Han-Min W, Jun Z, Hong-Bao L, Rong L, Chen H. Evaluation of the effects of pulse high-volume hemofiltration in patients with severe sepsis: a preliminary study. Int J Artif Organs. 2010;33(8):505-11.

251. Peng Z, Pai P, Hong-Bao L, Rong L, Han-Min W, Chen H. The impacts of continuous veno-venous hemofiltration on plasma cytokines and monocyte human leukocyte antigen-DR expression in septic patients. Cytokine. 2010;50(2):186-91.

252. Gao Y, Huang X, Yang Y, Lei Z, Chen Q, Guo X, et al. Clinical analysis of AN69ST membrane continuous venous hemofiltration in the treatment of severe sepsis. Open Med (Wars). 2023;18(1):20230784.

253. An MM, Liu CX, Gong P. Effects of continuous renal replacement therapy on inflammation-related anemia, iron metabolism and prognosis in sepsis patients with acute kidney injury. World J Emerg Med. 2023;14(3):186-92.

254. Nakamura Y, Hatomoto H, Yamasaki S, Yamauchi K, Kiyomi F, Hoshino K, et al. Comparison of the cytokine adsorption ability in continuous renal replacement therapy using polyethyleneimine-coated polyacrylonitrile (AN69ST) or polymethylmethacrylate (PMMA) hemofilters: a pilot single-center open-label randomized control trial. Eur J Med Res. 2023;28(1):208.

255. Stahl K, Wand P, Seeliger B, Wendel-Garcia PD, Schmidt JJ, Schmidt BMW, et al. Clinical and biochemical endpoints and predictors of response to plasma exchange in septic shock: results from a randomized controlled trial. Crit Care. 2022;26(1):134.

256. Hamishehkar H, Beigmohammadi MT, Abdollahi M, Mousavi S, Ziaie S, Sharifian RA, et al. Pro-inflammatory cytokine profile of critically ill septic patients following therapeutic plasma exchange. Transfus Apher Sci. 2013;48(1):75-8.

257. Giménez-Esparza C, Portillo-Requena C, Colomina-Climent F, Allegue-Gallego JM, Galindo-Martínez M, Mollà-Jiménez C, et al. The premature closure of ROMPA clinical trial: mortality reduction in septic shock by plasma adsorption. BMJ Open. 2019;9(12):e030139.

258. Hassan J, Cader RA, Kong NC, Mohd M, Rahman AR, Hod R. Coupled Plasma Filtration Adsorption (CPFA) plus Continuous Veno-Venous Haemofiltration (CVVH) versus CVVH alone as an adjunctive therapy in the treatment of sepsis. Excli j. 2013;12:681-92.

259. Hu D, Sun S, Zhu B, Mei Z, Wang L, Zhu S, Zhao W. Effects of coupled plasma filtration adsorption on septic patients with multiple organ dysfunction syndrome. Ren Fail. 2012;34(7):834-9.

260. Livigni S, Bertolini G, Rossi C, Ferrari F, Giardino M, Pozzato M, Remuzzi G. Efficacy of coupled plasma filtration adsorption (CPFA) in patients with septic shock: a multicenter randomised controlled clinical trial. BMJ Open. 2014;4(1):e003536.

261. Chu L, Li G, Yu Y, Bao X, Wei H, Hu M. Clinical effects of hemoperfusion combined with pulse high-volume hemofiltration on septic shock. Medicine (Baltimore). 2020;99(9):e19058.

262. Caliezi C, Zeerleder S, Redondo M, Regli B, Rothen HU, Zürcher-Zenklusen R, et al. C1-inhibitor in patients with severe sepsis and septic shock: beneficial effect on renal dysfunction. Crit Care Med. 2002;30(8):1722-8.

263. Igonin AA, Protsenko DN, Galstyan GM, Vlasenko AV, Khachatryan NN, Nekhaev IV, et al. C1-esterase inhibitor infusion increases survival rates for patients with sepsis*. Crit Care Med. 2012;40(3):770-7.

264. Zeerleder S, Caliezi C, van Mierlo G, Eerenberg-Belmer A, Sulzer I, Hack CE, Wuillemin WA. Administration of C1 inhibitor reduces neutrophil activation in patients with sepsis. Clin Diagn Lab Immunol. 2003;10(4):529-35.

265. Bauer M, Weyland A, Marx G, Bloos F, Weber S, Weiler N, et al. Efficacy and Safety of Vilobelimab (IFX-1), a Novel Monoclonal Anti-C5a Antibody, in Patients With Early Severe Sepsis or Septic Shock-A Randomized, Placebo-Controlled, Double-Blind, Multicenter, Phase IIa Trial (SCIENS Study). Crit Care Explor. 2021;3(11):e0577.

266. Rintala E, Kauppila M, Seppälä OP, Voipio-Pulkki LM, Pettilä V, Rasi V, Kotilainen P. Protein C substitution in sepsis-associated purpura fulminans. Crit Care Med. 2000;28(7):2373-8.

267. Dhainaut JF, Laterre PF, LaRosa SP, Levy H, Garber GE, Heiselman D, et al. The clinical evaluation committee in a large multicenter phase 3 trial of drotrecogin alfa (activated) in patients with severe sepsis (PROWESS): role, methodology, and results. Crit Care Med. 2003;31(9):2291-301.

268. Ely EW, Angus DC, Williams MD, Bates B, Qualy R, Bernard GR. Drotrecogin alfa (activated) treatment of older patients with severe sepsis. Clin Infect Dis. 2003;37(2):187-95.

269. Ely EW, Laterre PF, Angus DC, Helterbrand JD, Levy H, Dhainaut JF, et al. Drotrecogin alfa (activated) administration across clinically important subgroups of patients with severe sepsis. Crit Care Med. 2003;31(1):12-9.

270. Fowler RA, Hill-Popper M, Stasinos J, Petrou C, Sanders GD, Garber AM. Cost-effectiveness of recombinant human activated protein C and the influence of severity of illness in the treatment of patients with severe sepsis. J Crit Care. 2003;18(3):181-91; discussion 91-4.

271. Opal SM, Garber GE, LaRosa SP, Maki DG, Freebairn RC, Kinasewitz GT, et al. Systemic host responses in severe sepsis analyzed by causative microorganism and treatment effects of drotrecogin alfa (activated). Clin Infect Dis. 2003;37(1):50-8.

272. Vincent JL, Nadel S, Kutsogiannis DJ, Gibney RT, Yan SB, Wyss VL, et al. Drotrecogin alfa (activated) in patients with severe sepsis presenting with purpura fulminans, meningitis, or meningococcal disease: a retrospective analysis of patients enrolled in recent clinical studies. Crit Care. 2005;9(4):R331-43.

273. Fry DE, Beilman G, Johnson S, Williams MD, Rodman G, Booth FV, et al. Safety of drotrecogin alfa (activated) in surgical patients with severe sepsis. Surg Infect (Larchmt). 2004;5(3):253-9.

274. Laterre PF, Levy H, Clermont G, Ball DE, Garg R, Nelson DR, et al. Hospital mortality and resource use in subgroups of the Recombinant Human Activated Protein C Worldwide Evaluation in Severe Sepsis (PROWESS) trial. Crit Care Med. 2004;32(11):2207-18.

275. Barie PS, Williams MD, McCollam JS, Bates BM, Qualy RL, Lowry SF, Fry DE. Benefit/risk profile of drotrecogin alfa (activated) in surgical patients with severe sepsis. Am J Surg. 2004;188(3):212-20.

276. Bernard GR, Margolis BD, Shanies HM, Ely EW, Wheeler AP, Levy H, et al. Extended evaluation of recombinant human activated protein C United States Trial (ENHANCE US): a single-arm, phase 3B, multicenter study of drotrecogin alfa (activated) in severe sepsis. Chest. 2004;125(6):2206-16.

277. Davies A, Ridley S, Hutton J, Chinn C, Barber B, Angus DC. Cost effectiveness of drotrecogin alfa (activated) for the treatment of severe sepsis in the United Kingdom. Anaesthesia. 2005;60(2):155-62.

278. de Pont AC, Bakhtiari K, Hutten BA, de Jonge E, Vroom MB, Meijers JC, et al. Recombinant human activated protein C resets thrombin generation in patients with severe sepsis - a case control study. Crit Care. 2005;9(5):R490-7.

279. Gullo A, Iscra F, Di Capua G, Berlot G, Lucangelo U, Peratoner A, et al. Systemic and organ dysfunction response during infusion of recombinant human activated protein C (rhAPC) in severe sepsis and septic shock. Minerva Anestesiol. 2005;71(12):785-801.

280. Higgins TL, Steingrub JS, Tereso GJ, Tidswell MA, McGee WT. Drotrecogin alfa (activated) in sepsis: initial experience with patient selection, cost, and clinical outcomes. J Intensive Care Med. 2005;20(6):339-45.

281. Hjelmgren J, Persson U, Tennvall GR. Local treatment pattern versus trial-based data: a cost-effectiveness analysis of drotrecogin alfa (activated) in the treatment of severe sepsis in Sweden. Am J Ther. 2005;12(5):425-30.

282. Vincent JL, O'Brien J, Jr., Wheeler A, Wittebole X, Garg R, Trzaskoma BL, Sundin DP. Use of an integrated clinical trial database to evaluate the effect of timing of drotrecogin alfa (activated) treatment in severe sepsis. Crit Care. 2006;10(3):R74.

283. Riou França L, Launois R, Le Lay K, Aegerter P, Bouhassira M, Meshaka P, Guidet B. Cost-effectiveness of drotrecogin alfa (activated) in the treatment of severe sepsis with multiple organ failure. Int J Technol Assess Health Care. 2006;22(1):101-8.

284. Kübler A, Mayzner-Zawadzka E, Durek G, Gaszyński W, Karpel E, Mikaszewska-Sokolewicz M, Majak P. Results of severe sepsis treatment program using recombinant human activated protein C in Poland. Med Sci Monit. 2006;12(3):Cr107-12.

285. Bertolini G, Rossi C, Anghileri A, Livigni S, Addis A, Poole D. Use of Drotrecogin alfa (activated) in Italian intensive care units: the results of a nationwide survey. Intensive Care Med. 2007;33(3):426-34.

286. Bilbault P, Lavaux T, Launoy A, Gaub MP, Meyer N, Oudet P, et al. Influence of drotrecogin alpha (activated) infusion on the variation of Bax/Bcl-2 and Bax/Bcl-xl ratios in circulating mononuclear cells: a cohort study in septic shock patients. Crit Care Med. 2007;35(1):69-75.

287. Ernst FR, Johnston JA, Pulgar S, He J, Ball DE, Young JK, Cooper LM. Timing of drotrecogin alfa (activated) initiation in treatment of severe sepsis: a database cohort study of hospital mortality, length of stay, and costs. Curr Med Res Opin. 2007;23(1):235-44.

288. John J, Awab A, Norman D, Dernaika T, Kinasewitz GT. Activated protein C improves survival in severe sepsis patients with elevated troponin. Intensive Care Med. 2007;33(12):2122-8.

289. Kanji S, Perreault MM, Chant C, Williamson D, Burry L. Evaluating the use of Drotrecogin alfa (activated) in adult severe sepsis: a Canadian multicenter observational study. Intensive Care Med. 2007;33(3):517-23.

290. Laterre PF, Nelson DR, Macias W, Abraham E, Sashegyi A, Williams MD, et al. International integrated database for the evaluation of severe sepsis and drotrecogin alfa (activated) therapy: 28-day survival and safety. J Crit Care. 2007;22(2):142-52.

291. Longo CJ, Heyland DK, Fisher HN, Fowler RA, Martin CM, Day AG. A long-term follow-up study investigating health-related quality of life and resource use in survivors of severe sepsis: comparison of recombinant human activated protein C with standard care. Crit Care. 2007;11(6):R128.

292. Payen D, Sablotzki A, Barie PS, Ramsay G, Lowry S, Williams M, et al. International integrated database for the evaluation of severe sepsis and drotrecogin alfa (activated) therapy: analysis of efficacy and safety data in a large surgical cohort. Surgery. 2006;140(5):726-39.

293. Ridley S, Lwin A, Wyncoll D, Lippett S, Watson D, Gunning K, Higgins D. Drotrecogin alfa (activated): diffusion from clinical trials to clinical practice. Eur J Anaesthesiol. 2008;25(3):211-6.

294. Rowan KM, Welch CA, North E, Harrison DA. Drotrecogin alfa (activated): real-life use and outcomes for the UK. Crit Care. 2008;12(2):R58.

295. Wheeler A, Steingrub J, Schmidt GA, Sanchez P, Jacobi J, Linde-Zwirble W, et al. A retrospective observational study of drotrecogin alfa (activated) in adults with severe sepsis: comparison with a controlled clinical trial. Crit Care Med. 2008;36(1):14-23.

296. Camporota L, Corno E, Menaldo E, Smith J, Lei K, Beale R, Wyncoll D. Filter survival time and requirement of blood products in patients with severe sepsis receiving drotrecogin alfa (activated) and requiring renal replacement therapy. Crit Care. 2008;12(6):R163.

297. Janssen van Doorn K, Spapen H, Geers C, Diltoer M, Shabana W. Sepsis-related acute kidney injury: a protective effect of drotrecogin alpha (activated) treatment? Acta Anaesthesiol Scand. 2008;52(9):1259-64.

298. Kanji Z. Utilization and Outcomes of Drotrecogin Alfa (Activated) for Sepsis in Critically Ill Patients at a Community Acute Care Hospital. Canadian Journal of Hospital Pharmacy. 2008;61(6).

299. Behnes M, Brueckmann M, Wiessner M, Kettenmann E, Liebetrau C, Lang S, et al. Time-course of neopterin levels in patients suffering from severe sepsis treated with and without Drotrecogin-alpha (activated). Scand J Infect Dis. 2008;40(6-7):503-8.

300. Sanchez B, Piacentini E, Pradella V, Mignini M, Nava J. Hemodynamic effects of recombinant human activated protein C in patients with septic shock. J Crit Care. 2010;25(2):343-7.

301. Vieillard-Baron A, Caille V, Charron C, Belliard G, Aegerter P, Page B, Jardin F. Reversal of refractory septic shock with drotrecogin alpha (activated). Intensive Care Med. 2009;35(7):1204-9.

302. Crivellari M, Della Valle P, Landoni G, Pappalardo F, Gerli C, Bignami E, et al. Human protein C zymogen concentrate in patients with severe sepsis and multiple organ failure after adult cardiac surgery. Intensive Care Med. 2009;35(11):1959-63.

303. Ahishakiye D, Lorent S, De Backer D, Gottignies P, Vincent JL. Drotrecogin alfa (activated) for severe sepsis: could we consider a shorter treatment period in patients with a favorable course? J Crit Care. 2009;24(4):590-4.

304. Gentry CA, Gross KB, Sud B, Drevets DA. Adverse outcomes associated with the use of drotrecogin alfa (activated) in patients with severe sepsis and baseline bleeding precautions. Crit Care Med. 2009;37(1):19-25.

305. Hodder RV, Hall R, Russell JA, Fisher HN, Lee B. Early drotrecogin alpha (activated) administration in severe sepsis is associated with lower mortality: a retrospective analysis of the Canadian ENHANCE cohort. Crit Care. 2009;13(3):R78.

306. Martin G, Brunkhorst FM, Janes JM, Reinhart K, Sundin DP, Garnett K, Beale R. The international PROGRESS registry of patients with severe sepsis: drotrecogin alfa (activated) use and patient outcomes. Crit Care. 2009;13(3):R103.

307. Polli F, Savioli M, Cugno M, Taccone P, Bellani G, Spanu P, et al. Effects of recombinant human activated protein C on the fibrinolytic system of patients undergoing conventional or tight glycemic control. Minerva Anestesiol. 2009;75(7-8):417-26.

308. Lindenauer PK, Rothberg MB, Nathanson BH, Pekow PS, Steingrub JS. Activated protein C and hospital mortality in septic shock: a propensity-matched analysis. Crit Care Med. 2010;38(4):1101-7.

309. Taylor BJ, Lee SJ, Waxman K. Bleeding complications with Drotrecogin alfa activated (Xigris): a retrospective review of 31 operative and 68 non-operative patients with severe sepsis. Am Surg. 2008;74(10):898-901.

310. Spapen H, Nguyen DN, Troubleyn J, Huyghens L, Schiettecatte J. Drotrecogin alfa (activated) may attenuate severe sepsis-associated encephalopathy in clinical septic shock. Crit Care. 2010;14(2):R54.

311. Steingrub JS, Cheatham ML, Woodward B, Wang HT, Effron MB. A prospective, observational study of Xigris Use in the United States (XEUS). J Crit Care. 2010;25(4):660.e9-16.

312. Abuhasna SH, Z.H. & Jundi, A.H. Assessment of utilization and outcomes of drotrecogin alpha (activated) for critically ill patients with sepsis at a government acute care hospital: A retrospective study. Anaesthesia, Pain and Intensive Care. 2011;15:102-5.

313. Barie PS, Hydo LJ, Shou J, Eachempati SR. Efficacy of therapy with recombinant human activated protein C of critically ill surgical patients with infection complicated by septic shock and multiple organ dysfunction syndrome. Surg Infect (Larchmt). 2011;12(6):443-9.

314. Sadaka F, O'Brien J, Migneron M, Stortz J, Vanston A, Taylor RW. Activated protein C in septic shock: a propensity-matched analysis. Crit Care. 2011;15(2):R89.

315. Boyle A, McKenzie C, Yassin S, McLuckie A, Wyncoll D. Adverse events and clinical outcome associated with drotrecogin alfa-activated: a single-center experience of 498 patients over 8 years. J Crit Care. 2012;27(3):320.e7-12.

316. Casserly B, Gerlach H, Phillips GS, Marshall JC, Lemeshow S, Levy MM. Evaluating the use of recombinant human activated protein C in adult severe sepsis: results of the Surviving Sepsis Campaign. Crit Care Med. 2012;40(5):1417-26.

317. Rimmer E, Kumar A, Doucette S, Marshall J, Dial S, Gurka D, et al. Activated protein C and septic shock: a propensity-matched cohort study*. Crit Care Med. 2012;40(11):2974-81.

318. Anger KE, Degrado JR, Greenwood BC, Cohen SA, Szumita PM. Evaluation of recombinant activated protein C for severe sepsis at a tertiary academic medical center. Ther Clin Risk Manag. 2013;9:277-84.

319. Angus DC, Laterre PF, Helterbrand J, Ely EW, Ball DE, Garg R, et al. The effect of drotrecogin alfa (activated) on long-term survival after severe sepsis. Crit Care Med. 2004;32(11):2199-206.

320. Man M, Close SL, Shaw AD, Bernard GR, Douglas IS, Kaner RJ, et al. Beyond single-marker analyses: mining whole genome scans for insights into treatment responses in severe sepsis. Pharmacogenomics J. 2013;13(3):218-26.

321. Sinha P, Kerchberger VE, Willmore A, Chambers J, Zhuo H, Abbott J, et al. Identifying molecular phenotypes in sepsis: an analysis of two prospective observational cohorts and secondary analysis of two randomised controlled trials. Lancet Respir Med. 2023;11(11):965-74.

322. Messori A, Vacca F, Vaiani M, Trippoli S. Antithrombin III in patients admitted to intensive care units: a multicenter observational study. Crit Care. 2002;6(5):447-51.

323. Wiedermann CJ, Hoffmann JN, Juers M, Ostermann H, Kienast J, Briegel J, et al. High-dose antithrombin III in the treatment of severe sepsis in patients with a high risk of death: efficacy and safety. Crit Care Med. 2006;34(2):285-92.

324. Kienast J, Juers M, Wiedermann CJ, Hoffmann JN, Ostermann H, Strauss R, et al. Treatment effects of high-dose antithrombin without concomitant heparin in patients with severe sepsis with or without disseminated intravascular coagulation. J Thromb Haemost. 2006;4(1):90-7.

325. Eid A, Wiedermann CJ, Kinasewitz GT. Early administration of high-dose antithrombin in severe sepsis: single center results from the KyberSept-trial. Anesth Analg. 2008;107(5):1633-8.

326. Moubarak P, Zilker S, Wolf H, Hofner B, Kneib T, Küchenhoff H, et al. Activity-guided antithrombin III therapy in severe surgical sepsis: efficacy and safety according to a retrospective data analysis. Shock. 2008;30(6):634-41.

327. Iba T, Saito D, Wada H, Asakura H. Efficacy and bleeding risk of antithrombin supplementation in septic disseminated intravascular coagulation: a prospective multicenter survey. Thromb Res. 2012;130(3):e129-33.

328. Sakamoto Y, Inoue S, Iwamura T, Yamashita T, Nakashima A, Nishimura Y, et al. Studies on therapeutic effects and pathological features of an antithrombin preparation in septic disseminated intravascular coagulation patients. Yonsei Med J. 2013;54(3):686-9.

329. Kawano S, Uchino S, Endo A, Saito K, Iwai K, Kase Y, Takinami M. Impact of withdrawing antithrombin III administration from management of septic patients with or without disseminated intravascular coagulation. Blood Coagul Fibrinolysis. 2014;25(8):795-800.

330. Tagami T, Matsui H, Horiguchi H, Fushimi K, Yasunaga H. Antithrombin and mortality in severe pneumonia patients with sepsis-associated disseminated intravascular coagulation: an observational nationwide study. J Thromb Haemost. 2014;12(9):1470-9.

331. Hayakawa M, Kudo D, Saito S, Uchino S, Yamakawa K, Iizuka Y, et al. Antithrombin Supplementation and Mortality in Sepsis-Induced Disseminated Intravascular Coagulation: A Multicenter Retrospective Observational Study. Shock. 2016;46(6):623-31.

332. Iba T, Gando S, Saitoh D, Wada H, Di Nisio M, Thachil J. Antithrombin supplementation and risk of bleeding in patients with sepsis-associated disseminated intravascular coagulation. Thromb Res. 2016;145:46-50.

333. Iba T, Gando S, Saitoh D, Ikeda T, Anan H, Oda S, et al. Efficacy and Bleeding Risk of Antithrombin Supplementation in Patients With Septic Disseminated Intravascular Coagulation: A Third Survey. Clin Appl Thromb Hemost. 2017;23(5):422-8.

334. Hayakawa M, Yamakawa K, Kudo D, Ono K. Optimal Antithrombin Activity Threshold for Initiating Antithrombin Supplementation in Patients With Sepsis-Induced Disseminated Intravascular Coagulation: A Multicenter Retrospective Observational Study. Clin Appl Thromb Hemost. 2018;24(6):874-83.

335. Iba T, Tanigawa T, Wada H, Levy JH. The antithrombin activity recovery after substitution therapy is associated with improved 28-day mortality in patients with sepsis-associated disseminated intravascular coagulation. Thromb J. 2023;21(1):112.

336. Kanda N, Ohbe H, Nakamura K. Effects of Antithrombin on Persistent Inflammation, Immunosuppression, and Catabolism Syndrome among Patients with Sepsis-Induced Disseminated Intravascular Coagulation. J Clin Med. 2023;12(11).

337. Yasuda N, Goto K, Ohchi Y, Abe T, Koga H, Kitano T. The efficacy and safety of antithrombin and recombinant human thrombomodulin combination therapy in patients with severe sepsis and disseminated intravascular coagulation. J Crit Care. 2016;36:29-34.

338. Iba T, Hagiwara A, Saitoh D, Anan H, Ueki Y, Sato K, Gando S. Effects of combination therapy using antithrombin and thrombomodulin for sepsis-associated disseminated intravascular coagulation. Ann Intensive Care. 2017;7(1):110.

339. Tanaka K, Takeba J, Matsumoto H, Ohshita M, Annen S, Moriyama N, et al. Anticoagulation Therapy Using rh-Thrombomodulin and/or Antithrombin III Agent is Associated With Reduction in in-Hospital Mortality in Septic Disseminated Intravascular Coagulation: A Nationwide Registry Study. Shock. 2019;51(6):713-7.

340. Suzuki J, Sasabuchi Y, Hatakeyama S, Matsui H, Sasahara T, Morisawa Y, et al. The effect of antithrombin added to recombinant human-soluble thrombomodulin for severe community-acquired pneumonia-associated disseminated intravascular coagulation: a retrospective cohort study using a nationwide inpatient database. J Intensive Care. 2020;8:8.

341. Umegaki T, Kunisawa S, Nishimoto K, Kamibayashi T, Imanaka Y. Effectiveness of combined antithrombin and thrombomodulin therapy on in-hospital mortality in mechanically ventilated septic patients with disseminated intravascular coagulation. Scientific Reports. 2020;10(1):4874.

342. Murao A, Kato T, Yamane T, Honda G, Eguchi Y. Benefit Profile of Thrombomodulin Alfa Combined with Antithrombin Concentrate in Patients with Sepsis-Induced Disseminated Intravascular Coagulation. Clin Appl Thromb Hemost. 2022;28:10760296221077096.

343. Wada T, Yamakawa K, Kabata D, Abe T, Ogura H, Shiraishi A, et al. Age-related differences in the survival benefit of the administration of antithrombin, recombinant human thrombomodulin, or their combination in sepsis. Sci Rep. 2022;12(1):9304.

344. Wada T, Yamakawa K, Kabata D, Abe T, Fujishima S, Kushimoto S, et al. Sepsis-related coagulopathy treatment based on the disseminated intravascular coagulation diagnostic criteria: a post-hoc analysis of a prospective multicenter observational study. J Intensive Care. 2023;11(1):8.

345. Wada H, Kawasugi K, Honda G, Kawano N, Uchiyama T, Madoiwa S, et al. Sepsis-Associated DIC with Decreased Levels of Antithrombin and Fibrinogen is the Target for Combination Therapy with Thrombomodulin Alfa and Antithrombin. TH Open. 2023;7(1):e65-e75.

346. Tschaikowsky K, Sägner S, Lehnert N, Kaul M, Ritter J. Endothelin in septic patients: effects on cardiovascular and renal function and its relationship to proinflammatory cytokines. Crit Care Med. 2000;28(6):1854-60.

347. Zarychanski R, Doucette S, Fergusson D, Roberts D, Houston DS, Sharma S, et al. Early intravenous unfractionated heparin and mortality in septic shock. Crit Care Med. 2008;36(11):2973-9.

348. Peng JC, Nie F, Li YJ, Xu QY, Xing SP, Li W, Gao Y. Favorable Outcomes of Anticoagulation With Unfractioned Heparin in Sepsis-Induced Coagulopathy: A Retrospective Analysis of MIMIC-III Database. Front Med (Lausanne). 2021;8:773339.

349. Huang JJ, Zou ZY, Zhou ZP, Liu Y, Yang ZJ, Zhang JJ, et al. Effectiveness of early heparin therapy on outcomes in critically ill patients with sepsis-induced coagulopathy. Front Pharmacol. 2023;14:1173893.

350. Staudinger T, Presterl E, Graninger W, Locker GJ, Knapp S, Laczika K, et al. Influence of pentoxifylline on cytokine levels and inflammatory parameters in septic shock. Intensive Care Med. 1996;22(9):888-93.

351. Yamakawa K, Umemura Y, Hayakawa M, Kudo D, Sanui M, Takahashi H, et al. Benefit profile of anticoagulant therapy in sepsis: a nationwide multicentre registry in Japan. Crit Care. 2016;20(1):229.

352. Kudo D, Hayakawa M, Ono K, Yamakawa K. Impact of non-anticoagulant therapy on patients with sepsis-induced disseminated intravascular coagulation: A multicenter, case-control study. Thromb Res. 2018;163:22-9.

353. Yamakawa K, Gando S, Ogura H, Umemura Y, Kabata D, Shintani A, et al. Identifying Sepsis Populations Benefitting from Anticoagulant Therapy: A Prospective Cohort Study Incorporating a Restricted Cubic Spline Regression Model. Thromb Haemost. 2019;119(11):1740-51.

354. Kudo D, Hayakawa M, Iijima H, Yamakawa K, Saito S, Uchino S, et al. The Treatment Intensity of Anticoagulant Therapy for Patients With Sepsis-Induced Disseminated Intravascular Coagulation and Outcomes: A Multicenter Cohort Study. Clin Appl Thromb Hemost. 2019;25:1076029619839154.

355. Kato T, Sakai T, Kato M, Hagihara M, Hasegawa T, Matsuura K, Nakagawa T. Recombinant human soluble thrombomodulin administration improves sepsis-induced disseminated intravascular coagulation and mortality: a retrospective cohort study. Thromb J. 2013;11(1):3.

356. Tagami T, Matsui H, Horiguchi H, Fushimi K, Yasunaga H. Recombinant human soluble thrombomodulin and mortality in severe pneumonia patients with sepsis-associated disseminated intravascular coagulation: an observational nationwide study. J Thromb Haemost. 2015;13(1):31-40.

357. Yoshimura J, Yamakawa K, Ogura H, Umemura Y, Takahashi H, Morikawa M, et al. Benefit profile of recombinant human soluble thrombomodulin in sepsis-induced disseminated intravascular coagulation: a multicenter propensity score analysis. Crit Care. 2015;19(1):78.

358. Hayakawa M, Yamakawa K, Saito S, Uchino S, Kudo D, Iizuka Y, et al. Recombinant human soluble thrombomodulin and mortality in sepsis-induced disseminated intravascular coagulation. A multicentre retrospective study. Thromb Haemost. 2016;115(6):1157-66.

359. Yoshihiro S, Sakuraya M, Hayakawa M, Ono K, Hirata A, Takaba A, et al. Recombinant Human-Soluble Thrombomodulin Contributes to Reduced Mortality in Sepsis Patients With Severe Respiratory Failure: A Retrospective Observational Study Using a Multicenter Dataset. Shock. 2019;51(2):174-9.

360. Levi M, Vincent JL, Tanaka K, Radford AH, Kayanoki T, Fineberg DA, et al. Effect of a Recombinant Human Soluble Thrombomodulin on Baseline Coagulation Biomarker Levels and Mortality Outcome in Patients With Sepsis-Associated Coagulopathy. Crit Care Med. 2020;48(8):1140-7.

361. Kudo D, Goto T, Uchimido R, Hayakawa M, Yamakawa K, Abe T, et al. Coagulation phenotypes in sepsis and effects of recombinant human thrombomodulin: an analysis of three multicentre observational studies. Critical Care. 2021;25(1):114.

362. Kotake K, Hongo T, Tahira A, Niimi N, Haisa I, Kawakami Y. Factors Determining the Efficacy of Recombinant Human Thrombomodulin in the Treatment of Sepsis-Induced Disseminated Intravascular Coagulation. Biol Pharm Bull. 2021;44(5):605-10.

363. François B, Fiancette M, Helms J, Mercier E, Lascarrou JB, Kayanoki T, et al. Efficacy and safety of human soluble thrombomodulin (ART-123) for treatment of patients in France with sepsis-associated coagulopathy: post hoc analysis of SCARLET. Ann Intensive Care. 2021;11(1):53.

364. Nishita Y, Taga M, Sakurai M, Iinuma Y, Masauji T. Prognostic factors in patients with septic disseminated intravascular coagulation treated with thrombomodulin: the effect of reduced thrombomodulin dose; a single-center, retrospective, observational study. Journal of Pharmaceutical Health Care and Sciences. 2022;8(1):32.

365. Imaura M, Katagiri F, Nagase S, Hatoyama-Tanaka S, Takahashi H, Takayanagi R, et al. OPTIMAL PLASMA CONCENTRATION OF THROMBOMODULIN ALFA TO TREAT SEPSIS-INDUCED DISSEMINATED INTRAVASCULAR COAGULATION. Shock. 2023;60(2):221-6.

366. Ogura T, Eguchi T, Nakahara K, Kanno Y, Omoto S, Itonaga M, et al. Clinical impact of recombinant thrombomodulin administration on disseminated intravascular coagulation due to severe acute cholangitis (Recover-AC study). J Hepatobiliary Pancreat Sci. 2023;30(2):221-8.

367. Liu Y, Jin G, Sun J, Wang X, Guo L. Recombinant human thrombopoietin in critically ill patients with sepsis-associated thrombocytopenia: A clinical study. Int J Infect Dis. 2020;98:144-9.

368. Wu Q, Ren J, Wu X, Wang G, Gu G, Liu S, et al. Recombinant human thrombopoietin improves platelet counts and reduces platelet transfusion possibility among patients with severe sepsis and thrombocytopenia: a prospective study. J Crit Care. 2014;29(3):362-6.

369. Dhainaut JF, Yan SB, Joyce DE, Pettilä V, Basson B, Brandt JT, et al. Treatment effects of drotrecogin alfa (activated) in patients with severe sepsis with or without overt disseminated intravascular coagulation. J Thromb Haemost. 2004;2(11):1924-33.

370. Vincent JL, Bernard GR, Beale R, Doig C, Putensen C, Dhainaut JF, et al. Drotrecogin alfa (activated) treatment in severe sepsis from the global open-label trial ENHANCE: further evidence for survival and safety and implications for early treatment. Crit Care Med. 2005;33(10):2266-77.

371. Pastores SM, Shaw A, Williams MD, Mongan E, Alicea M, Halpern NA. A safety evaluation of drotrecogin alfa (activated) in hematopoietic stem cell transplant patients with severe sepsis: lessons in clinical research. Bone Marrow Transplant. 2005;36(8):721-4.

372. De Backer D, Verdant C, Chierego M, Koch M, Gullo A, Vincent JL. Effects of drotrecogin alfa activated on microcirculatory alterations in patients with severe sepsis. Crit Care Med. 2006;34(7):1918-24.

373. Chapital AD, Yu M, Ho HC, Wang J, Koss W, Takanishi DM, Jr. Using transcutaneous oxygen pressure measurements as selection criteria for activated protein C use. J Trauma. 2008;65(1):30-3.

374. Decruyenaere J, De Backer D, Spapen H, Laterre PF, Raemaekers J, Rogiers P, et al. 90-day follow-up of patients treated with Drotrecogin Alfa (activated) for severe sepsis: a Belgian open label study. Acta Clin Belg. 2009;64(1):16-22.

375. Crivellari M, Silvetti S, Gerli C, Landoni G, Franco A, Bove T, et al. Protein C zymogen in adults with severe sepsis or septic shock. Med Intensiva. 2014;38(5):278-82.

376. Ogawa Y, Yamakawa K, Ogura H, Kiguchi T, Mohri T, Nakamori Y, et al. Recombinant human soluble thrombomodulin improves mortality and respiratory dysfunction in patients with severe sepsis. J Trauma Acute Care Surg. 2012;72(5):1150-7.

377. Yamakawa K, Fujimi S, Mohri T, Matsuda H, Nakamori Y, Hirose T, et al. Treatment effects of recombinant human soluble thrombomodulin in patients with severe sepsis: a historical control study. Crit Care. 2011;15(3):R123.

378. Boldt J, Papsdorf M, Kumle B, Piper S, Hempelmann G. Influence of angiotensin-converting enzyme inhibitor enalaprilat on endothelial-derived substances in the critically ill. Crit Care Med. 1998;26(10):1663-70.

379. Bernard GR, Ely EW, Wright TJ, Fraiz J, Stasek JE, Jr., Russell JA, et al. Safety and dose relationship of recombinant human activated protein C for coagulopathy in severe sepsis. Crit Care Med. 2001;29(11):2051-9.

380. Bernard GR, Vincent JL, Laterre PF, LaRosa SP, Dhainaut JF, Lopez-Rodriguez A, et al. Efficacy and safety of recombinant human activated protein C for severe sepsis. N Engl J Med. 2001;344(10):699-709.

381. Macias WL, Dhainaut JF, Yan SC, Helterbrand JD, Seger M, Johnson G, 3rd, Small DS. Pharmacokinetic-pharmacodynamic analysis of drotrecogin alfa (activated) in patients with severe sepsis. Clin Pharmacol Ther. 2002;72(4):391-402.

382. Vincent JL, Angus DC, Artigas A, Kalil A, Basson BR, Jamal HH, et al. Effects of drotrecogin alfa (activated) on organ dysfunction in the PROWESS trial. Crit Care Med. 2003;31(3):834-40.

383. Angus DC, Linde-Zwirble WT, Clermont G, Ball DE, Basson BR, Ely EW, et al. Cost-effectiveness of drotrecogin alfa (activated) in the treatment of severe sepsis. Crit Care Med. 2003;31(1):1-11.

384. Dhainaut JF, Laterre PF, Janes JM, Bernard GR, Artigas A, Bakker J, et al. Drotrecogin alfa (activated) in the treatment of severe sepsis patients with multiple-organ dysfunction: data from the PROWESS trial. Intensive Care Med. 2003;29(6):894-903.

385. Dhainaut JF, Yan SB, Margolis BD, Lorente JA, Russell JA, Freebairn RC, et al. Drotrecogin alfa (activated) (recombinant human activated protein C) reduces host coagulopathy response in patients with severe sepsis. Thromb Haemost. 2003;90(4):642-53.

386. Abraham E, Laterre PF, Garg R, Levy H, Talwar D, Trzaskoma BL, et al. Drotrecogin alfa (activated) for adults with severe sepsis and a low risk of death. N Engl J Med. 2005;353(13):1332-41.

387. Laterre PF, Abraham E, Janes JM, Trzaskoma BL, Correll NL, Booth FV. ADDRESS (ADministration of DRotrecogin alfa [activated] in Early stage Severe Sepsis) long-term follow-up: one-year safety and efficacy evaluation. Crit Care Med. 2007;35(6):1457-63.

388. Levi M, Levy M, Williams MD, Douglas I, Artigas A, Antonelli M, et al. Prophylactic heparin in patients with severe sepsis treated with drotrecogin alfa (activated). Am J Respir Crit Care Med. 2007;176(5):483-90.

389. Dhainaut JF, Antonelli M, Wright P, Desachy A, Reignier J, Lavoue S, et al. Extended drotrecogin alfa (activated) treatment in patients with prolonged septic shock. Intensive Care Med. 2009;35(7):1187-95.

390. Shorr AF, Janes JM, Artigas A, Tenhunen J, Wyncoll DL, Mercier E, et al. Randomized trial evaluating serial protein C levels in severe sepsis patients treated with variable doses of drotrecogin alfa (activated). Crit Care. 2010;14(6):R229.

391. Ranieri VM, Thompson BT, Barie PS, Dhainaut JF, Douglas IS, Finfer S, et al. Drotrecogin alfa (activated) in adults with septic shock. N Engl J Med. 2012;366(22):2055-64.

392. Pappalardo F, Crivellari M, Di Prima AL, Agracheva N, Celinska-Spodar M, Lembo R, et al. Protein C zymogen in severe sepsis: a double-blinded, placebo-controlled, randomized study. Intensive Care Med. 2016;42(11):1706-14.

393. Laterre PF, Pickkers P, Marx G, Wittebole X, Meziani F, Dugernier T, et al. Safety and tolerability of non-neutralizing adrenomedullin antibody adrecizumab (HAM8101) in septic shock patients: the AdrenOSS-2 phase 2a biomarker-guided trial. Intensive Care Med. 2021;47(11):1284-94.

394. Fourrier F, Chopin C, Huart JJ, Runge I, Caron C, Goudemand J. Double-blind, placebo-controlled trial of antithrombin III concentrates in septic shock with disseminated intravascular coagulation. Chest. 1993;104(3):882-8.

395. Inthorn D, Hoffmann JN, Hartl WH, Mühlbayer D, Jochum M. Antithrombin III supplementation in severe sepsis: beneficial effects on organ dysfunction. Shock. 1997;8(5):328-34.

396. Eisele B, Lamy M, Thijs LG, Keinecke HO, Schuster HP, Matthias FR, et al. Antithrombin III in patients with severe sepsis. A randomized, placebo-controlled, double-blind multicenter trial plus a meta-analysis on all randomized, placebo-controlled, double-blind trials with antithrombin III in severe sepsis. Intensive Care Med. 1998;24(7):663-72.

397. Inthorn D, Hoffmann JN, Hartl WH, Mühlbayer D, Jochum M. Effect of antithrombin III supplementation on inflammatory response in patients with severe sepsis. Shock. 1998;10(2):90-6.

398. Baudo F, Caimi TM, de Cataldo F, Ravizza A, Arlati S, Casella G, et al. Antithrombin III (ATIII) replacement therapy in patients with sepsis and/or postsurgical complications: a controlled double-blind, randomized, multicenter study. Intensive Care Med. 1998;24(4):336-42.

399. Warren BL, Eid A, Singer P, Pillay SS, Carl P, Novak I, et al. Caring for the critically ill patient. High-dose antithrombin III in severe sepsis: a randomized controlled trial. Jama. 2001;286(15):1869-78.

400. Rublee D, Opal SM, Schramm W, Keinecke HO, Knaub S. Quality of life effects of antithrombin III in sepsis survivors: results from the KyberSept trial [ISRCTN22931023]. Crit Care. 2002;6(4):349-56.

401. Hoffmann JN, Mühlbayer D, Jochum M, Inthorn D. Effect of long-term and high-dose antithrombin supplementation on coagulation and fibrinolysis in patients with severe sepsis. Crit Care Med. 2004;32(9):1851-9.

402. Gonano C, Sitzwohl C, Meitner E, Weinstabl C, Kettner SC. Four-day antithrombin therapy does not seem to attenuate hypercoagulability in patients suffering from sepsis. Crit Care. 2006;10(6):R160.

403. Gando S, Saitoh D, Ishikura H, Ueyama M, Otomo Y, Oda S, et al. A randomized, controlled, multicenter trial of the effects of antithrombin on disseminated intravascular coagulation in patients with sepsis. Critical Care. 2013;17(6):R297.

404. Hoffmann JN, Wiedermann CJ, Juers M, Ostermann H, Kienast J, Briegel J, et al. Benefit/risk profile of high-dose antithrombin in patients with severe sepsis treated with and without concomitant heparin. Thromb Haemost. 2006;95(5):850-6.

405. Jaimes F, De La Rosa G, Morales C, Fortich F, Arango C, Aguirre D, Muñoz A. Unfractioned heparin for treatment of sepsis: A randomized clinical trial (The HETRASE Study). Crit Care Med. 2009;37(4):1185-96.

406. Liu XL, Wang XZ, Liu XX, Hao D, Jaladat Y, Lu F, et al. Low-dose heparin as treatment for early disseminated intravascular coagulation during sepsis: A prospective clinical study. Exp Ther Med. 2014;7(3):604-8.

407. Yang C, Chen X, Zheng D, Chen L, Ding Y, Xie W. Impacts of early anticoagulant therapy on tissue perfusion in patients with sepsis. Int J Clin Exp Med. 2016;9(10):20074-9.

408. Nouri M, Ahmadi A, Etezadi F, Barzegar E, Mojtahedzadeh M. Comparison of the Effects of Subcutaneous Versus Continuous Infusion of Heparin on Key Inflammatory Parameters Following Sepsis. Anesth Pain Med. 2016;6(2):e33780.

409. Levy M, Levi M, Williams MD, Antonelli M, Wang D, Mignini MA. Comprehensive safety analysis of concomitant drotrecogin alfa (activated) and prophylactic heparin use in patients with severe sepsis. Intensive Care Med. 2009;35(7):1196-203.

410. Chen TT, Lv JJ, Chen L, Li M, Liu LP. Heparanase inhibition leads to improvement in patients with acute gastrointestinal injuries induced by sepsis. World J Gastroenterol. 2023;29(35):5154-65.

411. Berthelsen RE, Ostrowski SR, Bestle MH, Johansson PI. Co-administration of iloprost and eptifibatide in septic shock (CO-ILEPSS)-a randomised, controlled, double-blind investigator-initiated trial investigating safety and efficacy. Crit Care. 2019;23(1):301.

412. Boldt J, Heesen M, MÜLler M, Linke LC, Hempelmann G. Interleukin-10 plasma levels in the critically ill. Clinical Intensive Care. 1995;6(4):153-8.

413. Castañon-Gonzalez JA, Eid-Lidt G, Wacher N, Gallegos-Perez H, Miranda-Ruiz R. Pentoxifylline and oxygen consumption in severe sepsis--a preliminary report. Acta Anaesthesiol Scand Suppl. 1995;107:219-22.

414. Zeni F, Pain P, Vindimian M, Gay JP, Gery P, Bertrand M, et al. Effects of pentoxifylline on circulating cytokine concentrations and hemodynamics in patients with septic shock: results from a double-blind, randomized, placebo-controlled study. Crit Care Med. 1996;24(2):207-14.

415. Boldt J, Müller M, Heesen M, Heyn S, Hempelmann G. Does long-term continuous administration of pentoxifylline affect platelet function in the critically ill patient? Intensive Care Med. 1996;22(7):644-50.

416. Boldt J, Muller M, Heesen M, Neumann K, Hempelmann GG. Influence of different volume therapies and pentoxifylline infusion on circulating soluble adhesion molecules in critically ill patients. Crit Care Med. 1996;24(3):385-91.

417. Boldt J, Müller M, Heyn S, Welters I, Hempelmann G. Influence of long-term continuous intravenous administration of pentoxifylline on endothelial-related coagulation in critically ill patients. Crit Care Med. 1996;24(6):940-6.

418. Staubach KH, Schröder J, Stüber F, Gehrke K, Traumann E, Zabel P. Effect of pentoxifylline in severe sepsis: results of a randomized, double-blind, placebo-controlled study. Arch Surg. 1998;133(1):94-100.

419. Elgendy HA, Ibrahim HM, Hasan BEE, Elkawe ASA. Effect of Pentoxifylline on Organ Dysfunction and Mortality in Severe Sepsis. The Open Anesthesia Journal. 2020;14(1):14-21.

420. Vincent JL, Spapen H, Bakker J, Webster NR, Curtis L. Phase II multicenter clinical study of the platelet-activating factor receptor antagonist BB-882 in the treatment of sepsis. Crit Care Med. 2000;28(3):638-42.

421. Poeze M, Froon AH, Ramsay G, Buurman WA, Greve JW. Decreased organ failure in patients with severe SIRS and septic shock treated with the platelet-activating factor antagonist TCV-309: a prospective, multicenter, double-blind, randomized phase II trial. TCV-309 Septic Shock Study Group. Shock. 2000;14(4):421-8.

422. Suputtamongkol Y, Intaranongpai S, Smith MD, Angus B, Chaowagul W, Permpikul C, et al. A double-blind placebo-controlled study of an infusion of lexipafant (Platelet-activating factor receptor antagonist) in patients with severe sepsis. Antimicrob Agents Chemother. 2000;44(3):693-6.

423. Albrecht DM, van Ackern K, Bender HJ, Hof H, Kox W, Victor N, et al. Efficacy and Safety of the Platelet-Activating Factor Receptor Antagonist BN 52021 (Ginkgolide B) in Patients with Severe Sepsis : A Randomised, Double-Blind, Placebo-Controlled, Multicentre Trial. Clin Drug Investig. 2004;24(3):137-47.

424. Vincent JL, Ramesh MK, Ernest D, LaRosa SP, Pachl J, Aikawa N, et al. A randomized, double-blind, placebo-controlled, Phase 2b study to evaluate the safety and efficacy of recombinant human soluble thrombomodulin, ART-123, in patients with sepsis and suspected disseminated intravascular coagulation. Crit Care Med. 2013;41(9):2069-79.

425. Eguchi Y, Gando S, Ishikura H, Saitoh D, Mimuro J, Takahashi H, et al. Post-marketing surveillance data of thrombomodulin alfa: sub-analysis in patients with sepsis-induced disseminated intravascular coagulation. Journal of Intensive Care. 2014;2(1):30.

426. Hagiwara A, Tanaka N, Uemura T, Matsuda W, Kimura A. Can recombinant human thrombomodulin increase survival among patients with severe septic-induced disseminated intravascular coagulation: a single-centre, open-label, randomised controlled trial. BMJ Open. 2016;6(12):e012850.

427. Vincent JL, Francois B, Zabolotskikh I, Daga MK, Lascarrou JB, Kirov MY, et al. Effect of a Recombinant Human Soluble Thrombomodulin on Mortality in Patients With Sepsis-Associated Coagulopathy: The SCARLET Randomized Clinical Trial. Jama. 2019;321(20):1993-2002.

428. Mori S, Ai T, Sera T, Ochiai K, Otomo Y. Human Soluble Recombinant Thrombomodulin, ART-123, Resolved Early Phase Coagulopathies, but Did Not Significantly Alter the 28 Day Outcome in the Treatment of DIC Associated with Infectious Systemic Inflammatory Response Syndromes. J Clin Med. 2019;8(10).

429. Abraham E, Reinhart K, Opal S, Demeyer I, Doig C, Rodriguez AL, et al. Efficacy and safety of tifacogin (recombinant tissue factor pathway inhibitor) in severe sepsis: a randomized controlled trial. Jama. 2003;290(2):238-47.

430. Abraham E, Reinhart K, Svoboda P, Seibert A, Olthoff D, Dal Nogare A, et al. Assessment of the safety of recombinant tissue factor pathway inhibitor in patients with severe sepsis: a multicenter, randomized, placebo-controlled, single-blind, dose escalation study. Crit Care Med. 2001;29(11):2081-9.

431. Wunderink RG, Laterre PF, Francois B, Perrotin D, Artigas A, Vidal LO, et al. Recombinant tissue factor pathway inhibitor in severe community-acquired pneumonia: a randomized trial. Am J Respir Crit Care Med. 2011;183(11):1561-8.

432. Morris PE, Steingrub JS, Huang BY, Tang S, Liu PM, Rhode PR, Wong HC. A phase I study evaluating the pharmacokinetics, safety and tolerability of an antibody-based tissue factor antagonist in subjects with acute lung injury or acute respiratory distress syndrome. BMC Pulm Med. 2012;12:5.

433. Eggers V, Fügener K, Hein OV, Rommelspacher H, Heyes MP, Kox WJ, Spies CD. Antibiotic-mediated release of tumour necrosis factor alpha and norharman in patients with hospital-acquired pneumonia and septic encephalopathy. Intensive Care Med. 2004;30(8):1544-51.

434. Restrepo MI, Mortensen EM, Waterer GW, Wunderink RG, Coalson JJ, Anzueto A. Impact of macrolide therapy on mortality for patients with severe sepsis due to pneumonia. Eur Respir J. 2009;33(1):153-9.

435. Miano TA, Hennessy S, Yang W, Dunn TG, Weisman AR, Oniyide O, et al. Association of vancomycin plus piperacillin-tazobactam with early changes in creatinine versus cystatin C in critically ill adults: a prospective cohort study. Intensive Care Med. 2022;48(9):1144-55.

436. Behal ML, Nguyen JL, Li X, Feola DJ, Neyra JA, Flannery AH. Azithromycin and Major Adverse Kidney Events in Critically Ill Patients With Sepsis-Associated Acute Kidney Injury. Shock. 2022;57(4):479-85.

437. Pang S, Song Y, Yang J, Li S, He Y, Luo G. Efficacy of Etimicin Sulfate Combined with Cefotaxime Sodium in the Treatment of Patients with Septic Shock and Effect on Serum Inflammatory Factor Levels and Immune Function. Evid Based Complement Alternat Med. 2021;2021:8583118.

438. Fernández J, Escorsell A, Zabalza M, Felipe V, Navasa M, Mas A, et al. Adrenal insufficiency in patients with cirrhosis and septic shock: Effect of treatment with hydrocortisone on survival. Hepatology. 2006;44(5):1288-95.

439. Annane D, Sébille V, Bellissant E. Effect of low doses of corticosteroids in septic shock patients with or without early acute respiratory distress syndrome. Crit Care Med. 2006;34(1):22-30.

440. Raurich JM, Llompart-Pou JA, Ibáñez J, Frontera G, Pérez O, García L, Ayestarán JI. Low-dose steroid therapy does not affect hemodynamic response in septic shock patients. J Crit Care. 2007;22(4):324-9.

441. Yu TJ, Liu YC, Yu CC, Tseng JC, Hua CC, Wu HP. Comparing hydrocortisone and methylprednisolone in patients with septic shock. Adv Ther. 2009;26(7):728-35.

442. Park HY, Suh GY, Song JU, Yoo H, Jo IJ, Shin TG, et al. Early initiation of low-dose corticosteroid therapy in the management of septic shock: a retrospective observational study. Crit Care. 2012;16(1):R3.

443. Funk D, Doucette S, Pisipati A, Dodek P, Marshall JC, Kumar A. Low-dose corticosteroid treatment in septic shock: a propensity-matching study. Crit Care Med. 2014;42(11):2333-41.

444. Antcliffe DB, Burnham KL, Al-Beidh F, Santhakumaran S, Brett SJ, Hinds CJ, et al. Transcriptomic Signatures in Sepsis and a Differential Response to Steroids. From the VANISH Randomized Trial. Am J Respir Crit Care Med. 2019;199(8):980-6.

445. Venkatesh B, Finfer S, Cohen J, Rajbhandari D, Arabi Y, Bellomo R, et al. Hydrocortisone Compared with Placebo in Patients with Septic Shock Satisfying the Sepsis-3 Diagnostic Criteria and APROCCHSS Study Inclusion Criteria: A Post Hoc Analysis of the ADRENAL Trial. Anesthesiology. 2019;131(6):1292-300.

446. Wong HR, Hart KW, Lindsell CJ, Sweeney TE. External Corroboration That Corticosteroids May Be Harmful to Septic Shock Endotype A Patients. Crit Care Med. 2021;49(1):e98-e101.

447. Cohen J, Bellomo R, Billot L, Burrell LM, Evans DM, Finfer S, et al. Plasma Cortisol, Aldosterone, and Ascorbic Acid Concentrations in Patients with Septic Shock Do Not Predict Treatment Effect of Hydrocortisone on Mortality. A Nested Cohort Study. Am J Respir Crit Care Med. 2020;202(5):700-7.

448. McDonnell E, Collins R, Hernandez M, Brown ART. Effect of hydrocortisone versus methylprednisolone on clinical outcomes in oncology patients with septic shock. J Oncol Pharm Pract. 2021;27(1):54-62.

449. Pirracchio R, Hubbard A, Sprung CL, Chevret S, Annane D. Assessment of Machine Learning to Estimate the Individual Treatment Effect of Corticosteroids in Septic Shock. JAMA Netw Open. 2020;3(12):e2029050.

450. König R, Kolte A, Ahlers O, Oswald M, Krauss V, Roell D, et al. Use of IFNγ/IL10 Ratio for Stratification of Hydrocortisone Therapy in Patients With Septic Shock. Front Immunol. 2021;12:607217.

451. Cohen J, Blumenthal A, Cuellar-Partida G, Evans DM, Finfer S, Li Q, et al. The relationship between adrenocortical candidate gene expression and clinical response to hydrocortisone in patients with septic shock. Intensive Care Med. 2021;47(9):974-83.

452. Ragoonanan D, Allen B, Cannon C, Rottman-Pietrzak K, Bello A. Comparison of Early Versus Late Initiation of Hydrocortisone in Patients With Septic Shock in the ICU Setting. Ann Pharmacother. 2022;56(3):264-70.

453. Rauf-ul-Hassan M, Aslam S, Waseem M, Bashir I, Naz S, Iqbal A. Effect of hydrocortisone on outcome of septic shock patients. A prospective observational study in a tertiary care ICU. Pakistan Journal of Medical & Health Sciences. 2022;16(02):315-.

454. Schelling G, Stoll C, Kapfhammer HP, Rothenhäusler HB, Krauseneck T, Durst K, et al. The effect of stress doses of hydrocortisone during septic shock on posttraumatic stress disorder and health-related quality of life in survivors. Crit Care Med. 1999;27(12):2678-83.

455. Fuchs P, Bozkurt A, Johnen D, Smeets R, Groger A, Pallua N. Beneficial effect of corticosteroids in catecholamine-dependent septic burn patients. Burns. 2007;33(3):306-11.

456. Nazer L, AlNajjar T, Al-Shaer M, Rimawi D, Hawari F. Evaluating the effectiveness and safety of hydrocortisone therapy in cancer patients with septic shock. J Oncol Pharm Pract. 2015;21(4):274-9.

457. Wu HP, Shih CC, Chuang DY, Chen TH. Low-Dose Steroid Therapy Is Associated with Decreased IL-12 Production in PBMCs of Severe Septic Patients. Mediators Inflamm. 2016;2016:1796094.

458. Ceccato A, Cilloniz C, Ranzani OT, Menendez R, Agusti C, Gabarrus A, et al. Treatment with macrolides and glucocorticosteroids in severe community-acquired pneumonia: A post-hoc exploratory analysis of a randomized controlled trial. PLoS One. 2017;12(6):e0178022.

459. Thompson KJ, Young PJ, Venkatesh B, Cohen J, Finfer SR, Grattan S, et al. Long-term costs and cost-effectiveness of adjunctive corticosteroids for patients with septic shock in New Zealand. Aust Crit Care. 2022;35(3):241-50.

460. Sacha GL, Chen AY, Palm NM, Wang X, Li M, Duggal A. Clinical Utilization of Stress Dose Hydrocortisone in Adult Patients With Septic Shock: A Retrospective Observational Study at a Large Academic Medical Center. J Pharm Pract. 2023;36(3):606-13.

461. Lenney M, Kopp B, Erstad B. Effect of fixed-dose hydrocortisone on vasopressor dose and mean arterial pressure in obese and nonobese patients with septic shock. Am J Health Syst Pharm. 2022;79(Suppl 3):S94-s9.

462. Jochheim L, Jochheim D, Habenicht L, Herner A, Ulrich J, Wiessner J, et al. Adjunctive Hydrocortisone Improves Hemodynamics in Critically Ill Patients with Septic Shock: An Observational Study Using Transpulmonary Thermodilution. Journal of Intensive Care Medicine. 2023;38(8):717-26.

463. Dong Y, Heng G, Zhang J, Shen Y, Lan Z, Wei K, Jin W. Association between corticosteroid use and 28-day mortality in septic shock patients with gram-negative bacterial infection: a retrospective study. Front Med (Lausanne). 2023;10:1276181.

464. Lock AE, Gutierrez GC, Hand EO, Barthol CA, Attridge RL. Fludrocortisone Plus Hydrocortisone Versus Hydrocortisone Alone as Adjunctive Therapy in Septic Shock: A Retrospective Cohort Study. Ann Pharmacother. 2023;57(12):1375-88.

465. Cheng X, Fu Z, Liu Y, Zheng X, Hu T. Association of mortality with fludrocortisone addition to hydrocortisone treatment among septic shock patients: a propensity score matching analysis. Front Med (Lausanne). 2023;10:1190758.

466. Bologheanu R, Kapral L, Laxar D, Maleczek M, Dibiasi C, Zeiner S, et al. Development of a Reinforcement Learning Algorithm to Optimize Corticosteroid Therapy in Critically Ill Patients with Sepsis. J Clin Med. 2023;12(4).

467. Bosch NA, Teja B, Law AC, Pang B, Jafarzadeh SR, Walkey AJ. Comparative Effectiveness of Fludrocortisone and Hydrocortisone vs Hydrocortisone Alone Among Patients With Septic Shock. JAMA Intern Med. 2023;183(5):451-9.

468. Carabetta S, Allen B, Cannon C, Johnson T. Abrupt Discontinuation Versus Taper of Hydrocortisone in Patients With Septic Shock. Ann Pharmacother. 2023;57(4):375-81.

469. Li M, Noordam R, Winter EM, van Meurs M, Bouma HR, Arbous MS, et al. Hydrocortisone-associated death and hospital length of stay in patients with sepsis: A retrospective cohort of large-scale clinical care data. Biomed Pharmacother. 2024;170:115961.

470. Hellali R, Chelly Dagdia Z, Ktaish A, Zeitouni K, Annane D. Corticosteroid sensitivity detection in sepsis patients using a personalized data mining approach: A clinical investigation. Comput Methods Programs Biomed. 2024;245:108017.

471. Tagami T, Matsui H, Fushimi K, Yasunaga H. Low-dose corticosteroid treatment and mortality in refractory abdominal septic shock after emergency laparotomy. Ann Intensive Care. 2015;5(1):32.

472. Bonnin S, Radosevich JJ, Lee YG, Feldman JP, Barletta JF. Comparison of shock reversal with high or low dose hydrocortisone in intensive care unit patients with septic shock: A retrospective cohort study. J Crit Care. 2021;62:111-6.

473. Tagami T, Matsui H, Horiguchi H, Fushimi K, Yasunaga H. Low-dose corticosteroid use and mortality in severe community-acquired pneumonia patients. Eur Respir J. 2015;45(2):463-72.

474. Torres A, Ceccato A, Ferrer M, Gabarrus A, Sibila O, Cilloniz C, et al. Effect of Corticosteroids on C-Reactive Protein in Patients with Severe Community-Acquired Pneumonia and High Inflammatory Response: The Effect of Lymphopenia. J Clin Med. 2019;8(9).

475. Wu YP, Lauffenburger JC. Effectiveness of corticosteroids in patients with sepsis or septic shock using the new third international consensus definitions (Sepsis-3): A retrospective observational study. PLoS One. 2020;15(12):e0243149.

476. Schäfer ST, Gessner S, Scherag A, Rump K, Frey UH, Siffert W, et al. Hydrocortisone fails to abolish NF-κB1 protein nuclear translocation in deletion allele carriers of the NFKB1 promoter polymorphism (-94ins/delATTG) and is associated with increased 30-day mortality in septic shock. PLoS One. 2014;9(8):e104953.

477. Póvoa P, Salluh JI, Martinez ML, Guillamat-Prats R, Gallup D, Al-Khalidi HR, et al. Clinical impact of stress dose steroids in patients with septic shock: insights from the PROWESS-Shock trial. Crit Care. 2015;19(1):193.

478. Kang J, Han M, Hong SB, Lim CM, Koh Y, Huh JW. Effect of adjunctive corticosteroid on 28-day mortality in neutropenic patients with septic shock. Ann Hematol. 2019;98(10):2311-8.

479. Lu X, Wang X, Gao Y, Yu S, Zhao L, Zhang Z, et al. Efficacy and safety of corticosteroids for septic shock in immunocompromised patients: A cohort study from MIMIC. Am J Emerg Med. 2021;42:121-6.

480. Yao L, Rey DA, Bulgarelli L, Kast R, Osborn J, Van Ark E, et al. Gene Expression Scoring of Immune Activity Levels for Precision Use of Hydrocortisone in Vasodilatory Shock. Shock. 2022;57(3):384-91.

481. Rivas M, Motes A, Ismail A, Yang S, Sotello D, Arevalo M, et al. Characteristics and outcomes of patients with sepsis who had cortisol level measurements or received hydrocortisone during their intensive care unit management: A retrospective single center study. SAGE Open Med. 2023;11:20503121221146907.

482. Chang K, Harbin M, Shuster C, Griesdale DEG, Foster D, Sweet D, et al. Adding vitamin C to hydrocortisone lacks benefit in septic shock: a historical cohort study. Can J Anaesth. 2020;67(12):1798-805.

483. Coloretti I, Biagioni E, Venturelli S, Munari E, Tosi M, Roat E, et al. Adjunctive therapy with vitamin c and thiamine in patients treated with steroids for refractory septic shock: A propensity matched before-after, case-control study. J Crit Care. 2020;59:37-41.

484. Grossestreuer AV, Moskowitz A, Andersen LW, Holmberg MJ, Konacki V, Berg KM, et al. Effect of Ascorbic Acid, Corticosteroids, and Thiamine on Health-Related Quality of Life in Sepsis. Crit Care Explor. 2020;2(12):e0270.

485. Litwak JJ, Cho N, Nguyen HB, Moussavi K, Bushell T. Vitamin C, Hydrocortisone, and Thiamine for the Treatment of Severe Sepsis and Septic Shock: A Retrospective Analysis of Real-World Application. J Clin Med. 2019;8(4).

486. Long MT, Frommelt MA, Ries MP, Murray M, Osman F, Krause BM, Korv P. Early hydrocortisone, ascorbate and thiamine therapy for severe septic shock. Critical Care & Shock. 2020;23(1).

487. Marik PE, Khangoora V, Rivera R, Hooper MH, Catravas J. Hydrocortisone, Vitamin C, and Thiamine for the Treatment of Severe Sepsis and Septic Shock: A Retrospective Before-After Study. Chest. 2017;151(6):1229-38.

488. Mitchell AB, Ryan TE, Gillion AR, Wells LD, Muthiah MP. Vitamin C and Thiamine for Sepsis and Septic Shock. Am J Med. 2020;133(5):635-8.

489. Joseph MV, Mohamed ZU, Sathyapalan D, Moni M, Balachandran S, Ravindran GC, et al. Vitamin C, thiamin and hydrocortisone in septic shock: a retrospective analysis. International Journal of Pharmaceutical Sciences and Research. 2022;13(8).

490. Busse LW, Schaich CL, Chappell MC, McCurdy MT, Staples EM, Ten Lohuis CC, et al. Association of Active Renin Content With Mortality in Critically Ill Patients: A Post hoc Analysis of the Vitamin C, Thiamine, and Steroids in Sepsis Trial. Crit Care Med. 2023.

491. Boyle AJ, Ferris P, Bradbury I, Conlon J, Shankar-Hari M, Rogers AJ, et al. Baseline plasma IL-18 may predict simvastatin treatment response in patients with ARDS: a secondary analysis of the HARP-2 randomised clinical trial. Crit Care. 2022;26(1):164.

492. Miyamoto Y, Aso S, Iwagami M, Yasunaga H, Matsui H, Fushimi K, et al. Association Between IV Thiamine and Mortality in Patients With Septic Shock: A Nationwide Observational Study. Crit Care Med. 2020;48(8):1135-9.

493. Jung SY, Lee MT, Baek MS, Kim WY. Vitamin C for ≥ 5 days is associated with decreased hospital mortality in sepsis subgroups: a nationwide cohort study. Crit Care. 2022;26(1):3.

494. Hemilä H, Chalker E. Abrupt termination of vitamin C from ICU patients may increase mortality: secondary analysis of the LOVIT trial. Eur J Clin Nutr. 2023;77(4):490-4.

495. Suárez-de-la-Rica A, Croes B, Ciudad L, Vallejo I, Mújica J, Díaz-Almirón M, Maseda E. Vitamin C and thiamine for the treatment of refractory septic shock in surgical critically ill patients: a retrospective before-and-after study. Rev Esp Quimioter. 2023;36(2):187-92.

496. Park JE, Jo YH, Hwang SY, Kim WY, Ryoo SM, Jang DH, et al. Biomarker Analysis for Combination Therapy of Vitamin C and Thiamine in Septic Shock: A Post-Hoc Study of the ATESS Trial. Shock. 2022;57(1):81-7.

497. Li M, Tingting Z, Naixia H, Wenjing P. The Mechanism of Silver Sulfadiazine Regulating the Cellular Immune Activity of Burn Sepsis. Journal of Biological Regulators and Homeostatic Agents. 2023;37(10):5383-90.

498. Briegel J, Kellermann W, Forst H, Haller M, Bittl M, Hoffmann GE, et al. Low-dose hydrocortisone infusion attenuates the systemic inflammatory response syndrome. The Phospholipase A2 Study Group. Clin Investig. 1994;72(10):782-7.

499. Büchele GL, Silva E, Ospina-Tascón GA, Vincent JL, De Backer D. Effects of hydrocortisone on microcirculatory alterations in patients with septic shock. Crit Care Med. 2009;37(4):1341-7.

500. Zhao Y, Ding C. Effects of Hydrocortisone on Regulating Inflammation, Hemodynamic Stability, and Preventing Shock in Severe Sepsis Patients. Med Sci Monit. 2018;24:3612-9.

501. Lederer V. Betamethasone sodium phosphate injection: high-dose regimen in septic shock. Clin Ther. 1984;6(5):719-26.

502. Lavillegrand J-R, Raia L, Urbina T, Hariri G, Gabarre P, Bonny V, et al. Vitamin C improves microvascular reactivity and peripheral tissue perfusion in septic shock patients. Critical Care. 2022;26(1):25.

503. Zhang R, Liu H, Dai D, Ding X, Wang D, Wang Y, et al. Adjunctive sepsis therapy with aminophylline (STAP): a randomized controlled trial. Chin Med J (Engl). 2022;135(23):2843-50.

504. Prins JM, van Agtmael MA, Kuijper EJ, van Deventer SJ, Speelman P. Antibiotic-induced endotoxin release in patients with gram-negative urosepsis: a double-blind study comparing imipenem and ceftazidime. J Infect Dis. 1995;172(3):886-91.

505. Luchi M, Morrison DC, Opal S, Yoneda K, Slotman G, Chambers H, et al. A comparative trial of imipenem versus ceftazidime in the release of endotoxin and cytokine generation in patients with gram-negative urosepsis. Urosepsis Study Group. J Endotoxin Res. 2000;6(1):25-31.

506. Maskin B, Fontán PA, Spinedi EG, Gammella D, Badolati A. Evaluation of endotoxin release and cytokine production induced by antibiotics in patients with Gram-negative nosocomial pneumonia. Crit Care Med. 2002;30(2):349-54.

507. Giamarellos-Bourboulis EJ, Perdios J, Gargalianos P, Kosmidis J, Giamarellou H. Antimicrobial-induced endotoxaemia in patients with sepsis in the field of acute pyelonephritis. J Postgrad Med. 2003;49(2):118-22.

508. Gogos CA, Skoutelis A, Lekkou A, Drosou E, Starakis I, Marangos MN, Bassaris HP. Comparative effects of ciprofloxacin and ceftazidime on cytokine production in patients with severe sepsis caused by gram-negative bacteria. Antimicrob Agents Chemother. 2004;48(8):2793-8.

509. Giamarellos-Bourboulis EJ, Mylona V, Antonopoulou A, Tsangaris I, Koutelidakis I, Marioli A, et al. Effect of clarithromycin in patients with suspected Gram-negative sepsis: results of a randomized controlled trial. J Antimicrob Chemother. 2014;69(4):1111-8.

510. Giamarellos-Bourboulis EJ, Mega A, Pavleas I, Archontoulis N, Rigas K, Vernikos P, et al. Impact of carbapenem administration on systemic endotoxemia in patients with severe sepsis and Gram-negative bacteremia. J Chemother. 2006;18(5):502-6.

511. Giamarellos-Bourboulis EJ, Pechère JC, Routsi C, Plachouras D, Kollias S, Raftogiannis M, et al. Effect of clarithromycin in patients with sepsis and ventilator-associated pneumonia. Clin Infect Dis. 2008;46(8):1157-64.

512. Nukarinen E, Tervahartiala T, Valkonen M, Hynninen M, Kolho E, Pettilä V, et al. Targeting matrix metalloproteinases with intravenous doxycycline in severe sepsis--A randomised placebo-controlled pilot trial. Pharmacol Res. 2015;99:44-51.

513. Spyridaki A, Raftogiannis M, Antonopoulou A, Tsaganos T, Routsi C, Baziaka F, et al. Effect of clarithromycin in inflammatory markers of patients with ventilator-associated pneumonia and sepsis caused by Gram-negative bacteria: results from a randomized clinical study. Antimicrob Agents Chemother. 2012;56(7):3819-25.

514. Tsaganos T, Raftogiannis M, Pratikaki M, Christodoulou S, Kotanidou A, Papadomichelakis E, et al. Clarithromycin Leads to Long-Term Survival and Cost Benefit in Ventilator-Associated Pneumonia and Sepsis. Antimicrob Agents Chemother. 2016;60(6):3640-6.

515. Karakike E, Scicluna BP, Roumpoutsou M, Mitrou I, Karampela N, Karageorgos A, et al. Effect of intravenous clarithromycin in patients with sepsis, respiratory and multiple organ dysfunction syndrome: a randomized clinical trial. Crit Care. 2022;26(1):183.

516. Wang Y, Wang, B., Ma, L., Zhao, X., Qiao, Y., Zhang, X. Effect of hydrocortisone in a low dosage on apoptosis of T lymphocyte subset in treatment of sepsis. Acta medica mediterranea. 2019:35: 2653.

517. Annane D, Renault A, Brun-Buisson C, Megarbane B, Quenot JP, Siami S, et al. Hydrocortisone plus Fludrocortisone for Adults with Septic Shock. N Engl J Med. 2018;378(9):809-18.

518. Arabi YM, Aljumah A, Dabbagh O, Tamim HM, Rishu AH, Al-Abdulkareem A, et al. Low-dose hydrocortisone in patients with cirrhosis and septic shock: a randomized controlled trial. Cmaj. 2010;182(18):1971-7.

519. Hammond NE, Finfer SR, Li Q, Taylor C, Cohen J, Arabi Y, et al. Health-related quality of life in survivors of septic shock: 6-month follow-up from the ADRENAL trial. Intensive Care Med. 2020;46(9):1696-706.

520. Huh JW, Choi HS, Lim CM, Koh Y, Oh YM, Shim TS, et al. Low-dose hydrocortisone treatment for patients with septic shock: a pilot study comparing 3days with 7days. Respirology. 2011;16(7):1088-95.

521. Jiang X, Zhao H, Yang F, Yuan Z, Lu P, Tian H. Early low-dose glucocorticoid therapy effectively suppresses serum pro-inflammatory factors such as IL-6 and inhibits apoptosis of CD4+ cells in septic shock patients. Int J Clin Exp Med. 2019;12(1):718-23.

522. Kaufmann I, Briegel J, Schliephake F, Hoelzl A, Chouker A, Hummel T, et al. Stress doses of hydrocortisone in septic shock: beneficial effects on opsonization-dependent neutrophil functions. Intensive Care Med. 2008;34(2):344-9.

523. Keh D, Boehnke T, Weber-Cartens S, Schulz C, Ahlers O, Bercker S, et al. Immunologic and hemodynamic effects of "low-dose" hydrocortisone in septic shock: a double-blind, randomized, placebo-controlled, crossover study. Am J Respir Crit Care Med. 2003;167(4):512-20.

524. Keh D, Trips E, Marx G, Wirtz SP, Abduljawwad E, Bercker S, et al. Effect of Hydrocortisone on Development of Shock Among Patients With Severe Sepsis: The HYPRESS Randomized Clinical Trial. JAMA. 2016;316(17):1775-85.

525. Laviolle B, Annane D, Fougerou C, Bellissant E. Gluco- and mineralocorticoid biological effects of a 7-day treatment with low doses of hydrocortisone and fludrocortisone in septic shock. Intensive Care Med. 2012;38(8):1306-14.

526. Lv QQ, Gu XH, Chen QH, Yu JQ, Zheng RQ. Early initiation of low-dose hydrocortisone treatment for septic shock in adults: A randomized clinical trial. Am J Emerg Med. 2017;35(12):1810-4.

527. Moreno R, Sprung CL, Annane D, Chevret S, Briegel J, Keh D, et al. Time course of organ failure in patients with septic shock treated with hydrocortisone: results of the Corticus study. Intensive Care Med. 2011;37(11):1765-72.

528. Ngaosuwan K, Ounchokdee K, Chalermchai T. Clinical Outcomes of Minimized Hydrocortisone Dosage of 100 Mg/Day on Lower Occurrence of Hyperglycemia in Septic Shock Patients. Shock. 2018;50(3):280-5.

529. Oppert M, Schindler R, Husung C, Offermann K, Gräf KJ, Boenisch O, et al. Low-dose hydrocortisone improves shock reversal and reduces cytokine levels in early hyperdynamic septic shock. Crit Care Med. 2005;33(11):2457-64.

530. Rinaldi S, Adembri C, Grechi S, De Gaudio AR. Low-dose hydrocortisone during severe sepsis: effects on microalbuminuria. Crit Care Med. 2006;34(9):2334-9.

531. Sprung CL, Annane D, Keh D, Moreno R, Singer M, Freivogel K, et al. Hydrocortisone therapy for patients with septic shock. N Engl J Med. 2008;358(2):111-24.

532. Tilouche N, Jaoued O, Ali HBS, Gharbi R, Fekih Hassen M, Elatrous S. Comparison Between Continuous and Intermittent Administration of Hydrocortisone During Septic Shock: A Randomized Controlled Clinical Trial. Shock. 2019;52(5):481-6.

533. Tongyoo S, Permpikul C, Mongkolpun W, Vattanavanit V, Udompanturak S, Kocak M, Meduri GU. Hydrocortisone treatment in early sepsis-associated acute respiratory distress syndrome: results of a randomized controlled trial. Crit Care. 2016;20(1):329.

534. Venkatesh B, Finfer S, Cohen J, Rajbhandari D, Arabi Y, Bellomo R, et al. Adjunctive Glucocorticoid Therapy in Patients with Septic Shock. N Engl J Med. 2018;378(9):797-808.

535. Yildiz O, Doganay M, Aygen B, Güven M, Keleştimur F, Tutuû A. Physiological-dose steroid therapy in sepsis [ISRCTN36253388]. Crit Care. 2002;6(3):251-9.

536. Yildiz O, Tanriverdi F, Simsek S, Aygen B, Kelestimur F. The effects of moderate-dose steroid therapy in sepsis: A placebo-controlled, randomized study. J Res Med Sci. 2011;16(11):1410-21.

537. Ram GK, Shekhar S, Singh RB, Anand R, De RR, Kumar N. Hyperglycemia Risk Evaluation of Hydrocortisone Intermittent Boluses versus Continuous Infusion in Septic Shock: A Prospective Randomized Trial. Anesth Essays Res. 2022;16(3):321-5.

538. Birudaraju D, Hamal S, Tayek JA. Solumedrol Treatment for Severe Sepsis in Humans with a Blunted Adrenocorticotropic Hormone-Cortisol Response: A Prospective Randomized Double-Blind Placebo-Controlled Pilot Clinical Trial. J Intensive Care Med. 2022;37(5):693-7.

539. Agarwal M, Dhar M, Agarwal D, Murlidharan A. Early Initiation of Low-Dose Hydrocortisone Therapy for Septic Shock in Geriatric Patients: A Randomized Control Trial. J Assoc Physicians India. 2022;70(2):11-2.

540. Torres A, Sibila O, Ferrer M, Polverino E, Menendez R, Mensa J, et al. Effect of Corticosteroids on Treatment Failure Among Hospitalized Patients With Severe Community-Acquired Pneumonia and High Inflammatory Response: A Randomized Clinical Trial. JAMA. 2015;313(7):677-86.

541. Dequin PF, Meziani F, Quenot JP, Kamel T, Ricard JD, Badie J, et al. Hydrocortisone in Severe Community-Acquired Pneumonia. N Engl J Med. 2023;388(21):1931-41.

542. Klastersky J, Capel R. Adreno-corticosteroids in the treatment of bacterial sepsis: a double-blind study with pharmacological doses. Antimicrob Agents Chemother (Bethesda). 1970;10:175-80.

543. Schumer W. Steroids in the treatment of clinical septic shock. Ann Surg. 1976;184(3):333-41.

544. Sprung CL, Caralis PV, Marcial EH, Pierce M, Gelbard MA, Long WM, et al. The effects of high-dose corticosteroids in patients with septic shock. A prospective, controlled study. N Engl J Med. 1984;311(18):1137-43.

545. Bone RC, Fisher CJ, Jr., Clemmer TP, Slotman GJ, Metz CA, Balk RA. A controlled clinical trial of high-dose methylprednisolone in the treatment of severe sepsis and septic shock. N Engl J Med. 1987;317(11):653-8.

546. Luce JM, Montgomery AB, Marks JD, Turner J, Metz CA, Murray JF. Ineffectiveness of high-dose methylprednisolone in preventing parenchymal lung injury and improving mortality in patients with septic shock. Am Rev Respir Dis. 1988;138(1):62-8.

547. Bollaert PE, Charpentier C, Levy B, Debouverie M, Audibert G, Larcan A. Reversal of late septic shock with supraphysiologic doses of hydrocortisone. Crit Care Med. 1998;26(4):645-50.

548. Schelling G, Briegel J, Roozendaal B, Stoll C, Rothenhäusler HB, Kapfhammer HP. The effect of stress doses of hydrocortisone during septic shock on posttraumatic stress disorder in survivors. Biol Psychiatry. 2001;50(12):978-85.

549. Confalonieri M, Urbino R, Potena A, Piattella M, Parigi P, Puccio G, et al. Hydrocortisone infusion for severe community-acquired pneumonia: a preliminary randomized study. Am J Respir Crit Care Med. 2005;171(3):242-8.

550. Mussack T, Briegel J, Schelling G, Biberthaler P, Jochum M. Effect of stress doses of hydrocortisone on S-100B vs. interleukin-8 and polymorphonuclear elastase levels in human septic shock. Clin Chem Lab Med. 2005;43(3):259-68.

551. Hyvernat H, Barel R, Gentilhomme A, Césari-Giordani JF, Freche A, Kaidomar M, et al. Effects of Increasing Hydrocortisone to 300 mg Per Day in the Treatment of Septic Shock: a Pilot Study. Shock. 2016;46(5):498-505.

552. Group VASSCS. Effect of high-dose glucocorticoid therapy on mortality in patients with clinical signs of systemic sepsis. N Engl J Med. 1987;317(11):659-65.

553. Briegel J, Forst H, Haller M, Schelling G, Kilger E, Kuprat G, et al. Stress doses of hydrocortisone reverse hyperdynamic septic shock: a prospective, randomized, double-blind, single-center study. Crit Care Med. 1999;27(4):723-32.

554. Briegel J, Jochum M, Gippner-Steppert C, Thiel M. Immunomodulation in septic shock: hydrocortisone differentially regulates cytokine responses. J Am Soc Nephrol. 2001;12 Suppl 17:S70-4.

555. Cicarelli DD, Benseñor FE, Vieira JE. Effects of single dose of dexamethasone on patients with systemic inflammatory response. Sao Paulo Med J. 2006;124(2):90-5.

556. Cicarelli DD, Vieira JE, Benseñor FE. Early dexamethasone treatment for septic shock patients: a prospective randomized clinical trial. Sao Paulo Med J. 2007;125(4):237-41.

557. Wani SJ, Mufti SA, Jan RA, Shah SU, Qadri SM, Khan UH, et al. Combination of vitamin C, thiamine and hydrocortisone added to standard treatment in the management of sepsis: results from an open label randomised controlled clinical trial and a review of the literature. Infect Dis (Lond). 2020;52(4):271-8.

558. Chang P, Liao Y, Guan J, Guo Y, Zhao M, Hu J, et al. Combined Treatment With Hydrocortisone, Vitamin C, and Thiamine for Sepsis and Septic Shock: A Randomized Controlled Trial. Chest. 2020;158(1):174-82.

559. Reddy PR, Samavedam S, Aluru N, Yelle S, Rajyalakshmi B. Metabolic Resuscitation Using Hydrocortisone, Ascorbic Acid, and Thiamine: Do Individual Components Influence Reversal of Shock Independently? Indian J Crit Care Med. 2020;24(8):649-52.

560. Hussein AA, Sabry NA, Abdalla MS, Farid SF. A prospective, randomised clinical study comparing triple therapy regimen to hydrocortisone monotherapy in reducing mortality in septic shock patients. Int J Clin Pract. 2021;75(9):e14376.

561. Lyu QQ, Zheng RQ, Chen QH, Yu JQ, Shao J, Gu XH. Early administration of hydrocortisone, vitamin C, and thiamine in adult patients with septic shock: a randomized controlled clinical trial. Crit Care. 2022;26(1):295.

562. Raghu K, Ramalingam K. Safety and Efficacy of Vitamin C, Vitamin B1, and Hydrocortisone in clinical outcome of septic shock receiving standard care: A quasi experimental randomized open label two arm parallel group study. European Journal of Molecular and Clinical Medicine. 2021;8:873+.

563. Balakrishnan M, Gandhi H, Shah K, Pandya H, Patel R, Keshwani S, Yadav N. Hydrocortisone, Vitamin C and thiamine for the treatment of sepsis and septic shock following cardiac surgery. Indian J Anaesth. 2018;62(12):934-9.

564. Fujii T, Luethi N, Young PJ, Frei DR, Eastwood GM, French CJ, et al. Effect of Vitamin C, Hydrocortisone, and Thiamine vs Hydrocortisone Alone on Time Alive and Free of Vasopressor Support Among Patients With Septic Shock: The VITAMINS Randomized Clinical Trial. Jama. 2020;323(5):423-31.

565. Iglesias J, Vassallo AV, Patel VV, Sullivan JB, Cavanaugh J, Elbaga Y. Outcomes of Metabolic Resuscitation Using Ascorbic Acid, Thiamine, and Glucocorticoids in the Early Treatment of Sepsis: The ORANGES Trial. Chest. 2020;158(1):164-73.

566. Moskowitz A, Huang DT, Hou PC, Gong J, Doshi PB, Grossestreuer AV, et al. Effect of Ascorbic Acid, Corticosteroids, and Thiamine on Organ Injury in Septic Shock: The ACTS Randomized Clinical Trial. Jama. 2020;324(7):642-50.

567. Wang J, Song Q, Yang S, Wang H, Meng S, Huang L, et al. Effects of hydrocortisone combined with vitamin C and vitamin B1 versus hydrocortisone alone on microcirculation in septic shock patients: A pilot study. Clin Hemorheol Microcirc. 2023;84(2):111-23.

568. Jamshidi MR, Zeraati MR, Forouzanfar B, Tahrekhani M, Motamed N. Effects of triple combination of hydrocortisone, thiamine, and Vitamin C on clinical outcome in patients with septic shock: A single-center randomized controlled trial. J Res Med Sci. 2021;26:47.

569. Sevransky JE, Rothman RE, Hager DN, Bernard GR, Brown SM, Buchman TG, et al. Effect of Vitamin C, Thiamine, and Hydrocortisone on Ventilator- and Vasopressor-Free Days in Patients With Sepsis: The VICTAS Randomized Clinical Trial. JAMA. 2021;325(8):742-50.

570. Williams Roberson S, Nwosu S, Collar EM, Kiehl AL, Harrison FE, Bastarache J, et al. Association of Vitamin C, Thiamine, and Hydrocortisone Infusion With Long-term Cognitive, Psychological, and Functional Outcomes in Sepsis Survivors: A Secondary Analysis of the Vitamin C, Thiamine, and Steroids in Sepsis Randomized Clinical Trial. JAMA Netw Open. 2023;6(2):e230380.

571. Mohamed A, Abdelaty M, Saad MO, Shible A, Mitwally H, Akkari AR, et al. EVALUATION OF HYDROCORTISONE, VITAMIN C, AND THIAMINE FOR THE TREATMENT OF SEPTIC SHOCK: A RANDOMIZED CONTROLLED TRIAL (THE HYVITS TRIAL). Shock. 2023;59(5):697-701.

572. Truwit JD, Bernard GR, Steingrub J, Matthay MA, Liu KD, Albertson TE, et al. Rosuvastatin for sepsis-associated acute respiratory distress syndrome. N Engl J Med. 2014;370(23):2191-200.

573. El-Akabawy H, Zaghla HE, Samer N, El-Raof A, editors. Effect of Statin Therapy in Early Sepsis : Effect on Endothelial Function and Prognostic Implication2013.

574. Kruger P, Bailey M, Bellomo R, Cooper DJ, Harward M, Higgins A, et al. A multicenter randomized trial of atorvastatin therapy in intensive care patients with severe sepsis. Am J Respir Crit Care Med. 2013;187(7):743-50.

575. Kruger PS, Harward ML, Jones MA, Joyce CJ, Kostner KM, Roberts MS, Venkatesh B. Continuation of statin therapy in patients with presumed infection: a randomized controlled trial. Am J Respir Crit Care Med. 2011;183(6):774-81.

576. Patel JM, Snaith C, Thickett DR, Linhartova L, Melody T, Hawkey P, et al. Randomized double-blind placebo-controlled trial of 40 mg/day of atorvastatin in reducing the severity of sepsis in ward patients (ASEPSIS Trial). Crit Care. 2012;16(6):R231.

577. Shao H, Wang C, Zhu W, Huang X, Guo Z, Zhang H, Qin B. Different regulation of Toll-like receptor 4 expression on blood CD14(+) monocytes by simvastatin in patients with sepsis and severe sepsis. Int J Clin Exp Med. 2015;8(8):13830-5.

578. Singh RK, Agarwal V, Baronia AK, Kumar S, Poddar B, Azim A. The Effects of Atorvastatin on Inflammatory Responses and Mortality in Septic Shock: A Single-center, Randomized Controlled Trial. Indian J Crit Care Med. 2017;21(10):646-54.

579. Moskowitz A, Berg KM, Grossestreuer AV, Balaji L, Liu X, Cocchi MN, et al. Thiamine for Renal Protection in Septic Shock (TRPSS): A Randomized, Placebo-controlled, Clinical Trial. Am J Respir Crit Care Med. 2023;208(5):570-8.

580. Rosengrave P, Spencer E, Williman J, Mehrtens J, Morgan S, Doyle T, et al. Intravenous vitamin C administration to patients with septic shock: a pilot randomised controlled trial. Crit Care. 2022;26(1):26.

581. El Driny WA, Esmat IM, Shaheen SM, Sabri NA. Efficacy of High-Dose Vitamin C Infusion on Outcomes in Sepsis Requiring Mechanical Ventilation: A Double-Blind Randomized Controlled Trial. Anesthesiol Res Pract. 2022;2022:4057215.

582. Fowler AA, 3rd, Syed AA, Knowlson S, Sculthorpe R, Farthing D, DeWilde C, et al. Phase I safety trial of intravenous ascorbic acid in patients with severe sepsis. J Transl Med. 2014;12:32.

583. Fowler AA, 3rd, Truwit JD, Hite RD, Morris PE, DeWilde C, Priday A, et al. Effect of Vitamin C Infusion on Organ Failure and Biomarkers of Inflammation and Vascular Injury in Patients With Sepsis and Severe Acute Respiratory Failure: The CITRIS-ALI Randomized Clinical Trial. Jama. 2019;322(13):1261-70.

584. Yanase F, Spano S, Maeda A, Chaba A, Naorungroj T, Ow CPC, et al. Mega-dose sodium ascorbate: a pilot, single-dose, physiological effect, double-blind, randomized, controlled trial. Critical Care. 2023;27(1):371.

585. Belousoviene E, Pranskuniene Z, Vaitkaitiene E, Pilvinis V, Pranskunas A. Effect of high-dose intravenous ascorbic acid on microcirculation and endothelial glycocalyx during sepsis and septic shock: a double-blind, randomized, placebo-controlled study. BMC Anesthesiology. 2023;23(1):309.

586. Lamontagne F, Masse MH, Menard J, Sprague S, Pinto R, Heyland DK, et al. Intravenous Vitamin C in Adults with Sepsis in the Intensive Care Unit. N Engl J Med. 2022;386(25):2387-98.

587. Lubis B, Ganie RA, Lelo A, Wijaya DW, Munir D, Nasution AH. Combination Therapy of Vitamin C and Thiamine on Matrix Metalloproteinases-9 (Mmp-9) for Septic in ICU. 2021.

588. Janka V, Ladislav K, Jozef F, Ladislav V. Restoration of antioxidant enzymes in the therapeutic use of selenium in septic patients. Wien Klin Wochenschr. 2013;125(11-12):316-25.

589. Cai G, Yan J, Zhang Z, Yu Y. Immunomodulatory effects of glutamine-enriched nutritional support in elderly patients with severe sepsis: a prospective, randomized, controlled study. Journal of Organ Dysfunction. 2008;4(1):31-7.

590. Cavalcante AA, Campelo MW, de Vasconcelos MP, Ferreira CM, Guimarães SB, Garcia JH, de Vasconcelos PR. Enteral nutrition supplemented with L-glutamine in patients with systemic inflammatory response syndrome due to pulmonary infection. Nutrition. 2012;28(4):397-402.

591. Elmokadem E, Sabri N, Roshdy T, Hasanin A. Clinical outcome study of critically-ill septic patients given taurine supplemented enteral nutrition. International Journal of Pharmaceutical Sciences and Research. 2015;6(8):3544.

592. Aisa-Alvarez A, Soto ME, Guarner-Lans V, Camarena-Alejo G, Franco-Granillo J, Martínez-Rodríguez EA, et al. Usefulness of Antioxidants as Adjuvant Therapy for Septic Shock: A Randomized Clinical Trial. Medicina (Kaunas). 2020;56(11).

593. Pérez-Torres I, Aisa-Álvarez A, Casarez-Alvarado S, Borrayo G, Márquez-Velasco R, Guarner-Lans V, et al. Impact of Treatment with Antioxidants as an Adjuvant to Standard Therapy in Patients with Septic Shock: Analysis of the Correlation between Cytokine Storm and Oxidative Stress and Therapeutic Effects. Int J Mol Sci. 2023;24(23).

594. Shah FA, Kitsios GD, Yende S, Dunlap DG, Scholl D, Chuan B, et al. A Pilot Double-Blind Placebo-Controlled Randomized Clinical Trial to Investigate the Effects of Early Enteral Nutrients in Sepsis. Crit Care Explor. 2021;3(10):e550.

595. Gultekin G, Sahin H, Inanc N, Uyanik F, Ok E. Impact of Omega-3 and Omega-9 fatty acids enriched total parenteral nutrition on blood chemistry and inflammatory markers in septic patients. Pak J Med Sci. 2014;30(2):299-304.

596. Hosny M, Nahas R, Ali S, Elshafei SA, Khaled H. Impact of oral omega-3 fatty acids supplementation in early sepsis on clinical outcome and immunomodulation. The Egyptian Journal of Critical Care Medicine. 2013;1(3):119-26.

597. Mayer K, Gokorsch S, Fegbeutel C, Hattar K, Rosseau S, Walmrath D, et al. Parenteral nutrition with fish oil modulates cytokine response in patients with sepsis. Am J Respir Crit Care Med. 2003;167(10):1321-8.

598. Sungurtekin H, Değirmenci S, Sungurtekin U, Oguz BE, Sabir N, Kaptanoglu B. Comparison of the effects of different intravenous fat emulsions in patients with systemic inflammatory response syndrome and sepsis. Nutr Clin Pract. 2011;26(6):665-71.

599. Elay G, Gündoğan K, Güntürk İ, Temel Ş, Özer NT, Sipahioğlu H, et al. The Effects of Genistein as Supplement to Oral/Enteral Nutrition on Inflammatory Cytokines in Septic ICU patients: A Prospective, Single-center, Randomized Controlled Pilot Study. J Clin Pract Res. 2023;45(2):131-7.

600. Evans CR, Karnovsky A, Puskarich MA, Michailidis G, Jones AE, Stringer KA. Untargeted Metabolomics Differentiates l-Carnitine Treated Septic Shock 1-Year Survivors and Nonsurvivors. J Proteome Res. 2019;18(5):2004-11.

601. Galbán C, Montejo JC, Mesejo A, Marco P, Celaya S, Sánchez-Segura JM, et al. An immune-enhancing enteral diet reduces mortality rate and episodes of bacteremia in septic intensive care unit patients. Crit Care Med. 2000;28(3):643-8.

602. Wang H, Hu X, Wang T, Cui C, Jiang J, Dong K, et al. Exposure-Response Modeling to Support Dosing Selection for Phase IIb Development of Kukoamine B in Sepsis Patients. Front Pharmacol. 2021;12:645130.

603. Karimi A, Mahmoodpoor A, Kooshki F, Niazkar HR, Shoorei H, Tarighat-Esfanjani A. Effects of nanocurcumin on inflammatory factors and clinical outcomes in critically ill patients with sepsis: A pilot randomized clinical trial. European Journal of Integrative Medicine. 2020;36:101122.

604. Naeini F, Tutunchi H, Razmi H, Mahmoodpoor A, Vajdi M, Sefidmooye Azar P, et al. Does nano-curcumin supplementation improve hematological indices in critically ill patients with sepsis? A randomized controlled clinical trial. J Food Biochem. 2022;46(5):e14093.

605. Karimi A, Naeini F, Niazkar HR, Tutunchi H, Musazadeh V, Mahmoodpoor A, et al. Nano-curcumin supplementation in critically ill patients with sepsis: a randomized clinical trial investigating the inflammatory biomarkers, oxidative stress indices, endothelial function, clinical outcomes and nutritional status. Food Funct. 2022;13(12):6596-612.

606. Karimi A, Pourreza S, Vajdi M, Mahmoodpoor A, Sanaie S, Karimi M, Tarighat-Esfanjani A. Evaluating the effects of curcumin nanomicelles on clinical outcome and cellular immune responses in critically ill sepsis patients: A randomized, double-blind, and placebo-controlled trial. Front Nutr. 2022;9:1037861.

607. Brodska H, Valenta J, Malickova K, Kohout P, Kazda A, Drabek T. Biomarkers in critically ill patients with systemic inflammatory response syndrome or sepsis supplemented with high-dose selenium. J Trace Elem Med Biol. 2015;31:25-32.

608. Chelkeba L, Ahmadi A, Abdollahi M, Najafi A, Ghadimi MH, Mosaed R, Mojtahedzadeh M. The effect of parenteral selenium on outcomes of mechanically ventilated patients following sepsis: a prospective randomized clinical trial. Ann Intensive Care. 2015;5(1):29.

609. Chelkeba L, Ahmadi A, Abdollahi M, Najafi A, Ghadimi MH, Mosaed R, Mojtahedzadeh M. The Effect of High-dose Parenteral Sodium Selenite in Critically Ill Patients following Sepsis: A Clinical and Mechanistic Study. Indian J Crit Care Med. 2017;21(5):287-93.

610. Guo A, Srinath J, Feuerecker M, Crucian B, Briegel J, Boulesteix AL, et al. Immune function testing in sepsis patients receiving sodium selenite. J Crit Care. 2019;52:208-12.

611. Mishra V, Baines M, Perry SE, McLaughlin PJ, Carson J, Wenstone R, Shenkin A. Effect of selenium supplementation on biochemical markers and outcome in critically ill patients. Clin Nutr. 2007;26(1):41-50.

612. Valenta J, Brodska H, Drabek T, Hendl J, Kazda A. High-dose selenium substitution in sepsis: a prospective randomized clinical trial. Intensive Care Med. 2011;37(5):808-15.

613. Hazrati E, Rafii MRR, Shekarchi B, Hosseini-Shokouh SJ. The effect of selenase on the disease process of patients with septic shock admitted to the intensive care unit: A clinical trial in Tehran. Annals of Military and Health Sciences Research. 2015;13(1).

614. Cherukuri L, Gewirtz G, Osea K, Tayek JA. Vitamin A treatment for severe sepsis in humans; a prospective randomized double blind placebo-controlled clinical trial. Clin Nutr ESPEN. 2019;29:49-51.

615. Leaf DE, Raed A, Donnino MW, Ginde AA, Waikar SS. Randomized controlled trial of calcitriol in severe sepsis. Am J Respir Crit Care Med. 2014;190(5):533-41.

616. Quraishi SA, De Pascale G, Needleman JS, Nakazawa H, Kaneki M, Bajwa EK, et al. Effect of Cholecalciferol Supplementation on Vitamin D Status and Cathelicidin Levels in Sepsis: A Randomized, Placebo-Controlled Trial. Crit Care Med. 2015;43(9):1928-37.

617. Jokar A, Ahmadi K, Taherinia A, Didgar F, Kazemi F, Bahramian M. The Effects of Injected Vitamin D on Prognosis of Patients with Urosepsis. Horm Metab Res. 2018;50(5):383-8.

618. Memiş D, Eskiocak S, Bayrak F, Batmaz E. The influence of alpha-tocopherol on cytokine levels and gastric intramucosal ph in severe sepsis. Trakya Üniversitesi Tıp Fakültesi Dergisi. 2008.

619. Bonifazi M, Meessen J, Pérez A, Vasques F, Busana M, Vassalli F, et al. Albumin Oxidation Status in Sepsis Patients Treated With Albumin or Crystalloids. Front Physiol. 2021;12:682877.

620. Pandharipande PP, Sanders RD, Girard TD, McGrane S, Thompson JL, Shintani AK, et al. Effect of dexmedetomidine versus lorazepam on outcome in patients with sepsis: an a priori-designed analysis of the MENDS randomized controlled trial. Crit Care. 2010;14(2):R38.

621. Castro R, Regueira T, Aguirre ML, Llanos OP, Bruhn A, Bugedo G, et al. An evidence-based resuscitation algorithm applied from the emergency room to the ICU improves survival of severe septic shock. Minerva Anestesiol. 2008;74(6):223-31.

622. Moeen SM, Abdelhakeem EE, Abdalla EM, Embaby I, Hassan HM, Ismail EA. Dexmedetomidine versus propofol or midazolam in patients with abdominal sepsis regarding inflammatory response and capillary leak. Egyptian Journal of Anaesthesia. 2022;38(1):23-32.

623. Chen L, Lan Y, Zhu J-C, He Y. Effects of dexmedetomidine on renal function in patients with septic shock. European Journal of Inflammation. 2018;16:2058739218794062.

624. Liu J, Shi K, Hong J, Gong F, Mo S, Chen M, et al. Dexmedetomidine protects against acute kidney injury in patients with septic shock. Ann Palliat Med. 2020;9(2):224-30.

625. Elayashy M, Elsayed EA, Mukhtar AM, Kasem S, Elmetwally SA, Habib S, et al. Role of dexmedetomidine in modifying immune paralysis in patients with septic shock: randomized controlled trial. Intensive Care Med Exp. 2023;11(1):59.

626. Tasdogan M, Memis D, Sut N, Yuksel M. Results of a pilot study on the effects of propofol and dexmedetomidine on inflammatory responses and intraabdominal pressure in severe sepsis. J Clin Anesth. 2009;21(6):394-400.

627. Zhou WJ, Cui JK, Liu M, Shang XK, Ding SS. Comparison of norepinephrine, dopamine and dobutamine combined with enteral nutrition in the treatment of elderly patients harboring sepsis. Pak J Pharm Sci. 2021;34(3):957-61.

628. Cocchi MN, Dargin J, Chase M, Patel PV, Grossestreuer A, Balaji L, et al. Esmolol to Treat the Hemodynamic Effects of Septic Shock: A Randomized Controlled Trial. Shock. 2022;57(4):508-17.

629. Savioli M, Cugno M, Polli F, Taccone P, Bellani G, Spanu P, et al. Tight glycemic control may favor fibrinolysis in patients with sepsis. Crit Care Med. 2009;37(2):424-31.

630. Sun T, Zhang N, Cui N, Wang SH, Ding XX, Li N, et al. Efficacy of Levosimendan in the Treatment of Patients With Severe Septic Cardiomyopathy. J Cardiothorac Vasc Anesth. 2023;37(3):344-9.

631. Yin T, Liu Y, Zhao C, Guo S. Effects of levosimendan combined with routine therapy on markers for cardiac function, inflammatory factors, and apache II score in patients with septic shock. Acta Medica Mediterranea. 2021;37(4):2359-64.

632. Jiang D, Liu A, Ling W. Effects of levosimendan on serum endothelial cell specific molecule 1, procalcitonin and high-mobility group protein B1 in patients with sepsis. Acta medica mediterranea. 2020;36(3):1381-5.

633. Berger C, Rossaint J, Van Aken H, Westphal M, Hahnenkamp K, Zarbock A. Lidocaine reduces neutrophil recruitment by abolishing chemokine-induced arrest and transendothelial migration in septic patients. J Immunol. 2014;192(1):367-76.

634. Chen L, Meng K, Su W, Fu Y. The Effect of Continuous Sedation Therapy on Immunomodulation, Plasma Levels of Antioxidants, and Indicators of Tissue Repair in Post-Burn Sepsis Patients. Cell Biochem Biophys. 2015;73(2):473-8.

635. Sahoo P, Kothari N, Goyal S, Sharma A, Bhatia PK. Comparison of Norepinephrine and Terlipressin vs Norepinephrine Alone for Management of Septic Shock: A Randomized Control Study. Indian J Crit Care Med. 2022;26(6):669-75.

636. Anthon CT, Müller RB, Haase N, Hjortrup PB, Møller K, Lange T, et al. Effects of hydroxyethyl starch 130/0.42 vs. Ringer's acetate on cytokine levels in severe sepsis. Acta Anaesthesiol Scand. 2017;61(8):904-13.

637. Gao Y, Zhu J, Yin C, Zhu J, Zhu T, Liu L. Effects of Target Temperature Management on the Outcome of Septic Patients with Fever. BioMed Research International. 2017;2017:3906032.

638. Saoraya J, Musikatavorn K, Puttaphaisan P, Komindr A, Srisawat N. Intensive fever control using a therapeutic normothermia protocol in patients with febrile early septic shock: A randomized feasibility trial and exploration of the immunomodulatory effects. SAGE Open Med. 2020;8:2050312120928732.

639. Drewry AM, Mohr NM, Ablordeppey EA, Dalton CM, Doctor RJ, Fuller BM, et al. Therapeutic Hyperthermia Is Associated With Improved Survival in Afebrile Critically Ill Patients With Sepsis: A Pilot Randomized Trial. Crit Care Med. 2022;50(6):924-34.

640. Yuxi Q, Zhang H, Baili Y, Shi S. Effects of Xuebijing Injection for Patients With Sepsis-induced Acute Kidney Injury After Wenchuan Earthquake. Altern Ther Health Med. 2017;23(2):36-42.

641. Yang G, Zheng B, Yu Y, Huang J, Zhu H, Deng D, Li J. Electroacupuncture at Zusanli (ST36), Guanyuan (CV4), and Qihai (CV6) Acupoints Regulates Immune Function in Patients with Sepsis via the PD-1 Pathway. Biomed Res Int. 2022;2022:7037497.

642. Meng JB, Jiao YN, Xu XJ, Lai ZZ, Zhang G, Ji CL, Hu MH. Electro-acupuncture attenuates inflammatory responses and intraabdominal pressure in septic patients: A randomized controlled trial. Medicine (Baltimore). 2018;97(17):e0555.

643. Eslami K, Mahmoodpoor A, Ahmadi A, Abdollahi M, Kamali K, Mousavi S, et al. Positive effect of septimeb™ on mortality rate in severe sepsis: a novel non antibiotic strategy. Daru. 2012;20(1):40.

644. Wu X, He C, Liu C, Xu X, Chen C, Yang H, et al. Mechanisms of JinHong Formula on treating sepsis explored by randomized controlled trial combined with network pharmacology. J Ethnopharmacol. 2023;305:116040.

645. Wang S, Liu G, Chen L, Xu X, Jia T, Zhu C, Xiong J. Effects of Shenfu injection on sublingual microcirculation in septic shock patients: a randomized controlled trial. Shock. 2022;58(3):196-203.

646. Tie M, Chen B, Lv R, Xie Q, Pang Y, Gong R. Effects of Shenling Chengqi Decoction on Gastrointestinal Function and Immune Status of Patients with Gastrointestinal Injury in Severe Sepsis. Comput Math Methods Med. 2022;2022:2219451.

647. Liu Y, Yu Y, Dai H, Fei A. Efficacy of Shufeng Jiedu Capsules in the treatment of sepsis: A prospective, randomized, placebo-controlled trial. European Journal of Integrative Medicine. 2020;38:101172.

648. Zhen G, Shan Y, Qiao H, Wang W, Gai L, Li Z. Efficacy of Xuebijing injection in the adjunctive therapy of acute respiratory distress syndrome caused by sepsis. Int J Clin Exp Med. 2019;12(8):10029-38.

649. Yin Q, Li C. Treatment effects of xuebijing injection in severe septic patients with disseminated intravascular coagulation. Evid Based Complement Alternat Med. 2014;2014:949254.

650. He J, Zhao X, Lin X, Yang Z, Ma M, Ma L, et al. The Effect of Xinmailong Infusion on Sepsis-Induced Myocardial Dysfunction: a Pragmatic Randomized Controlled Trial. Shock. 2021;55(1):33-40.

651. Cheng AC, Stephens DP, Anstey NM, Currie BJ. Adjunctive granulocyte colony-stimulating factor for treatment of septic shock due to melioidosis. Clin Infect Dis. 2004;38(1):32-7.

652. Stephens DP, Fisher DA, Currie BJ. An audit of the use of granulocyte colony-stimulating factor in septic shock. Intern Med J. 2002;32(4):143-8.

653. Endo S, Inada K, Inoue Y, Yamada Y, Takakuwa T, Kasai T, et al. Evaluation of recombinant human granulocyte colony-stimulating factor (rhG-CSF) therapy in granulopoetic patients complicated with sepsis. Curr Med Res Opin. 1994;13(4):233-41.

654. Gross-Weege W, Weiss M, Schneider M, Wenning M, Harms B, Dumon K, et al. Safety of a low-dosage Filgrastim (rhG-CSF) treatment in non-neutropenic surgical intensive care patients with an inflammatory process. Intensive Care Med. 1997;23(1):16-22.

655. Ishikawa K, Tanaka H, Nakamori Y, Hosotsubo H, Ogura H, Nishino M, et al. Difference in the responses after administration of granulocyte colony-stimulating factor in septic patients with relative neutropenia. J Trauma. 2000;48(5):814-24; discussion 24-5.

656. Nierhaus A, Montag B, Timmler N, Frings DP, Gutensohn K, Jung R, et al. Reversal of immunoparalysis by recombinant human granulocyte-macrophage colony-stimulating factor in patients with severe sepsis. Intensive Care Med. 2003;29(4):646-51.

657. Graw RG, Jr., Herzig G, Perry S, Henderson ES. Normal granulocyte transfusion therapy: treatment of septicemia due to gram-negative bacteria. N Engl J Med. 1972;287(8):367-71.

658. Kox WJ, Bone RC, Krausch D, Döcke WD, Kox SN, Wauer H, et al. Interferon gamma-1b in the treatment of compensatory anti-inflammatory response syndrome. A new approach: proof of principle. Arch Intern Med. 1997;157(4):389-93.

659. Peters C, Minkov M, Matthes-Martin S, Pötschger U, Witt V, Mann G, et al. Leucocyte transfusions from rhG-CSF or prednisolone stimulated donors for treatment of severe infections in immunocompromised neutropenic patients. Br J Haematol. 1999;106(3):689-96.

660. Cheng AC, Limmathurotsakul D, Chierakul W, Getchalarat N, Wuthiekanun V, Stephens DP, et al. A randomized controlled trial of granulocyte colony-stimulating factor for the treatment of severe sepsis due to melioidosis in Thailand. Clin Infect Dis. 2007;45(3):308-14.

661. Hartmann P, Lammertink J, Mansmann G, Hübel K, Salzberger B, Stützer H, et al. A randomized, placebo-controlled study of the use of filgrastim in non neutropenic patients with nosocomial pneumonia. Eur J Med Res. 2005;10(1):29-35.

662. Root RK, Lodato RF, Patrick W, Cade JF, Fotheringham N, Milwee S, et al. Multicenter, double-blind, placebo-controlled study of the use of filgrastim in patients hospitalized with pneumonia and severe sepsis. Crit Care Med. 2003;31(2):367-73.

663. Stephens DP, Thomas JH, Higgins A, Bailey M, Anstey NM, Currie BJ, Cheng AC. Randomized, double-blind, placebo-controlled trial of granulocyte colony-stimulating factor in patients with septic shock. Crit Care Med. 2008;36(2):448-54.

664. Tanaka H, Nishino M, Nakamori Y, Ogura H, Ishikawa K, Shimazu T, Sugimoto H. Granulocyte colony-stimulating factor (G-CSF) stiffens leukocytes but attenuates inflammatory response without lung injury in septic patients. J Trauma. 2001;51(6):1110-6.

665. Wunderink R, Leeper K, Jr., Schein R, Nelson S, DeBoisblanc B, Fotheringham N, Logan E. Filgrastim in patients with pneumonia and severe sepsis or septic shock. Chest. 2001;119(2):523-9.

666. Meisel C, Schefold JC, Pschowski R, Baumann T, Hetzger K, Gregor J, et al. Granulocyte-macrophage colony-stimulating factor to reverse sepsis-associated immunosuppression: a double-blind, randomized, placebo-controlled multicenter trial. Am J Respir Crit Care Med. 2009;180(7):640-8.

667. Orozco H, Arch J, Medina-Franco H, Pantoja JP, González QH, Vilatoba M, et al. Molgramostim (GM-CSF) associated with antibiotic treatment in nontraumatic abdominal sepsis: a randomized, double-blind, placebo-controlled clinical trial. Arch Surg. 2006;141(2):150-3; discussion 4.

668. Presneill JJ, Harris T, Stewart AG, Cade JF, Wilson JW. A randomized phase II trial of granulocyte-macrophage colony-stimulating factor therapy in severe sepsis with respiratory dysfunction. Am J Respir Crit Care Med. 2002;166(2):138-43.

669. Schefold JC, Zeden JP, Pschowski R, Hammoud B, Fotopoulou C, Hasper D, et al. Treatment with granulocyte-macrophage colony-stimulating factor is associated with reduced indoleamine 2,3-dioxygenase activity and kynurenine pathway catabolites in patients with severe sepsis and septic shock. Scand J Infect Dis. 2010;42(3):164-71.

670. Vacheron CH, Lepape A, Venet F, Monneret G, Gueyffier F, Boutitie F, et al. Granulocyte-macrophage colony-stimulating factor (GM-CSF) in patients presenting sepsis-induced immunosuppression: The GRID randomized controlled trial. J Crit Care. 2023;78:154330.

671. Prakash V, Arora V, Jindal A, Maiwall R, Sarin SK. Combination of GM CSF and carbapenem is superior to carbapenem monotherapy in difficult-to-treat spontaneous bacterial peritonitis: A randomized controlled trial. Liver Int. 2023;43(6):1298-306.

672. Herzig RH, Herzig GP, Graw RG, Jr., Bull MI, Ray KK. Successful granulocyte transfusion therapy for gram-negative septicemia. A prospectively randomized controlled study. N Engl J Med. 1977;296(13):701-5.

673. Francois B, Jeannet R, Daix T, Walton AH, Shotwell MS, Unsinger J, et al. Interleukin-7 restores lymphocytes in septic shock: the IRIS-7 randomized clinical trial. JCI Insight. 2018;3(5).

674. Daix T, Mathonnet A, Brakenridge S, Dequin PF, Mira JP, Berbille F, et al. Intravenously administered interleukin-7 to reverse lymphopenia in patients with septic shock: a double-blind, randomized, placebo-controlled trial. Ann Intensive Care. 2023;13(1):17.

675. Wu J, Zhou L, Liu J, Ma G, Kou Q, He Z, et al. The efficacy of thymosin alpha 1 for severe sepsis (ETASS): a multicenter, single-blind, randomized and controlled trial. Crit Care. 2013;17(1):R8.

676. Ishikura H, Nakamura Y, Kawano Y, Tanaka J, Mizunuma M, Ohta D, et al. Intravenous immunoglobulin improves sepsis-induced coagulopathy: A retrospective, single-center observational study. J Crit Care. 2015;30(3):579-83.

677. Tagami T, Matsui H, Fushimi K, Yasunaga H. Intravenous immunoglobulin use in septic shock patients after emergency laparotomy. J Infect. 2015;71(2):158-66.

678. Akatsuka M, Masuda Y, Tatsumi H, Sonoda T. Efficacy of Intravenous Immunoglobulin Therapy for Patients With Sepsis and Low Immunoglobulin G Levels: A Single-Center Retrospective Study. Clin Ther. 2022;44(2):295-303.

679. Goto K, Yasuda N, Sato Y. Effects of low-dose intravenous immunoglobulin as the adjunctive therapy in septic shock patients with and without hypogammaglobulinemia: a retrospective cohort study. Ann Palliat Med. 2022;11(8):2600-8.

680. Senda A, Endo A, Fushimi K, Otomo Y. Effectiveness of intravenous immunoglobulin therapy for invasive group A Streptococcus infection: A Japanese nationwide observational study. Int J Infect Dis. 2023;135:84-90.

681. Iizuka Y, Sanui M, Sasabuchi Y, Lefor AK, Hayakawa M, Saito S, et al. Low-dose immunoglobulin G is not associated with mortality in patients with sepsis and septic shock. Crit Care. 2017;21(1):181.

682. Pilz G, Kääb S, Neeser G, Class I, Schweigart U, Brähler A, et al. Supplemental immunoglobulin (ivIgG) treatment in 163 patients with sepsis and septic shock--an observational study as a prerequisite for placebo-controlled clinical trials. Infection. 1991;19(4):216-27.

683. Takahashi G, Shibata S. Study of usefulness of low-dose IgG for patients with septic disseminated intravascular coagulation. Biomark Med. 2020;14(13):1189-96.

684. Hedetoft M, Madsen MB, Perner A, Garred P, Hyldegaard O. Effect of immunoglobulin G on cytokine response in necrotising soft-tissue infection: A post hoc analysis. Acta Anaesthesiol Scand. 2021;65(9):1293-9.

685. Yavuz L, Aynali G, Aynali A, Alaca A, Kutuk S, Ceylan BG. The effects of adjuvant immunoglobulin M-enriched immunoglobulin therapy on mortality rate and renal function in sepsis-induced multiple organ dysfunction syndrome: retrospective analysis of intensive care unit patients. J Int Med Res. 2012;40(3):1166-74.

686. Paternoster G, Rosa SD, Bertini P, Innelli P, Vignale R, Tripodi VF, et al. Comparative Effectiveness of Combined IgM-Enriched Immunoglobulin and Extracorporeal Blood Purification Plus Standard Care Versus Standard Care for Sepsis and Septic Shock after Cardiac Surgery. RCM. 2022;23(9).

687. Scarpati G, Baldassarre D, Tripepi G, Boffardi M, Piazza O. Effect of Intravenous IgM-Enriched Immunoglobulins on Presepsin and Other Sepsis Biomarkers. Front Pharmacol. 2021;12:717349.

688. Martinez JI, Sánchez HF, Velandia JA, Urbina Z, Florián MC, Martínez MA, et al. Treatment with IgM-enriched immunoglobulin in sepsis: a matched case-control analysis. J Crit Care. 2021;64:120-4.

689. Buda S, Riefolo A, Biscione R, Goretti E, Cattabriga I, Grillone G, et al. Clinical experience with polyclonal IgM-enriched immunoglobulins in a group of patients affected by sepsis after cardiac surgery. J Cardiothorac Vasc Anesth. 2005;19(4):440-5.

690. Giamarellos-Bourboulis EJ, Tziolos N, Routsi C, Katsenos C, Tsangaris I, Pneumatikos I, et al. Improving outcomes of severe infections by multidrug-resistant pathogens with polyclonal IgM-enriched immunoglobulins. Clin Microbiol Infect. 2016;22(6):499-506.

691. Berlot G, Dimastromatteo G. Use of IgM and IgA-enriched immunoglobulins in the treatment of severe sepsis and septic shock. Clinical experience. Minerva Anestesiol. 2004;70(10):739-43; 43-5.

692. Berlot G, Scamperle A, Istrati T, Dattola R, Longo I, Chillemi A, et al. Kinetics of Immunoglobulins in Septic Shock Patients Treated With an IgM- and IgA-Enriched Intravenous Preparation: An Observational Study. Front Med (Lausanne). 2021;8:605113.

693. Ando Y, Inoue S, Kawashima T, Okashiro M, Kotani J, Nishiyama T. Intravenous Immunoglobulin G Modulates the Expression of Sepsis-Induced Coagulopathy Factors and Increases Serum IgM Levels: A Prospective, Single-Center Intervention Study. Kobe J Med Sci. 2020;66(1):E32-e9.

694. Schellekens J, Brouwer E, Calandra T, Rozenberg-Arska M, Verhoef J. Humoral immunity in human Gram-negative septic shock II. Specific anti-bacterial immunity: opsonic activity and the effect of intravenous immunoglobulin G administration. Serodiagnosis and Immunotherapy in Infectious Disease. 1989;3(4):279-91.

695. Behre G, Schedel I, Nentwig B, Wörmann B, Essink M, Hiddemann W. Endotoxin concentration in neutropenic patients with suspected gram-negative sepsis: correlation with clinical outcome and determination of anti-endotoxin core antibodies during therapy with polyclonal immunoglobulin M-enriched immunoglobulins. Antimicrob Agents Chemother. 1992;36(10):2139-46.

696. Wand S, Klages M, Kirbach C, Warszawska J, Meybohm P, Zacharowski K, Koch A. IgM-Enriched Immunoglobulin Attenuates Systemic Endotoxin Activity in Early Severe Sepsis: A Before-After Cohort Study. PLoS One. 2016;11(8):e0160907.

697. Lindquist L, Lundbergh P, Maasing R. Pepsin-treated human gamma globulin in bacterial infections. A randomized study in patients with septicaemia and pneumonia. Vox Sang. 1981;40(5):329-37.

698. Burns ER, Lee V, Rubinstein A. Treatment of septic thrombocytopenia with immune globulin. J Clin Immunol. 1991;11(6):363-8.

699. Grundmann R, Hornung M. Immunoglobulin therapy in patients with endotoxemia and postoperative sepsis--a prospective randomized study. Prog Clin Biol Res. 1988;272:339-49.

700. Hamano N, Nishi K, Onose A, Okamoto A, Umegaki T, Yamazaki E, et al. Efficacy of single-dose intravenous immunoglobulin administration for severe sepsis and septic shock. J Intensive Care. 2013;1(1):4.

701. Schedel I, Dreikhausen U, Nentwig B, Höckenschnieder M, Rauthmann D, Balikcioglu S, et al. Treatment of gram-negative septic shock with an immunoglobulin preparation: a prospective, randomized clinical trial. Crit Care Med. 1991;19(9):1104-13.

702. Darenberg J, Ihendyane N, Sjölin J, Aufwerber E, Haidl S, Follin P, et al. Intravenous immunoglobulin G therapy in streptococcal toxic shock syndrome: a European randomized, double-blind, placebo-controlled trial. Clin Infect Dis. 2003;37(3):333-40.

703. De Simone C, Delogu G, Corbetta G. Intravenous immunoglobulins in association with antibiotics: a therapeutic trial in septic intensive care unit patients. Crit Care Med. 1988;16(1):23-6.

704. Dominioni L, Bianchi V, Imperatori A, Minoia G, Dionigi R. High-Dose Intravenous IgG for Treatment of Severe Surgical Infections. Digestive Surgery. 2008;13(4-5):430-4.

705. Dominioni L, Dionigi R, Zanello M, Chiaranda M, Dionigi R, Acquarolo A, et al. Effects of high-dose IgG on survival of surgical patients with sepsis scores of 20 or greater. Arch Surg. 1991;126(2):236-40.

706. Werdan K, Pilz G, Bujdoso O, Fraunberger P, Neeser G, Schmieder RE, et al. Score-based immunoglobulin G therapy of patients with sepsis: the SBITS study. Crit Care Med. 2007;35(12):2693-701.

707. Calandra T, Glauser MP, Schellekens J, Verhoef J. Treatment of gram-negative septic shock with human IgG antibody to Escherichia coli J5: a prospective, double-blind, randomized trial. J Infect Dis. 1988;158(2):312-9.

708. Albertson TE, Panacek EA, MacArthur RD, Johnson SB, Benjamin E, Matuschak GM, et al. Multicenter evaluation of a human monoclonal antibody to Enterobacteriaceae common antigen in patients with Gram-negative sepsis. Crit Care Med. 2003;31(2):419-27.

709. Greenman RL, Schein RM, Martin MA, Wenzel RP, MacIntyre NR, Emmanuel G, et al. A controlled clinical trial of E5 murine monoclonal IgM antibody to endotoxin in the treatment of gram-negative sepsis. The XOMA Sepsis Study Group. Jama. 1991;266(8):1097-102.

710. Hentrich M, Fehnle K, Ostermann H, Kienast J, Cornely O, Salat C, et al. IgMA-enriched immunoglobulin in neutropenic patients with sepsis syndrome and septic shock: a randomized, controlled, multiple-center trial. Crit Care Med. 2006;34(5):1319-25.

711. Toth I, Mikor A, Leiner T, Molnar Z, Bogar L, Szakmany T. Effects of IgM-enriched immunoglobulin therapy in septic-shock-induced multiple organ failure: pilot study. J Anesth. 2013;27(4):618-22.

712. Tugrul S, Ozcan PE, Akinci O, Seyhun Y, Cagatay A, Cakar N, Esen F. The effects of IgM-enriched immunoglobulin preparations in patients with severe sepsis [ISRCTN28863830]. Crit Care. 2002;6(4):357-62.

713. Brunner R, Rinner W, Haberler C, Kitzberger R, Sycha T, Herkner H, et al. Early treatment with IgM-enriched intravenous immunoglobulin does not mitigate critical illness polyneuropathy and/or myopathy in patients with multiple organ failure and SIRS/sepsis: a prospective, randomized, placebo-controlled, double-blinded trial. Crit Care. 2013;17(5):R213.

714. Domizi R, Adrario E, Damiani E, Scorcella C, Carsetti A, Giaccaglia P, et al. IgM-enriched immunoglobulins (Pentaglobin) may improve the microcirculation in sepsis: a pilot randomized trial. Ann Intensive Care. 2019;9(1):135.

715. Rodríguez A, Rello J, Neira J, Maskin B, Ceraso D, Vasta L, Palizas F. Effects of high-dose of intravenous immunoglobulin and antibiotics on survival for severe sepsis undergoing surgery. Shock. 2005;23(4):298-304.

716. Welte T, Dellinger RP, Ebelt H, Ferrer M, Opal SM, Singer M, et al. Efficacy and safety of trimodulin, a novel polyclonal antibody preparation, in patients with severe community-acquired pneumonia: a randomized, placebo-controlled, double-blind, multicenter, phase II trial (CIGMA study). Intensive Care Med. 2018;44(4):438-48.

717. Banoei MM, McIntyre LA, Stewart DJ, Mei SHJ, Courtman D, Watpool I, et al. Metabolomics Analysis of Mesenchymal Stem Cell (MSC) Therapy in a Phase I Clinical Trial of Septic Shock: An Exploratory Study. Metabolites. 2023;13(11).

718. He X, Ai S, Guo W, Yang Y, Wang Z, Jiang D, Xu X. Umbilical cord-derived mesenchymal stem (stromal) cells for treatment of severe sepsis: aphase 1 clinical trial. Transl Res. 2018;199:52-61.

719. McIntyre LA, Stewart DJ, Mei SHJ, Courtman D, Watpool I, Granton J, et al. Cellular Immunotherapy for Septic Shock. A Phase I Clinical Trial. Am J Respir Crit Care Med. 2018;197(3):337-47.

720. Schlosser K, Wang JP, Dos Santos C, Walley KR, Marshall J, Fergusson DA, et al. Effects of Mesenchymal Stem Cell Treatment on Systemic Cytokine Levels in a Phase 1 Dose Escalation Safety Trial of Septic Shock Patients. Crit Care Med. 2019;47(7):918-25.

721. Alp E, Gonen ZB, Gundogan K, Esmaoglu A, Kaynar L, Cetin A, et al. The Effect of Mesenchymal Stromal Cells on the Mortality of Patients with Sepsis and Septic Shock: A Promising Therapy. Emerg Med Int. 2022;2022:9222379.

722. Galstyan G, Makarova P, Parovichnikova E, Kuzmina L, Troitskaya V, Ghemdzhian E. The results of the single center pilot randomized Russian clinical trial of mesenchymal stromal cells in severe neutropenic patients with septic shock (RUMCESS). Int J Blood Res Disord. 2018;5(1):33.

723. Laterre PF, Sánchez García M, van der Poll T, Wittebole X, Martínez-Sagasti F, Hernandez G, et al. The safety and efficacy of stem cells for the treatment of severe community-acquired bacterial pneumonia: A randomized clinical trial. J Crit Care. 2024;79:154446.

724. Watanabe E, Nishida O, Kakihana Y, Odani M, Okamura T, Harada T, Oda S. Pharmacokinetics, Pharmacodynamics, and Safety of Nivolumab in Patients With Sepsis-Induced Immunosuppression: A Multicenter, Open-Label Phase 1/2 Study. Shock. 2020;53(6):686-94.

725. Hotchkiss RS, Colston E, Yende S, Angus DC, Moldawer LL, Crouser ED, et al. Immune Checkpoint Inhibition in Sepsis: A Phase 1b Randomized, Placebo-Controlled, Single Ascending Dose Study of Antiprogrammed Cell Death-Ligand 1 Antibody (BMS-936559). Crit Care Med. 2019;47(5):632-42.

726. Hotchkiss RS, Colston E, Yende S, Crouser ED, Martin GS, Albertson T, et al. Immune checkpoint inhibition in sepsis: a Phase 1b randomized study to evaluate the safety, tolerability, pharmacokinetics, and pharmacodynamics of nivolumab. Intensive Care Med. 2019;45(10):1360-71.

727. Carapetis JR, Jacoby P, Carville K, Ang SJ, Curtis N, Andrews R. Effectiveness of clindamycin and intravenous immunoglobulin, and risk of disease in contacts, in invasive group a streptococcal infections. Clin Infect Dis. 2014;59(3):358-65.

728. Fernández-Galilea A, Estella A, García-Garmendia JL, Loza A, Palacios-García I, Sierra-Camerino R, et al. Clindamycin but not Intravenous Immunoglobulins reduces mortality in a retrospective cohort of critically ill patients with bacteremic Group A Streptococcal infections. Rev Esp Quimioter. 2022;35(5):475-81.

729. Bissell BD, Erdman MJ, Smotherman C, Kraemer DF, Ferreira JA. The impact of endocrine supplementation on adverse events in septic shock. J Crit Care. 2015;30(6):1169-73.

730. Russell JA, Walley KR, Gordon AC, Cooper DJ, Hébert PC, Singer J, et al. Interaction of vasopressin infusion, corticosteroid treatment, and mortality of septic shock. Crit Care Med. 2009;37(3):811-8.

731. Torgersen C, Luckner G, Schröder DC, Schmittinger CA, Rex C, Ulmer H, Dünser MW. Concomitant arginine-vasopressin and hydrocortisone therapy in severe septic shock: association with mortality. Intensive Care Med. 2011;37(9):1432-7.

732. Cleasby C, Marshall T, Gordon AC, Antcliffe DB. The effect of vasopressin and hydrocortisone on cytokine trajectories. Intensive Care Med. 2023;49(2):241-3.

733. Kulesza S, Gignac L, Colvin CA, Boll S, Giuliano C, Haan B, et al. Hydrocortisone versus vasopressin for the management of adult patients with septic shock refractory to norepinephrine: A multicenter retrospective study. Pharmacotherapy. 2023;43(8):787-94.

734. Alshehri AM, Kovacevic MP, Dube KM, Lupi KE, DeGrado JR. Comparison of Early Versus Late Adjunctive Vasopressin and Corticosteroids in Patients With Septic Shock. Ann Pharmacother. 2023:10600280231191131.

735. Levy H, Laterre P-F, Bates B, Qualy RL. Steroid use in PROWESS severe sepsis patients treated with drotrecogin alfa (activated). Critical Care. 2005;9(5):R502.

736. Annane D, Timsit JF, Megarbane B, Martin C, Misset B, Mourvillier B, et al. Recombinant human activated protein C for adults with septic shock: a randomized controlled trial. Am J Respir Crit Care Med. 2013;187(10):1091-7.

737. Lei L, Wang MJ, Zhang S, Hu DJ. Effects of prostaglandin E combined with continuous renal replacement therapy on septic acute kidney injury. World J Clin Cases. 2020;8(13):2738-48.

738. Ferrer R, Artigas A, Suarez D, Palencia E, Levy MM, Arenzana A, et al. Effectiveness of treatments for severe sepsis: a prospective, multicenter, observational study. Am J Respir Crit Care Med. 2009;180(9):861-6.

739. Zhao J, Wei Q, Guo S, Wang H, Zhao C, Hu C, et al. Efficacy of Oxymatrine Plus Antiviral in the Treatment of Sepsis and Its Effect on the Levels of Endotoxin and Inflammatory Factors. Evid Based Complement Alternat Med. 2022;2022:1938325.

740. Nakamura T, Sato E, Fujiwara N, Kawagoe Y, Maeda S, Inoue H, Yamagishi S-i. Statins and hemoperfusion improve 28-day survival in septic shock patients. Open Medicine. 2012;7(4):475-80.

741. Mochizuki K, Mori K, Kamijo H, Ichikawa M, Nitta K, Imamura H. Beneficial effect modification on survival outcome of sepsis between ART-123 and polymyxin B‑immobilised haemoperfusion: a nationwide Japanese registry study. Ann Intensive Care. 2020;10(1):57.

742. Yamato M, Minematsu Y, Fujii J, Mori K, Minato T, Miyagawa S, et al. Effective combination therapy of polymyxin-B direct hemoperfusion and recombinant thrombomodulin for septic shock accompanied by disseminated intravascular coagulation: a historical controlled trial. Ther Apher Dial. 2013;17(5):472-6.

743. Li Y, Wang S, Zhang J, Cao J. Effect of a combination of Xuebijing and thymosin α1 on patients with severe pneumonia complicated with sepsis, and its influence on serum inflammatory factors. Tropical Journal of Pharmaceutical Research. 2022;21(7):1507-13.

744. Schattner A, el-Hador I, Hahn T, Landau Z. Triple anti-TNF-alpha therapy in early sepsis: a preliminary report. J Int Med Res. 1997;25(2):112-6.

745. Gordon AC, Mason AJ, Perkins GD, Stotz M, Terblanche M, Ashby D, Brett SJ. The interaction of vasopressin and corticosteroids in septic shock: a pilot randomized controlled trial. Crit Care Med. 2014;42(6):1325-33.

746. Gordon AC, Mason AJ, Thirunavukkarasu N, Perkins GD, Cecconi M, Cepkova M, et al. Effect of Early Vasopressin vs Norepinephrine on Kidney Failure in Patients With Septic Shock: The VANISH Randomized Clinical Trial. Jama. 2016;316(5):509-18.

747. Yadav A, Soni A, Tyagi N, Shekhawat K. The Interaction of Vasopressin and Corticosteroids in Septic Shock: A Study in Southern Rajasthan. International Journal of Pharmaceutical and Clinical Research. 2022(14(12)):220-6.

748. Meng F, Du C, Zhang Y, Wang S, Zhou Q, Wu L, et al. Protective effect of rhubarb combined with ulinastatin for patients with sepsis. Medicine (Baltimore). 2020;99(7):e18895.

749. Bai L, Qiu X, Ding X, Bai X, Huang W, Yang L, Shi X. Value of Thymosin α1 Combined With Blood Purification to Increase Successful Rescues of Shock Patients. Altern Ther Health Med. 2022;28(7):146-52.

750. He Y, Chen X, Zhang G, Guan L, Yu X. Clinical efficacy and safety of norepinephrine combined with ulinastatin in the treatment of septic shock. Pak J Pharm Sci. 2022;35(2(Special)):657-63.

751. Yuan K, Wang L. Efficacy of ulinastatin combined with alanyl glutamine for patients with sepsis. INTERNATIONAL JOURNAL OF CLINICAL AND EXPERIMENTAL MEDICINE. 2020;13(2):974-80.

752. Fang Q, Zhao X. Clinical effect of combined ulinastatin and continuous renal replacement therapy on management of severe sepsis with acute kidney injury. Tropical Journal of Pharmaceutical Research. 2017;16(4):925-30.

753. Chen H, He MY, Li YM. Treatment of patients with severe sepsis using ulinastatin and thymosin alpha1: a prospective, randomized, controlled pilot study. Chin Med J (Engl). 2009;122(8):883-8.

754. Huang SW, Chen J, Ouyang B, Yang CH, Chen MY, Guan XD. Immunotherapy improves immune homeostasis and increases survival rate of septic patients. Chin J Traumatol. 2009;12(6):344-9.

755. Huanling S, Rongju L, Min J. Effect and analysis of ulinastatin combined with thymosin on cardiopulmonary function and delirium in sepsis patients. Pak J Pharm Sci. 2019;32(3 Special):1281-4.

756. Li Y, Chen H, Li X, Zhou W, He M, Chiriva-Internati M, et al. A new immunomodulatory therapy for severe sepsis: Ulinastatin Plus Thymosin {alpha} 1. J Intensive Care Med. 2009;24(1):47-53.

757. Yadav AK, Singh VK, Singh GP, Singh V. Outcome of Ulinastatin vs Metabolic Resuscitation using Ascorbic Acid, Thiamine and Glucocorticoid in Early Treatment of Sepsis- A Randomised Controlled Trial. Journal of Clinical & Diagnostic Research. 2021;15(5):36-9.

758. Permana SA, Hartono H, Purwanto B, Indarto D. Non inferiority trial of Channa striata extract on endothelial glycocalyx layer protection in septic patient: a prospective cohort study. Pharmacia. 2023;70(3):569-73.

759. Scorcella C, Domizi R, Amoroso S, Carsetti A, Casarotta E, Castaldo P, et al. Pharmacogenetics in critical care: association between CYP3A5 rs776746 A/G genotype and acetaminophen response in sepsis and septic shock. BMC Anesthesiol. 2023;23(1):55.

760. Heemskerk S, Masereeuw R, Moesker O, Bouw MP, van der Hoeven JG, Peters WH, et al. Alkaline phosphatase treatment improves renal function in severe sepsis or septic shock patients. Crit Care Med. 2009;37(2):417-23, e1.

761. Pickkers P, Heemskerk S, Schouten J, Laterre PF, Vincent JL, Beishuizen A, et al. Alkaline phosphatase for treatment of sepsis-induced acute kidney injury: a prospective randomized double-blind placebo-controlled trial. Crit Care. 2012;16(1):R14.

762. Pickkers P, Snellen F, Rogiers P, Bakker J, Jorens P, Meulenbelt J, et al. Clinical pharmacology of exogenously administered alkaline phosphatase. Eur J Clin Pharmacol. 2009;65(4):393-402.

763. Elbaradey GF, Elshmaa NS, Hodeib H. Role of edaravone in managemant of septic peritonitis. J Anaesthesiol Clin Pharmacol. 2016;32(4):465-9.

764. Frass M, Linkesch M, Banyai S, Resch G, Dielacher C, Löbl T, et al. Adjunctive homeopathic treatment in patients with severe sepsis: a randomized, double-blind, placebo-controlled trial in an intensive care unit. Homeopathy. 2005;94(2):75-80.

765. Pahlavani N, Malekahmadi M, Sedaghat A, Rostami A, Alkadir OKA, Taifi A, et al. Effects of Melatonin and Propolis Supplementation on Inflammation, Oxidative Stress, and Clinical Outcomes in Patients with Primary Pneumosepsis: A Randomized Controlled Clinical Trial. Complement Med Res. 2022;29(4):275-85.

766. Memis D, Karamanlioglu B, Yuksel M, Gemlik I, Pamukcu Z. The influence of methylene blue infusion on cytokine levels during severe sepsis. Anaesth Intensive Care. 2002;30(6):755-62.

767. Emet S, Memis D, Pamukçu Z. The influence of N-acetyl-L-cystein infusion on cytokine levels and gastric intramucosal pH during severe sepsis. Crit Care. 2004;8(4):R172-9.

768. Najafi A, Mojtahedzadeh M, Ahmadi KH, Abdollahi M, Mousavi M, Chelkeba L, et al. The immunological benefit of higher dose N-acetyl cysteine following mechanical ventilation in critically ill patients. Daru. 2014;22(1):57.

769. Paterson RL, Galley HF, Webster NR. The effect of N-acetylcysteine on nuclear factor-kappa B activation, interleukin-6, interleukin-8, and intercellular adhesion molecule-1 expression in patients with sepsis. Crit Care Med. 2003;31(11):2574-8.

770. Pinder N, Bruckner T, Lehmann M, Motsch J, Brenner T, Larmann J, et al. Effect of physostigmine on recovery from septic shock following intra-abdominal infection - Results from a randomized, double-blind, placebo-controlled, monocentric pilot trial (Anticholium® per Se). J Crit Care. 2019;52:126-35.

771. Guntupalli K, Dean N, Morris PE, Bandi V, Margolis B, Rivers E, et al. A phase 2 randomized, double-blind, placebo-controlled study of the safety and efficacy of talactoferrin in patients with severe sepsis. Crit Care Med. 2013;41(3):706-16.

772. Vincent JL, Marshall JC, Dellinger RP, Simonson SG, Guntupalli K, Levy MM, et al. Talactoferrin in Severe Sepsis: Results From the Phase II/III Oral tAlactoferrin in Severe sepsIS Trial. Crit Care Med. 2015;43(9):1832-8.

773. Wu Z, Zhang X, Cai T, Li Y, Guo X, Zhao X, et al. Transcutaneous auricular vagus nerve stimulation reduces cytokine production in sepsis: An open double-blind, sham-controlled, pilot study. Brain Stimulation. 2023;16(2):507-14.
